# Structured evaluation of rodent behavioral tests used in drug discovery research

**DOI:** 10.3389/fnbeh.2014.00252

**Published:** 2014-07-22

**Authors:** Anders Hånell, Niklas Marklund

**Affiliations:** ^1^Department of Neuroscience, Section for Neurosurgery, Uppsala UniversityUppsala, Sweden; ^2^Department of Anatomy and Neurobiology, Virginia Commonwealth University School of MedicineRichmond, VA, USA

**Keywords:** animal behavior, translational medicine, phenotyping, mice, rats

## Abstract

A large variety of rodent behavioral tests are currently being used to evaluate traits such as sensory-motor function, social interactions, anxiety-like and depressive-like behavior, substance dependence and various forms of cognitive function. Most behavioral tests have an inherent complexity, and their use requires consideration of several aspects such as the source of motivation in the test, the interaction between experimenter and animal, sources of variability, the sensory modality required by the animal to solve the task as well as costs and required work effort. Of particular importance is a test’s validity because of its influence on the chance of successful translation of preclinical results to clinical settings. High validity may, however, have to be balanced against practical constraints and there are no behavioral tests with optimal characteristics. The design and development of new behavioral tests is therefore an ongoing effort and there are now well over one hundred tests described in the contemporary literature. Some of them are well established following extensive use, while others are novel and still unproven. The task of choosing a behavioral test for a particular project may therefore be daunting and the aim of the present review is to provide a structured way to evaluate rodent behavioral tests aimed at drug discovery research.

## Introduction

Charles Darwin may be considered to be the founder of behavioral research (Thierry, 2010). Since then, behavioral testing has been extensively used to gain a better understanding of the central nervous system (CNS) and to find treatments for its diseases. Early experimental work on animal behavior includes Ivan Pavlov’s work on conditional reflexes in dogs which began at the end of the 19th century (Samoilov, 2007). Continued interest in animal behavior gave rise to the field of ethology which resulted in the Nobel Prize in Physiology and Medicine in 1973 shared by Karl von Frisch, Konrad Lorenz and Nikolaas Tinbergen. They studied animals in their natural habitat, which made controlled experiments difficult. This problem was addressed by introducing behavioral testing in a laboratory setting in the early twentieth century, which evolved into the field of comparative psychology in a process facilitated by the important contributions made by B F Skinner (Gray, 1973; Dews et al., 1994).

**Figure 1:**
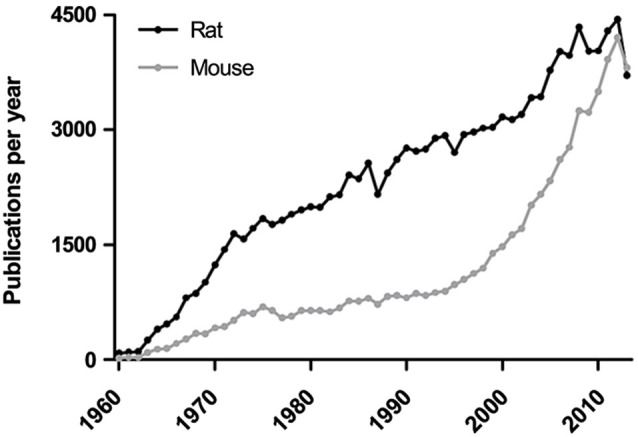
**The total use of mice and rats in behavioral testing**. The number of publications were determined in PubMed using the search terms (mouse or mice) and (rat or rats) combined with (behavior or behaviour), where data prior to 1960 is excluded since the absence of abstracts in the older literature makes search results unreliable. A sharp rise in mouse behavioral testing can be seen in the last decade and is now slightly more prevalent than rat behavioral testing.

Historically, a large variety of species has been used for behavioral testing but rodents have always been widely used, likely since they are mammals and easy to house and breed. In contrast to common pets such as cats and dogs, there may also be a higher acceptance in the general public for the use of rodents in medical research. Although hamsters, guinea pigs and Mongolian gerbils have been subjected to behavioral testing, mice and rats are far more popular and firmly established as model organisms with several outbred stocks and inbred strains available for experiments. Unlike other rodents used for research, mice and rats belong to the subfamily Murinaea and are sometimes referred to as murine models. Early examples of rodent behavioral testing include Karl Lashley’s work on learning and memory using mazes in the early twentieth century. Initially, wild-caught rodents were used for experiments (McCoy, 1909) but this practice changed with the introduction of strains bred by mouse and rat fanciers (Steensma et al., 2010). Following approximately a hundred years of breeding, contemporary laboratory animals are now considerably more docile than their wild counterparts (Wahlsten et al., 2003a). Over time, there has been a continuous evolution of rodent behavioral tests and there are well over 100 tests in contemporary use, exhaustively summarized in Supplementary Table 1 of the present review. In recent years, genetically modified mice have become readily available and the use of mice in behavioral testing recently surpassed that of rats (Figure 1). However, the increasing availability of genetically modified rats (Jacob et al., 2010) may shift the tide again.

Unfortunately, rodent behavioral testing in the laboratory setting have proved difficult and test results may vary depending on the person performing the experiment (Chesler et al., 2002), in which laboratory the experiments are performed (Crabbe et al., 1999) and environmental factors including for example animal housing (Richter et al., 2010). To further advance the field of rodent behavioral testing and achieve reliable and reproducible results, these issues must be resolved. Fortunately, recent technological improvements facilitate the design and construction of sophisticated automated test equipment. New techniques include 3D printing which can construct structures of almost any shape (Jones, 2012), user friendly electronic microcontrollers such as Arduino boards, which can control gates, sensors and reward delivery (D’Ausilio, 2012), as well as Radio Frequency Identification (RFID), which is used to detect the position and identity of individual rats and mice (Lewejohann et al., 2009). Combined with sound ethological principles and a good understanding of rodent biology, these techniques may facilitate the design and construction of novel behavioral tests with improved characteristics.

Unfortunately, there is no such thing as a perfect behavioral test and the most suitable one has to be chosen depending on the goals of the project. There are, however, a large number of factors to take into account when making this decision. To facilitate the process of finding strengths and weaknesses of existing behavioral test, the present review evaluates a multitude of important aspects of behavioral testing which are summarized in Table 1.

**Table 1:** **Points to consider when evaluating a behavioral test**.

Motivation	How are the animals motivated to perform the task? Is the level of motivation high and stable? Can the source of the motivation interfere with the disease model?
Animal-experimenter interaction	Are the experimenter and the animal in close contact during testing? Can handling be performed prior to testing to allow the animals to adjust to human contact?
Dynamic range	Can the test make accurately measure the ability of both naïve and severely impaired animals? Is there a risk for flooring or ceiling effects? Can the test difficulty be adjusted within or between trials?
Repeatability	Can the test be repeated to assess changes in ability over time?
Interaction with other tests	Is there a risk that the test experience can affect the behavior in other test?
Data collection	How are the results collected? Is there a risk for subjective effects in the evaluation? Can the results be collected automatically?
Result evaluation	Which statistical tests can be used to evaluate the results? How are the results presented?
Result interpretation	Can the results be attributed to a single domain or can, for example, changes in general activity level interfere with the measurements?
Automation	How much of the test procedure is automated? Does the test have the potential to be fully automated?
Variability	Can the results be related to baseline performance to mitigate the effects of variability? Can the estrous cycle of female animals be measured to restrict testing to a single day in the cycle?
Experimental design	Can the test be performed in a blinded fashion? Can the test be used for both mice and rats of different strains and stocks to obtain more robust results?
Sensory modality	Which sensory modality does the animal use to solve the task? Is it possible to confirm intact sensory functions?
Predictive validity	Do drugs approved for human use improve test results for rodents?
Construct validity	Does the test rely on brain structures used for this psychological construct in humans?
Ethological validity	Does the test resemble natural rodent behavior?
Face validity	Is it immediately apparent what the test is intended to measure?
Intrinsic validity	Does the test give the same result when experiments are repeated?
Extrinsic validity	Does the test give the same result when performed in, for example, different strains, age group or species?
Housing	Does the test cause restrictions on how the animals can be housed? Can the social status be assessed for group housed animals to allow the creation of balanced experimental groups?
Throughput	How long does it take to run the test? Do the animals have to be trained before performing the test? Can several animals be tested in parallel? Is the collection and evaluation of the results time consuming?
Costs	How much does the equipment cost? How much lab space has to be devoted to the test? How much staff time does the test require?
Practical considerations	Can the equipment be stowed away when not in use? Is extensive training of the experimenter required to carry out the test? Does the test have to be performed on several consecutive days which may overlap with weekends, holidays and vacations? Can the test be run by a substitute in case of sick leave? Can the test equipment be easily cleaned and disinfected?

*The points are ordered as they appear in the text and the importance of each point has to be assessed based on the goals of individual projects*.

## Motivation

Most behavioral tests used to evaluate sensory-motor function as well as learning and memory aim to measure an animal’s ability to solve a task. On the other hand, behavioral tests used in rodent drug dependence research such as self administration and conditioned place preference (see Supplementary Table 1), focus on measuring the motivation to perform a selected action. The measured performance in a test will invariably include a combination of both the ability and the motivation of the animal to solve the task. To detect differences in ability, the level of motivation should therefore be equal among individual animals. To solve the problem of variability in motivation, behavioral tests typically attempt to provide sufficient motivation to make each animal perform at the height of its ability. However, a variable level of motivation is a potential problem in many tests, including the rotarod and grip strength test (Balkaya et al., 2013). A commonly chosen source of motivation is fear, such as fear of drowning in the forced swim test (FST; Porsolt et al., 1977), fear of falling in the rotarod (Dunham and Miya, 1957) or fear of receiving electrical shocks during active avoidance learning (Moscarello and LeDoux, 2013). Fear can be studied independently, using for example predator odors, to gain insights into for instance post-traumatic stress disorder (Staples, 2010; Johansen et al., 2011), which differs from its use as a motivator in tests which evaluate other traits. Fear must be used cautiously as a source of motivator since it may cause unwanted effects such as freezing or panic-like behavior, and fear-induced stress may negatively influence cognitive performance (Harrison et al., 2009). When a test situation is perceived as dangerous to the animal, motivation can also be provided by introducing an escape route to the home cage when the task is solved (Blizard et al., 2006).

Hunger is another commonly used motivator. Rodents typically feed throughout the day although mainly in the beginning and end of the dark phase (Clifton, 2000), and a sufficient level of hunger for motivation is induced at a level of food deprivation which causes a 10–20% reduction in body weight. It must be noted that fear, stress and anxiety inhibit food consumption (Petrovich et al., 2009) and the animals may have to be habituated to the test arena prior to initiation of the experiment. The need for food deprivation might be alleviated or avoided by using a highly palatable food, by using the natural tendency of rats and mice to forage for food and hoard it in their nests (Whishaw et al., 1995) or by relying on thirst rather than hunger. In addition, rodent diet may also influence test results. Free access to food cause obesity in laboratory rodents (Martin et al., 2010) and dietary restriction was observed to be beneficial in models of stroke, addiction and excitotoxicity (Bruce-Keller et al., 1999; Yu and Mattson, 1999; Guccione et al., 2013).

**Figure 2:**
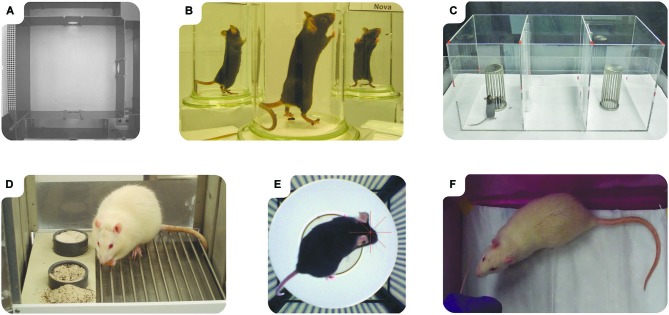
**Recently introduced behavioral tests**. **(A)** Multivariate concentric square field: the animal is allowed to freely explore a complex arena made up of several subdomains with different characteristics. The dark corner room in the top right of the image is for example likely perceived as safe unlike the brightly lit bridge on the left side which is probably considered risky. The result is analyzed using multivariate statistics to give a behavioral profile of the animal rather than attempting to measure individual traits. **(B)** Cylinder test: when the animal is placed in the cylinder it will spontaneously rear and use its forepaws for support. Unilateral injury to CNS motor control areas typically induces asymmetric forelimb use in this task. **(C)** Three-chamber social approach: the sociability of the test mouse is measured by its tendency to spend time in an empty chamber or a chamber containing another mouse. **(D)** The Dig Task: based on olfactory cues, the animal identifies the correct cup and digs to obtain the reward. **(E)** OptoMotry: the unrestrained animal is placed on a small, elevated, platform surrounded by four monitors displaying a grating pattern. If the animal has sufficient visual acuity, the lateral flow of the grating pattern induces reflexive head movements which are automatically detected by an overhead camera. **(F)** Whisker nuisance task: the experimenters hand is seen in the lower left corner holding the small stick which is used to stimulate the whiskers. Traumatic brain injury, for instance, causes allodynia which can be detected in this test. All images except the cylinder test were kindly provided by other scholars, see Acknowledgments for details.

Several tests rely on spontaneous rodent behavior, such as the exploration of a novel environment in the open field (Hall, 1934), multivariate concentric square field (Figure 2A; Meyerson et al., 2006) and the cylinder test (Figure 2B; Schallert et al., 2000). Furthermore, social interaction tests such as the three-chamber social approach (Figure 2C; Nadler et al., 2004), can be used to evaluate memory function as well as sociability. Other spontaneous behaviors include nest building and burrowing which can be used to assess cognitive function (Deacon, 2012). Relying on spontaneous behaviors may reduce the need of strong motivators and likely attenuates the stress level of the animal in a test in contrast to the release of stress hormones induced by, for instance, the Morris Water Maze (MWM; Engelmann et al., 2006). Motivation to repeatedly perform simple tasks may be provided by operant conditioning. This can, for example, be used to motivate animals to press a lever to receive a sucrose pellet and is often performed in a Skinner box. Operant conditioning does, however, typically require some degree of food deprivation as well as an intact learning ability. It can be assumed that the use of positive motivators is not only ethical but also less likely to result in stress-induced aberrant behaviors. Insufficient interest to perform tasks without a strong motivator is still a potential caveat in behavioral testing though this problem might be mitigated by the longer test sessions enabled by automated testing (see *Automated testing* below).

## Animal-experimenter interaction

In virtually all behavioral tests, there is a degree of interaction between experimenter and animal which potentially influences the obtained results. The importance of this interaction was established by the observation that experimenter identity had a greater influence than genotype on hot plate test results (Chesler et al., 2002). Although different interpretations of test instructions might be one explanation, differences between researchers in their amount of animal work experience and anxiety towards rodents is also likely to be important. It has also recently been demonstrated that the presence of a male, but not female, experimenter induce analgesia in rodents (Sorge et al., 2014). In addition, rats are able to distinguish between, and results may be affected by the level of rodent familiarity with, the individual experimenters (McCall et al., 1969; van Driel and Talling, 2005) and any remaining odor traces from predatory pets such as cats will induce stress in rodents (Burn, 2008). Individual human experimenters may also display some day to day variation in for example stress level, mood and/or odor, which potentially increases the variability of the results. Individual rodents also differ in their response to humans and typically initially avoid human contact but gradually accept it following repeated exposure (Schallert et al., 2003; Hurst and West, 2010). Handling of laboratory animals prior to any behavioral testing may therefore reduce the effects of animal-experimenter interaction and potentially reduce variability (Schmitt and Hiemke, 1998; Hurst and West, 2010). Additionally, lack of handling before a series of repeated testing may cause altered results over time as the animals get more and more used to human contact. Note, however, that human presence always influences animal behavior and reduced fear of the experimenter may even decrease the motivation to perform some tests. Animal-experimenter interaction is not limited to fear reactions, and may also be caused by curiosity and the anticipation of reward. The importance of animal-experimenter interaction likely differs between tests, and is likely of little concern in for example operant chambers, while it potentially significantly affects several tests of neurological function where the animal is held by the experimenter throughout the test.

In the attempts made to evaluate reproducibility between different laboratories the experiments are usually performed by different persons (Crabbe et al., 1999; Mandillo et al., 2008), with experimenter identity reported as an important source of variability (Mandillo et al., 2008). Animal-experimenter interaction may thus be one of the reasons for the difficulties in achieving consistent results in behavioral tests. By relying on fully automated testing (see *Automated testing* below) human contact is avoided and the reproducibility of the test is potentially increased. Since fully automated, high quality, behavioral test are still sparse, the problem of animal-experimenter interaction is instead typically addressed by having the same person perform all testing within a study.

## Range of reliable measurements

**Figure 3:**
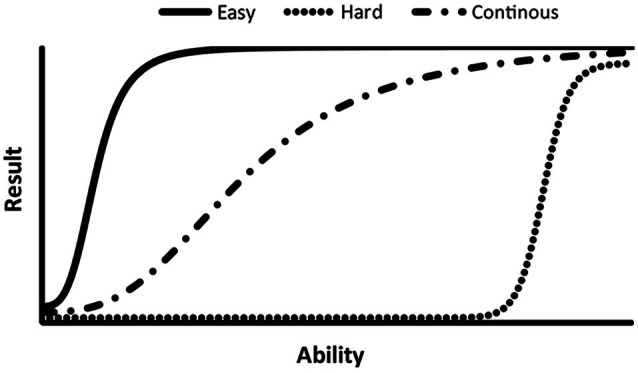
**The level of difficulty varies between rodent behavioral tests which makes them suitable for different purposes**. A non-demanding test (solid line) is, for example, suitable for detection of treatment effects in models of severe central nervous system lesions. Non-demanding tests may, however, display ceiling effects, i.e., even impaired animals receives close to optimal test results. A highly demanding test (dotted line), on the other hand, cause the risk of flooring effects where all animals fails the task, leaving any improvement undetected. A demanding test is thus mainly suitable for detection of minor insults, such as side effects of treatments or discrete effects of genetic manipulations. Test with a continuous increase in difficulty or stimulus intensity (mixed line) are useful over a wide range of ability/trait levels, i.e., have a wide dynamic range. See *Range of reliable measurements* in the text for examples.

A behavioral test should ideally enable accurate and precise measurements without flooring and ceiling effects (Figure 3) when testing both highly impaired and normally functioning animals. A reliable assessment over a wide range of ability levels can be achieved by continuously increasing the difficulty or stimulus intensity during a test session, such as protocols using accelerating, rather than constant, speed in the rotarod. In this test, the rotating drum starts at a very slow speed, demanding only for severely impaired animals, and then accelerates up to a speed challenging even for naïve mice (Dunham and Miya, 1957; Brooks and Dunnett, 2009). The same principle is applied in the ledged tapered beam, a modification of traditional beam walking tests. Here, the beam is initially wide and easy to traverse but gradually tapers and becomes increasingly narrow and challenging (Schallert et al., 2002). This approach to test design is not limited to motor function tests and is for example also used in the successive alleys test of anxiety-like behavior. This test consists of four alleys which are increasingly narrow and open, and thus more anxiogenic, to enable assessment of anxiety-like behavior over a wide range (Deacon, 2013). The demands of a test can also be controlled using parameters within the test, for example by adjusting the platform size in the MWM (Vorhees and Williams, 2006) or by changing the temperature in the hot plate test (Neelakantan and Walker, 2012). Correct parameter settings are important since differences between two groups cannot be detected if a test is either too difficult or too easy (Figure 3).

## Repeatability and interaction between tests

Repeated testing is desirable in the study of diseases with a dynamic and prolonged course as well as in development and ageing research. The measurement of baseline performance also allows treatment groups to be equally balanced based on performance prior to, for example, drug administration or injury induction (Lenzlinger et al., 2005). Repeating a test may, however, not always be possible since the experience of one test session may influence subsequent testing. Most behavioral tests are likely affected to some extent by repeated testing, and practice effects have for example been observed in the zero maze (Cook et al., 2002) and the hot plate test (Espejo and Mir, 1994). Repeated testing can also be influenced by the test interval, demonstrated by the functional recovery seen with daily, although not weekly, assessment in the rotarod following traumatic brain injury (O’Connor et al., 2003). These results also suggest that intensive behavioral testing can function as rehabilitation therapy which would cause the testing itself to influence the obtained results. Furthermore, learning effects may influence the results when tests are repeated and impairments in learning and memory may thus influence the evaluation of other brain functions. Test repeatability is also desirable when extensive training prior to the actual testing is required, an approach validated in for example the 5-choice continuous performance test (Young et al., 2013).

Apart from repeating a single test, animals are also commonly subjected to several different behavioral tests. This practice may be problematic since participation in one test potentially influences the results obtained from subsequent testing. Thus, the order in which the tests are carried out is important and performing the tests on separate days potentially reduces the interaction between tests. However, this strategy has to our knowledge not been verified. A possible solution may be to combine several tests into a single test. For instance has the open field, elevated plus maze (EPM) and light-dark box tests been integrated into a single test arena (Ramos et al., 2008). Another strategy is to use a test that does not measure a single behavioral trait in the animal, but instead determines a behavioral profile. An example of this strategy is the multivariate concentric square field test, which uses a complex arena to enable simultaneous evaluation of several aspects of rodent behavior and subsequent analysis of the results using multivariate statistics (Meyerson et al., 2006; Ekmark-Lewén et al., 2010). Another example is a modified version of the hole board test, which measure several behaviors related to anxiety-like behavior, cognition and social interactions (Ohl and Keck, 2003).

## Data collection and result interpretation

Specific rodent behaviors are typically difficult to describe using a single continuous variable and categorical scales are therefore commonly used. Manual scoring using this type of scales may be subjective and should therefore be performed by a researcher blinded to the treatment, disease and genetic status of the evaluated animal. Preferably, the evaluation should also be preceded by an evaluation of the inter- and intra-rater reliability (Shrout and Fleiss, 1979; Rousson, 2011). The use of automated data collection such as video tracking software for mazes and the use of hind paw attached magnets to measure inactivity in the FST (Shimamura et al., 2007), is not always feasible but may assure objective data sampling and likely reduces the work load (see *Automated testing* below). One caveat caused by automated data collection is the increased risk of false positive findings when evaluating a large number of behavioral variables from one or several tests. Proper use of corrections for multiple testing, multivariate statistics, a clearly defined hypothesis and replication of key findings in independent experiments are potential solutions for this problem.

When behavioral test results have been collected, the interpretation of them is rarely obvious. For instance, mice which floats in a single location in the MWM fails to find the platform although this behavior may not reflect impairments in learning and memory capacity (Wahlsten et al., 2003c). Additionally, if a rodent clings onto the rod in the rotarod test instead of running on top of it, no conclusions about its balance and coordination can be made (Wahlsten et al., 2003b). In the interpretation of behavioral test results, an important issue is to understand the cause of the observed behavior. By studying natural rodent behavior, evaluating the ethological validity of test (see *Validity* below), determining the source of motivation in the test (see *Motivation* above) and using the knowledge of rodents’ sensory capacity (see *Sensory modality* below) to view the test from a rodent’s perspective, increased understanding of the rodent behavior in a test may be achieved.

Redesigning existing tests can potentially resolve interpretation issues in some cases. One such example is the elevated zero maze, a modification of the EPM, in which the center zone has been removed since the amount of time spent in it is difficult to interpret (Shepherd et al., 1994; Braun et al., 2011). Reduced levels of paw licking and escape behaviors in the hot plate test can be attributed to both anesthetic, anxiolytic and sedative drug effects (Yezierski and Vierck, 2011; Casarrubea et al., 2012). A potential solution to this interpretation issue is to use the double plate test where the animal is allowed to freely explore two plates, one plate at room temperature and the other maintained at an aversive temperature. The fraction of the time spent on each plate may then be used to quantify anesthetic effects (Walczak and Beaulieu, 2006). High variability is another common problem in behavioral testing, likely mainly caused by factors outside the test situation such as social status and past experiences of the animal. However, some of the variability may be caused by imprecise measurements and more extensive testing may mitigate this problem. For example, the evaluation of more rears in the cylinder test or performing more test runs in the beam walk test may increase the reliability of these tests. Behavioral test sessions often also have a limited duration, typically only a few minutes, which may be insufficient. Accordingly, altered behavioral patterns have been observed when using extended test session durations in the open field test (Fonio et al., 2012). Although beyond the scope of the present review, adequate use of statistics is obviously crucial in behavioral testing and a detailed discussion about core concepts can be found in the *Points of significance* article series (Krzywinski and Altman, 2013). When interpreting beneficial pre-clinical treatment effects, it is, however, also important to not only consider the statistical significance of the effect but also if the magnitude is sufficient to translate into a clinically meaningful improvement. Finally, if behavioral test results are described with a single, easily interpreted variable, it is easier for other scientist to draw correct conclusions from them. However, this strategy has to be balanced against the benefit of a detailed description of the observed behavior.

## Automated testing

The continuous advancement in electronics and image analysis makes automated behavioral testing procedures increasingly feasible. The use of automated procedures has several potential advantages such as objective scoring, avoidance of animal-experimenter interaction, extended test durations and reduced work effort. Fully automated systems are, however, still sparingly used although automated data collection is rather common (see *Data collection and result interpretation* above) and operant chambers typically only require the animal to be placed in the testing chamber while the rest of the procedure is automated. Simultaneous tracking of individual animals within a group can be achieved by the application of different fluorescent dyes to the animals’ fur which allows automated evaluation of social dynamics over extended time periods (Shemesh et al., 2013). As previously correctly pointed out (Crabbe and Morris, 2004), it is unfeasible to design automated tests which rely on a robotic system to capture and ferry animals from their home cage to the test arena. Fully automated testing may instead be carried out in the animals’ home cage (de Visser et al., 2006; Krackow et al., 2010) or in a test module attached to the home cage via an automated sorting system (Schaefers and Winter, 2011; Winter and Schaefers, 2011). Integration of the test equipment and home cage into a single unit is for example used in the IntelliCage (Krackow et al., 2010) and the PhenoTyper (de Visser et al., 2006). This strategy does, however, have certain limitations. The test equipment can, for example, only be used by one group of animals at a time, and to test a group of animals in several different in-cage test systems, the animals have to be transferred between them. These limitations may be resolved by adding an automated sorting system between the home cage and the test arena, a strategy which has been successfully implemented for both an operant chamber (Winter and Schaefers, 2011) and an automated T-maze (Schaefers and Winter, 2011). In this setup, the animals are tagged using RFID-chips which can be identified by the sorting system which make sure that only one animal at a time enters the test arena. This approach can potentially be extended by connecting several cages to the same test arena or by connecting one cage to several test arenas.

The creation of fully automated tests which precisely mimic currently used tests is, however, an unconceivable task in most cases. Instead, thinking along new lines may be required to allow computers to make measurements and control the test situation. An elegant example is the automated assessment of grooming behavior, where the vibrations caused by the grooming are detected using a highly sensitive scale and translated to meaningful information using advanced algorithms (Chen et al., 2010). Attempting to mimic a human observer by relying on image analysis of video-captured grooming behavior is considerably more difficult, even though machine learning potentially makes this possible (Kabra et al., 2013). Furthermore, rodents are stressed by human contact and avoiding it would therefore be an example of refinement in the 3R (Replacement, Reduction and Refinement) system (Russell and Burch, 1959). Finally, regardless of how tempting it can be to use automated tests to speed up the work process, obtaining high throughput at the expense of high quality may result in unreliable data of limited value. Frequently it may, therefore, be advantageous to use a more labor intensive test to achieve a higher validity and increased chance of successful clinical translation.

## Variability

Rodent behavior usually varies considerably from animal to animal, likely caused by a combination of genetic, environmental and experimental factors. Variability may be present even prior to the initiation of an experiment, for example, when using outbred animals which are meant to be genetically diverse. Outbred rat stocks are frequently used and include, for example, Sprague-Dawley, Long-Evans and Wistar rats. Although the mice used in medical research usually are inbred, the CD1 and Swiss Webster mice are outbred and it is reasonable to assume that the genetic diversity in outbred animals contributes to behavioral variability (Chia et al., 2005). All individuals in an inbred strain are meant to be genetically identical but may still differ in their minisatellite regions, short repetitive DNA sequences with highly polymorphic copy numbers, which potentially affect gene expression and behavior (Lathe, 2004). Since variability in preferred activities potentially alters life experiences, variability may also increase over time (Freund et al., 2013).

Maternal behavior also varies between individuals and high levels of pup licking and grooming leads to altered stress reactivity and improved performance in the MWM when the pups reach adulthood (Liu et al., 2000). Maternal behavior is also inherited in a non-genomic fashion making it a possible source of variability affecting future generations (Meaney, 2001). Rodent embryos excrete sex hormones in the uterus where they are lined up like beads on a string. This means that the sex hormones an embryo is exposed to depends on the sex of the adjacent embryos, which potentially affects adult behavior (Ryan and Vandenbergh, 2002). Rodents have a well-developed immune system and obvious symptoms of post-surgery infections are rare. However, infection causes an immune response, which may affect experimental outcome and increase variability. For instance, infection following surgery was shown to exacerbate ischemic brain injury (Yousuf et al., 2013). The composition of commensal bacteria in the rodent intestine can also influence behavior adding yet another potential source of variability (Foster and McVey Neufeld, 2013).

The estrous cycle of female animals also potentially affects behavior (Meziane et al., 2007), although it may fortunately be measured fairly easily using vaginal swab cytology (Caligioni, 2009). Female mice previously unexposed to male mice coordinate their estrous cycle when exposed to male pheromones, the Whitten effect (Whitten, 1956). By introducing male urine-soaked bedding into a cage with female mice for a few days, experiments can be performed using individuals in the same estrous stage (Dalal et al., 2001). The commonly held belief that female animals have a higher variability due to their estrous cycle has recently been questioned after a meta-analysis found that males and females had similar levels of variability on a wide range of outcome measures (Prendergast et al., 2014).

Experimental disease models often require surgery or substance administration and the level of sustained injury or disease severity may vary from subject to subject (Kim et al., 2011). The dose of administrated drug and the resulting plasma level may also vary between individuals (Kääriäinen et al., 2011). Variability may also be introduced during the actual testing, for example by the order in which the animals in a cage are tested (Chesler et al., 2002), and test order should therefore be randomized. The effects of variability may be limited by measuring behavior before an intervention to allow animals with aberrant behavior to be excluded as well as enabling presentation of the results normalized to the baseline value. Since variability reduces the ability to detect significant differences, the identification and removal of sources of variability is important in the development and refinement of behavioral tests and testing procedures. Since decreasing variability enables smaller experimental group sizes it is not only practical but also ethical since it reduces the number of animals used (Russell and Burch, 1959).

## Experimental design

The risk of bias is ever present in medical research and failure to take measures to avoid it resulted in overestimated treatment effects in preclinical stroke trials (Sena et al., 2007). Several guidelines outlining important measures to avoid bias have been prepared including the Camrades and ARRIVE guidelines (Kilkenny et al., 2010). Particularly, the assessment of animal behavior risks being subjective and should always be performed by a person blinded to the genotype, surgical intervention or drug treatment. Given the possibility for animal-experimenter interactions, it is also prudent to perform actual testing in a blinded manner. If the person performing genotyping, surgery or treatment administration is also performing behavioral testing, blinding becomes a practical problem. If the animals are unlabeled prior to the intervention, it may be possible to have them labeled by another researcher afterwards. Replacement of cage cards may also be a possible way to conceal group assignment in some cases. RFID tags used to identify animals contain a number which is paired with the text displayed on the RFID reader, which means that blinding can be achieved by pairing the number with a new text. Group size is another important aspect of the experimental design and it has been suggested that neuroscience studies commonly use an insufficient number of animals. Power calculations are therefore recommended to determine adequate group sizes (Button et al., 2013).

Group assignment would not be an issue if all rodents in an experiment were identical. However, given the arguments in the section on variability (see *Variability* above), individual rodents are likely unique and randomization is required to avoid bias. However, unrestricted randomization may lead to, for instance, a cage containing only control animals, which introduces a risk of systematic errors. This risk can be avoided by dividing the study into blocks consisting of, for example, a single litter or a cage of animals and then randomly allocate the animals in each block to treatment groups (Festing and Altman, 2002). Given the importance of blinding and randomization, it is recommended to always describe it in scientific reports (Macleod et al., 2009).

Most rodent behavioral testing is done to understand human physiology and to find treatments for diseases, and experiments should therefore be designed to maximize the potential for successful translation of the results into patient benefit. It seems reasonable that a drug treatment which is effective only in single strain during a narrow age span is unlikely to translate into an effective clinical treatment. The chance for successful translation is on the other hand likely higher for treatments which are effective over a wide range of parameters like species, strain, sex, age and environmental factors. Evaluating treatments in the traditional way with a treatment group and a control group for each combination of parameters would unfortunately be prohibitively expensive and time consuming. By instead using a pair of animals for each set of parameters, where one animal per pair is given the evaluated treatment and the other serves as the control, robust effects could be detected without using large amounts of animals. Since performance is likely to be influenced by numerous factors, in particular the strain, the results would have to be evaluated using paired non-parametric statistics. This way of using a paired design to test animals of different species, strains, ages and sex within a single study has to our knowledge not previously been evaluated. It may, however, be a way to avoid the risk of obtaining idiosyncratic results caused by using inbred animals of a single age and sex which are housed and raised under identical conditions.

## Sensory modality

To fully understand animal behavior, it is crucial to recognize that their view of the world can differ drastically from ours (Burn, 2008). Extreme examples include the sea turtles reliance on the earth’s magnetic field for navigation and the ability of various snake species to detect their prey using infrared radiation. Rodents perceive their environment primarily by using their excellent olfactory system and vomero-nasal organ and do not, unlike humans, rely heavily on vision (Ache and Young, 2005). Rodent vision is adapted to a nocturnal lifestyle with a dominance of rods in the retina. Furthermore, both rats and mice only have two types of cones limiting them to dichromatic vision, though one type of cones enables detection of ultraviolet light (Huberman and Niell, 2011). Rodents also have highly developed whiskers with large cortical representation, and actively move their whiskers over objects to examine them (Diamond et al., 2008). Another difference between human and rodent sensory systems is the ability of rodents to both detect ultrasound and use it for communication (Wöhr et al., 2008, 2011). It has also been suggested that mice can detect magnetic fields (Muheim et al., 2006) and that rats are able to echolocate (Rosenzweig et al., 1955).

Vision-based rodent behavioral test can closely mimic clinical test procedure which has been suggested to facilitate the translation of pre-clinical test results to the clinic (Horner et al., 2013), while others have cautioned against adopting an anthropocentric view (Wynne, 2004). Olfaction based test procedures are an alternative which may have better ethological validity (see *Validity* below). Such tests are still infrequently used relative to other types of behavioral tests, but there are for example several procedures where the animals dig for rewards in scented media (See Supplementary Table 1). One of them is the Dig Task (Figure 2D; Martens et al., 2013), although several other protocols are available for this versatile cognitive evaluation system, where reward location can be indicated not only by the scent and type of the digging medium but also the surface structure and location of the containers (Wood et al., 1999; Birrell and Brown, 2000; Gilmour et al., 2013).

Other factors to consider are that several commonly used mouse strains have restricted hearing abilities and that the barren environment in which laboratory animals are reared can impair sensory development (Cancedda et al., 2004; Turner et al., 2005). Accordingly, it is a desirable quality control for any behavioral test to verify that the animals have sufficient sensory capacity to detect the provided sensory cues. Such sensory evaluation is of particular importance when using genetically modified animals which may have unexpected sensory deficits as well as in disease models which may alter sensory functions. Fortunately, visual function can be assessed automatically using the Optomotry system (Figure 2E; Prusky et al., 2004), anosmia can be rapidly detected using the buried food test (Yang and Crawley, 2009) and hearing can be evaluated using pre-pulse inhibition of the acoustic startle response (Clause et al., 2011). The whisker sensory system can also be assessed using the Whisker nuisance task (Figure 2F; McNamara et al., 2010). Detailed assessment of sensory functions can also be performed using operant conditioning procedures (See Supplementary Table 1).

## Test validity

There are several types of validity which have to be considered when evaluating behavioral test results. The predictive validity is arguably one of the most important and is typically defined as the ability of a rodent behavioral test to predict the effect of a drug in humans. Determining the predictive validity requires an existing clinical treatment which can be back-tested in the rodent behavioral test under evaluation (Schallert, 2006). An example is benzodiazepines, which are widely used to treat anxiety in patients and also reduce the extent of rodents’ anxiety-like behavior in both the light-dark box test (Crawley and Goodwin, 1980) and the EPM (Pellow and File, 1986). Predictive validity can, however, frequently not be assessed due to the lack of available efficient treatments and construct validity (Strauss and Smith, 2009) may be used instead. This type of validity depends on whether the test measures the intended psychological construct or not and may for example be assessed by evaluating whether the involved neural systems and neurotransmitters are similar in rodents and humans.

In a test with high face validity it is obvious what the test is intended to measure. The rotarod test is for example easily and immediately recognized by most scientists as a motor function test. The ethological validity on the other hand depends on how well the test resembles natural animal behavior. It is for example easy to see how the use of predator odors to elicit fear responses resembles a situation which may be experienced by wild rodents as well. On the contrary, mice suspended by their tails are not observed in nature, although the tail suspension test is commonly used as an indicator of depression-like behavior (Cryan et al., 2005).

The internal validity, reproducibility, of a test depends on the similarity of results obtained from repeated experiments. The external validity, generalizability, of a test is determined by its ability to predict results in other strains and species, including humans. Standardization of behavioral tests and testing environment may increase internal validity although it may, on the other hand, decrease the external validity (Würbel, 2002; van der Staay and Steckler, 2002). Though standardization of test parameters may increase reproducibility, the choice of parameter setting is not always obvious and optimal settings for one disease model is not necessarily ideal in models of other diseases. Systematic variation of test parameters may on the other hand increase the external validity (Richter et al., 2010) and thus increases the chance of successful translation of the result to the clinic.

Using a test with a high predictive validity is likely the best choice if available. There is, however, a risk that the demonstrated validity only holds for other drugs of the same class. Relying on untried behavioral tests might on the other hand lead to the discovery of drugs acting via novel mechanisms. If fully validated tests are not available, a potential alternative to increase the reliability of the conclusions is to measure a trait using several different tests in the same domain. Tests and testing procedures may also be validated by verifying that known strain differences and/or drug effects can be replicated. In drug discovery research it is also important to separately consider the validity of the disease model to determine the reliability of the obtained test results.

## Animal housing

Housing is crucial when testing behavior and may influence the results in several ways. For example, rodents within a cage will form a social hierarchy where a lower rank is associated with increased levels of stress hormones and anxiety-like behavior, potentially adding to behavioral variability (Barnum et al., 2008; Costa-Pinto et al., 2009; Davis et al., 2009; Prendergast et al., 2014). Several tests are available to measure the social status including barbering, urination patterns, the tube confrontation test, the visible burrow system and the food contest task (Merlot et al., 2004; Wang et al., 2011; see Supplementary Table 1), which allows the formation of balanced experimental groups prior to an experiment. A further complication of group housing is the tendency of male mice to fight and wound each other (Emond et al., 2003), though this is less likely with littermates or among animals who have been sharing a cage since early age (Costa-Pinto et al., 2009). On the other hand, since both mice and rats are social animals, single housing may affect the results in several behavioral tests (Võikar et al., 2005). For the majority of studies, group housing is likely preferable since results obtained using socially deprived single housed animals may have a reduced validity.

Another aspect of rodent housing is the potential inability of a rodent cage to provide sufficient stimuli for normal development. Various forms of enrichment such as plastic tunnels, running wheels, rubber balls and nesting material can be used to mitigate this problem (Nithianantharajah and Hannan, 2006). The effects of enrichment are, however, complex and conditional knock out of a subtype of the NMDA receptor in the hippocampus was found to affect non-spatial memory and learning in mice reared in standard although not in enriched environments (Rampon et al., 2000). Rodent cages must also be cleaned at regular intervals, preferably on days without behavioral testing since the transfer to a new cage transiently increases stress hormones (Castelhano-Carlos and Baumans, 2009). Lightning conditions also needs to be considered since testing at different time points in the light-dark cycle may alter the outcome of behavioral testing (Hopkins and Bucci, 2010). Contrary to humans, rats and mice have their resting period during the day and a reversed light-dark cycle (Bertoglio and Carobrez, 2002) enables testing when the animals are active which is likely to be the best alternative in most behavioral studies.

## Practical considerations

Rodent behavioral tests vary considerably in the required work effort they require, which needs to be balanced against the reliability and importance of the obtained results. There are also ever more efficient methods available for the development of genetic models and antibodies as well as for the quantitation of mRNA synthesis and protein expression levels. If behavioral testing fails to match these improvements, it risks becoming a bottleneck in the drug development process (Brunner et al., 2002). If fully automated systems cannot be implemented, throughput can be increased by testing several animals in parallel, such as when using rotarod devices constructed with several lanes. Test equipment of a small size and reasonable cost also allows the use of several test chambers in parallel to make the work more efficient, a strategy applied for example in fear conditioning testing (Maren, 2008) and when using operant chambers. Relying on test procedures which can be performed by new personnel without extensive training or technical skills is also of practical value (Brooks and Dunnett, 2009). The cost of laboratory premises can also be a substantial part of a research budget, which needs to be considered when planning to use physically large equipment like the MWM or the EPM. Furthermore, if tests are not used continuously, the possibility to disassemble and store test equipment can be of great practical value.

Behavioral tests must frequently be performed at a specific time depending on estrous cycle, time after injury or disease onset, or as part of a project schedule including several batches of animals subjected to several different tests. Such rigorous schemes are often needed, although problematic when faced with sick leave, vacations and holidays. One way to overcome such practical problems is to use fully automated tests or tests with low animal-experimenter interaction which may allow for the testing to be performed by a substitute investigator.

Cleaning of behavioral test equipment serves to avoid the spread of contagious diseases and to remove odor traces which otherwise might influence test results. Apart from obvious olfactory cues like feces and urine, rodents secrete fluids from their foot pads which might influence subsequent testing (Quatrale and Laden, 1968). Rats are also viewed as predators by mice (Quatrale and Laden, 1968) and if the two species share equipment, thorough cleaning is required. To facilitate the cleaning process the equipment should be devoid of narrow passages and corners, made of a readily cleanable material and preferably able to withstand sterilization procedures.

## Concluding remarks

Although genetics, electrophysiology and histology are very important tools for understanding underlying mechanisms of novel drug treatments, behavior represents the final output of the CNS and should be the basis for the final conclusion of preclinical evaluations of novel drugs or genetic modifications. Unfortunately, behavioral testing is very labor intensive as well as sensitive to environmental factors and the translation from preclinical to clinical studies has proven difficult in the fields of stroke, brain trauma, spinal cord injury, pain and Alzheimer’s disease (Lo, 2008; Mao, 2009; Loane and Faden, 2010; Filli and Schwab, 2012; Savonenko et al., 2012). Novel behavioral tests may be needed to overcome this crucial problem but it is at least potentially mitigated by careful selection and execution of existing behavioral tests. Initial considerations prior to behavioral testing include selection of animal species, strain, gender and age as well as a determination of sample size, order of testing, type of housing and whether to use a reversed light-dark cycle or not. The best choice of a test depends not only on the scientific goals of the project, the intended measure and possible interpretations of the results, but also on practical and economical constraints, and may therefore differ between projects. In most cases, however, the ideal test is one which not only measures a clinically relevant trait and has a high validity, but also is practically feasible and ethically acceptable. The finding of such a test may be challenging but the process is potentially facilitated by the evaluation structure presented in Table 1 and the listing of available tests in Supplementary Table 1. Finally, given the incomplete understanding of the brain and the limited treatment options for neurological disorders, rodent behavioral testing will likely continue to evolve and to be an indispensable part of neuroscience for the foreseeable future.

## Conflict of interest statement

The authors declare that the research was conducted in the absence of any commercial or financial relationships that could be construed as a potential conflict of interest.
