# Supplementary material for: Structured evaluation of rodent behavioral tests used in drug discovery research
Source: Front Behav Neurosci. 2014 Jul 22;8:252. doi: 10.3389/fnbeh.2014.00252 (PMC4106406; doi:10.3389/fnbeh.2014.00252)
Supplement: Supplementary file 1 [file Supplementary_Table_1.PDF]

Supplementary Table 1 Behavioral tests in contemporary use. Frequency of use was evaluated for the period 2010-2012 where “Low” means less than five publications/year, “Medium” five to twenty publication/year and “High” more than twenty publications/year. Year introduced refers to the first description of the test in rodents. In some cases, marked with *, a definite first description could not be located, and the oldest description found is used instead. The references include initial descriptions and reviews of the tests as well as publications evaluating parameter settings or contain detailed protocols, pictures or films of the tests. The tests in the table were primarily identified using reviews on functional evaluation in major disease models with additional test identified when searching for references of already identified tests. The list aims to be exhaustive and any omissions are purely unintentional and should not be seen an indication of low quality. Due to the large number of existing test, and the fact that new tests are continuously developed, there may be useful tests which are not mentioned in this table. FOU Frequency of use, Aka, Also known as.

**Test**	**Domain**	**FOU**	**Year**	**Description**
0-maze				See Zero maze
5-choice continuous performance task[1,2]	Attention	Low	2009	In the majority of trials the animal has to nose-poke in one of five possible locations identified by a brief visual stimulus to receive a reward. In a subset of trials all five locations display the visual stimulus and the animal has to withhold action in these trials to receive the reward. Also see 5-choice serial reaction task.
5-choice serial reaction task[3–9]	Attention	Medium	1983	Guided by a brief visual stimulus the animal nose-pokes in one out of five holes and correct choices are rewarded. Since the visual stimulus is only presented briefly, animals with attention deficits will have impaired performances. Also see 5-choice continuous performance task.
Acetone test[10,11]	Nociception	Medium	1994*	The test evaluates the cold-evoked response caused by application of a drop of acetone to the animals' skin, which reduces temperature due to evaporation.
Acoustic startle[12–22]	Reflexive behavior	High	1939*	The startle response induced by a sudden loud acoustic stimulus is assessed. Also see Prepulse inhibition and Tactile startle.
Active avoidance				See One-way active avoidance, Passive avoidance and Shuttle-box
Adhesive removal test[23–26]	Sensory-motor function, Laterality	Medium	1982	The time to initiation and to successful removal of an adhesive tape placed on the animal's forepaws is noted. By using adhesive tape patches of different size for each forepaw, asymmetry caused by unilateral impairments can be graded. Optionally, the adhesive tape is placed on the hind paws or snout. Aka Bilateral tactile stimulation test, Sticky tape test and Sticky paper test.

Angle board				See Inclined plane
Ankle-bend test[27–29]	Nociception	Low	1987*	The amount of squeaks and struggle during paw manipulation is assessed. This test is mainly used in arthritis models. Also see Knee-bend test.
Attentional set-shifting[30–38]	Learning and memory, Cognitive flexibility	Low	1998*	The ability of an animal to locate a buried reward, located in one out of several cups filled with a digging medium, is analyzed. The correct location can be based on the type of digging medium or its scent, cup surface texture or cup location. The protocol can be modified to allow both intra- and extra dimensional set shifts as well as delayed non-matching to sample testing. Also see Dig task, Odor span task and Spatial span task.
Autoshaping[39–43]	Learning and memory	Medium	1974*	When a spatially localized sensory cue is used in Pavlovian conditioning the animal tends to approach and interact with the source of the cue which is utilized in autoshaping procedures. The sensory cue is initiated on a variable schedule and lasts for a set time interval. If the animal interacts with the source of the sensory cue the reward is delivered immediately and otherwise at the end of the preset time interval. These procedures are therefore often described as a mix between Pavlovian and operant conditioning.
Balance beam[44–47]	Sensory-motor function	High	1976*	The time the animal is able to remain on a narrow, elevated beam without falling down is measured. Aka Beam balance, Dowel test and Fixed bar test. Also see Beam walk and Ledged tapered beam.
Barbering[48–53]	Social status, Obsessive-compulsive like behavior	Low	1972*	The behavior of dominant animals to remove whiskers and/or fur from submissive animals is evaluated to determine social status. Excessive barbering may also be interpreted as an obsessive-compulsive like behavior. Aka Whisker trimming and Dalila effect.
Bar test[54–58]	Catalepsy	Medium	1972*	The animal's forepaws are placed on a bar and the time it retains this position is recorded.
Barnes maze[59–64]	Visuo-spatial learning and memory	High	1979	The test is performed using a brightly lit circular arena where one of several openings leads to an escape route. The time required to locate the escape route during repeated testing is used to evaluate learning and memory. Aka Circular platform maze.

Basso, Beattie, Bresnahan (BBB) Locomotor Rating Scale[65–70]	Neurological function, Gait	Medium	1995	A 21 point categorical scale used to assess locomotion in the open field following spinal cord injury. Aka BBB scale. Also see Basso Mouse Scale for Locomotion (BMS).
Basso Mouse Scale for Locomotion (BMS)[71]	Neurological function, Gait	Medium	2006	A 9 point categorical scale used to assess locomotion in mice following spinal cord injury. Also see Basso, Beattie, Bresnahan (BBB) Locomotor Rating Scale.
Baton test[72,73]	Sensory-motor function	Low	2008*	The mouse is held by the tail and the ability to grasp a vertically held applicator stick is evaluated.
BBB scale				See Basso, Beattie, Bresnahan (BBB) Locomotor Rating Scale
Beam balance				See Balance beam
Beam walk[74–77]	Sensory-motor function	Medium	1982*	The time required to traverse a narrow beam to reach a goal box is evaluated. Optionally, vertical pegs may be placed along the beam to increase test difficulty. Also see Balance beam and Ledged tapered beam.
Bederson scale				See Neurological score
Behavioral sensitization				See Locomotor sensitization
Bilateral tactile stimulation test				See Adhesive removal test
Bin cotton use				See Nest building
Block test (catalepsy)[78–80]	Catalepsy	Low	1981*	The animal is placed on top of a small block and the time to descend from it is measured.
Block test (odor)[81–84]	Olfaction, Social interactions	Low	2006*	The time the animal spends investigating a wooden block scented with its own bedding and another block scented with another animal's bedding is measured.
Bowl test				See Rotometer
Bracing test				See Lateral pulsion
Buried food test[85–87]	Olfaction	Low	1971	The latency to locate a food item hidden under bedding material is used to assess olfactory abilities. Aka Buried pellet test.
Buried pellet test				See Buried food test
Burrowing[88–91]	Species-typical behavior	Medium	2001*	The test determines to what extent the animal removes material from a tube to create a burrow.

Capellini handling test				See Pasta handling
CatWalk[92–102]	Gait, Nociception	Medium	1999*	The animal walks on top of a horizontal transparent pane. Light enters the pane from the side and can only leave the pane at points of paw contact which illuminates the paws and the animal can be filmed from below to record the gait pattern. Limb pain may also be detected by this test if it alters the gait pattern. Also see Paw print analysis.
Challenging beam				See Ledged tapered beam
Cheeseboard maze				See Holeboard
Chimney test[103,104]	Sensory-motor function	Medium	1960	The animal is introduced into a narrow horizontal plastic tube. As soon as the animal almost has reached the other end of the tube, it is turned to a vertical position and the animal is now facing head down. The normal response for the animal is to start backing out of the tube.
Circle exit test				See Exit circle test
Circular platform maze				See Barnes maze
Cliff avoidance[105–107]	Developmental stage	Medium	1975*	The time it takes for a pup to back away after it is placed with its head and forelimbs hanging over a cliff is recorded.
Coat hanger test[108–110]	Sensory-motor function	Low	1992*	The ability to remain on or climb to the top of a wire coat hanger is evaluated.
Coherent motion detection[111–113]	Vision	Low	2006	A number of dots are moving randomly on a computer screen while a small subset of them coherently move either left or right. The animal has to detect the direction of the coherent motion and respond correctly to it in order to receive a reward.
Cold plate[114–119]	Nociception	Medium	1988*	Cold-induced behavior is assessed by placing the animal on a cold plate. Also see Double plate and Hot plate.
Collins test				See Paw preference testing
Composite neuroscore				See Neurological score

Conditioned place preference[120–132]	Substance dependence	High	1957	The animal is administered a rewarding substance and placed in a test chamber and is then administered vehicle and placed in a second test chamber. The animal is then allowed free access to both chambers to see which one it prefers by comparing the time spent in each chamber. The use of aversive substances or drug withdrawal may cause a conditioned place aversion.
Conditioned odor aversion				See Conditioned taste aversion
Conditioned taste aversion[133–144]	Learning and memory	High	1955	The animal is presented with, and consumes, a novel food/fluid and is thereafter administered a test substance/treatment. If the test substance/treatment has aversive effects the animal may associate the novel food/fluid with the aversive effects and avoid the food/fluid on future presentations if it has an intact learning and memory capacity. The novel food/fluid is usually recognized by its taste but odor can also be used which creates a conditioned odor aversion.
Cork test[145–147]	Catalepsy	Low	1979*	The animal is placed with each paw on top of a cork and the time it retains this position is recorded.
Corner test[23,148–150]	Sensory-motor function, Laterality	Low	2002	The animal approaches and rears into a corner and the direction it turns to following the rear is evaluated. Unilateral impairments in the whisker sensory system may influence turning direction.
Cross maze[151–157]	Visuo-spatial learning and memory, Cognitive flexibility	Low	1974*	A maze shaped like a cross and can for example be used to evaluate spatial learning, procedural learning and to implement rule shifting tasks. The maze can also be filled with water (Water cross maze).
Cylinder test[158–162]	Sensory-motor function, Laterality	Medium	2000	The animal is placed in a transparent cylinder and the rearing behavior is evaluated. Asymmetry in forelimb use during rearing indicates unilateral motor impairments. Aka Spontaneous forelimb use (Fig 2B).
Dalila effect				See Barbering

Delayed alternation[163–165]	Learning and memory, Working memory	Medium	1957*	The animal first performs one of two possible actions, for example turning left or right in a T-maze. After a delay it has to make the alternate choice to receive a reward. Delayed alternation tasks can be performed in mazes (spatial delayed alternation) and operant chambers (operant delayed alternation).
Dig task[33,37]	Learning and memory	Low	2012	The animal is presented with two cups filled with digging media, although only one contains a reward, and therefore has to rely on olfactory cues to identify the correct cup (Fig 2D). Also see Attentional set-shifting, Odor span task and Spatial span task.
Digging for rewards				See Attentional set-shifting, Dig task, Odor span task and Spatial span task.
Distractor sustained attention task				See Sustained attention task
Double plate[118]	Nociception	Low	2006	The time spent on each plate is measure in an arena with one cold plate and one plate at room temperature which the animal can freely move between. Also see Cold plate and Hot plate.
Dowel test				See Balance beam
Drug discrimination[166–185]	Substance dependence	Medium	1951*	The animal is injected with either drug or vehicle and placed in an operant chamber with two levers. One lever results in a food reward only following drug injection and the other only following vehicle injection. Following extensive training, the animal learns the correct response. By then injecting the animal with another drug, its similarity with the first drug can be assessed. The ability of a compound to block the effect of the injected drug can also be evaluated.
Elevated open platform[186–188]	Stress response, Anxiety-like behavior	Low	1979*	The animal is placed on an inescapable elevated open platform to either induce a stress response or to assess anxiety-like behavior.
Elevated plus maze[189–215]	Anxiety-like behavior	High	1985	The time spent in closed (safe) versus open (unsafe) arms, and transitions between them, is used as a measure of anxiety-like behavior. Also see Graded anxiety test and Zero maze.
Elevated T-maze[216–225]	Anxiety-like behavior, Learning and memory	Medium	1993	A T-maze with one enclosed and two open arms is used to assess both anxiety-like behavior as well as memory and learning. Also see Elevated plus maze and T-maze.

Emergence test[226–228]	Anxiety-like behavior	Low	1986*	The time before the animal leaves a safe start box to explore a novel open field is used to evaluate anxiety-like behavior. Also see Light-Dark box and Open field.
Exit circle test[229,230]	Locomotor activity	Low	1988*	The test measures the time needed to exit a 20 to 50 cm diameter circle surrounded by a wall with a single opening. Aka Circle exit test.
Fear conditioning and extinction[231–247]	Learning and memory, Associative learning	High	1941*	The response when exposed to a sensory cue, usually an auditory tone, followed by an aversive event such as an electric foot shock is evaluated. If the animal makes an association between the two events it displays a fear response, typically freezing, when re-exposed to the sensory cue or the context it was delivered in. Repeated exposure to the sensory cue in the absence of the aversive event leads to extinction of the fear response.
Fixed bar test				See Balance beam
Floor projection maze[248]	Vision assessment	Low	2009	Visual information presented on the floor of a maze is used to evaluate visual function.
Food carrying				See Hoarding
Food competition test[249–254]	Social status, Social interactions	Low	1972*	Social status is determined by assessing the animals' behavior when competing for desirable food items.
Forced swim test[255–272]	Depressive-like behavior	High	1977	The time spent trying to escape versus floating in a small water tank with no escape route is measured. A low level of escape behavior is considered to be a depressive-like behavior. Aka Porsolt test.
Forelimb flexion[273–276]	Neurological function, Laterality	Medium	1986*	The ability to extend both forelimbs to break an anticipated fall when suspended by the tail above a surface is evaluated. Neurologically intact animals will extend both forelimbs symmetrically.
Forelimb locomotor assessment scale (FLAS)[277]	Gait	Low	2009	A qualitative scale used to assess gait patterns following cervical spinal cord injury.
Forelimb placing				See Vibrissae-evoked forelimb placing
Formalin test[278–307]	Nociception	High	1977	Pain-evoked behavior following formalin forepaw, orofacial or tail injection is assessed.

Four plate test[308–310]	Anxiety-like behavior	Low	1971	An animal is placed in a cage where the floor consists of four metal plates separated by a small distance and the number of crossing between plates is counted. Every time the animal crosses from one plate to another, it receives a foot shock which creates a conflict between exploratory behavior and avoidance of foot shocks. Also see Geller-Seifter conflict test and Vogel conflict test.
Frey filaments, von				See von Frey filaments
Functional neuroscore				See Neurological score
Gait kinematics[311–319]	Gait	Medium	1993*	The gait pattern when walking or wading is evaluated when the animal is viewed from the side, aided by a high-speed video camera and reflective disks attached to the animal. Optionally, X-ray video recordings may be used to visualize the skeleton.
Gap crossing[320–322]	Sensory function	Low	1986*	The ability to move between two platforms in the dark is used to assess the function of the whisker sensory system.
Geller-Seifter conflict test[202,323–325]	Anxiety-like behavior	Low	1960	The test determines the behavior evoked when lever pressing results in both a food reward and occasionally a mild electric shock. Also see Four plate test and Vogel conflict test.
Graded anxiety test[326]	Anxiety-like behavior	Low	2002	A combination of the Elevated plus maze and Light-dark box with several compartments gradually transitioning from safe to risky. Also see Successive alleys.
Grid performance test				See Inverted grid
Grid walk[95,159,327–330]	Sensory-motor function	Medium	1987*	The number of foot slips made by an animal walking on top of an elevated horizontal grid is recorded. Optionally, the grid can be tilted. Also see Ladder rung walk.
Grip strength[331–336]	Sensory-motor function	High	1978*	The test measures the maximum force exerted by an animal on a bar it holds on to while being pulled backwards by the base of its tail. Can be modified for individual measurement of each forepaw to assess unilateral impairments. Also see Wire grip and Inverted grid.
Grooming evaluation[337–348]	Species-typical behavior	Medium	1960*	The quality and/or amount of grooming are evaluated. Grooming may be evoked by swimming or water application.
Gross neuroscore				See Neurological score

Gustatory neophobia[349–355]	Species-typical behavior, Learning and memory, Anxiety-like behavior	Low	1963*	The test determines to what extent the animal displays the normal rodent behavior of initially avoiding, but eventually accepting, novel types of food. Attenuation of gustatory neophobia during repeated exposure requires intact learning and memory. Gustatory neophobia may also be considered to be an anxiety-like behavior. Aka Hyponeophagia.
Hargreaves test[356–360]	Nociception	High	1988	A beam of light is directed to the animal's hind paw and the withdrawal latency is measured. Aka Plantar test.
Head twitch response[361–366]	Drug induced behavior	Medium	1956	Administration of various hallucinogenic drugs induces rapid side-to-side twitching of the animals head. The response frequency can be evaluated by manual observation, either directly or using video recordings. The head twitch responses can also be automatically assessed by measuring voltage changes caused by the movement of a small magnet attached to the skull of the animal. Also see Locomotor sensitization.
Hoarding[91,367–384]	Species-typical behavior	Low	1939*	The test determines to what extent the animal retrieves food from an external location and hoards it near its nest. Aka Food carrying.
Holeboard[385–396]	Exploratory behavior, Learning and memory	Medium	1962	The extent of exploratory head dips into the holes of an arena with several small holes in the floor is used to assess exploratory behavior. Optionally, certain holes may be baited with food pellets to determine if the animal learns and remembers the location of the baited holes upon repetition.
Horizontal ladder				See Ladder rung walk
Horizontal rope walking[397–399]	Sensory-motor function	Low	2001*	The number of foot slips made when crossing a horizontally suspended rope by walking on top of it is determined. Aka Rope walking.
Hot plate[305,400–411]	Nociception	High	1944	Latency to initiate paw-licking and escape behavior when placed on a hot surface is measured. Also see Cold plate and Double plate.
Hyponeophagia				See Gustatory neophobia
Inclined ladder				See Ladder climb
Inclined plane[276,412,413]	Sensory-motor function, Laterality	Medium	1977	The animal is placed on a horizontal plane which is gradually turned towards the vertical position. The angle the plane is at when the animal loses its grip is recorded. The animal is placed so that either its left or right side is facing down when the plane is inclined. Aka Angle board.
IntelliCage[414–417]	Learning and memory	Medium	2005*	Various tests of learning and memory are carried out in a large cage which serves as both test device and animal housing.

Inverted grid[418–420]	Sensory-motor function	Low	1977	The animal is placed on top of a horizontal grid which is then turned upside down and the animal's ability to hang on to the grid is evaluated. Optionally, the grid is turned to the vertical position. Aka Grid performance test, Screen test and Wire grip. Also see Wire hanging.
Jet ball				See Virtual reality spatial navigation systems
Knee-bend test[421,422]	Nociception	Low	2008*	The animal is gently restrained and the knee joint extended and flexed five times and the amount of squeaks and/or struggle is evaluated. Also see Ankle-bend test.
Ladder climb[423–425]	Sensory-motor function	Low	2006*	The time required for an animal to climb up an inclined ladder is measured. Aka Inclined ladder. Also see Stairway test.
Ladder rung walk[426–430]	Sensory-motor function	Medium	1997*	The number of paw slips, which additionally may be precisely categorized, is counted when the animal walks along a horizontal ladder made of irregularly or regularly spaced rungs. Aka Horizontal ladder. Also see Grid walk.
Lashley III maze[431–434]	Learning and memory	Low	1929	The time required to navigate through a relatively simple maze to reach a reward at the end is measured.
Latent inhibition[435–440]	Sensory information processing	High	1959*	If an animal is repeatedly exposed to a sensory cue which is not paired with a positive or negative reinforcer the animal will consider the sensory cue irrelevant and start to ignore it. If the sensory cue thereafter is paired with a reinforcer it will take a longer time than usual for the animal to make the association between them, latent inhibition, because the sensory cue is initially ignored. Reduced latent inhibition is interpreted as a schizophrenia-like behavior. Also see Prepulse inhibition.
Lateral pulsion[23,273,274,276,441]	Neurological function, Laterality	Medium	1979*	The resistance an animal exerts towards being gently pushed sideways using one hand on each side of the animal is evaluated. Intact animals normally resist the push while unilateral brain insults reduces resistance when pushed towards the contra-lateral side. Aka Bracing test.
Lateralized reaction time task[151,442–445]	Attention, Laterality	Low	1985	To initiate a trial the animal has to perform an extended nose-poke in a centrally located hole. After a short delay a light is turned on to either the left or right side. To receive a reward the animal has to perform another nose-poke at the side indicated by the light.

Ledged tapered beam[446–448]	Sensory-motor function	Low	2002	The test determines the number of foot slips and the time required for an animal to traverse a tapering beam with ledges on either side to reach a goal box. Aka Challenging beam. Also see Beam walk.
Light-dark box[202,449–456]	Anxiety-like behavior	High	1980	Time spent in different chambers and transitions between them is measured in an arena divided into one dark and one brightly illuminated chamber. Also see Emergence test.
Location discrimination[457–459]	Spatial learning and memory	Low	1998*	The animal has to be able to discriminate between two locations and choose the correct one to receive a reward.
Locomotor sensitization[127,185,460–462]	Substance dependence	High	1990*	To what extent repeated drug injections cause an increased spontaneous locomotor activity is assessed. Aka Behavioral sensitization. Also see Head twitch response.
Louisville swim scale[463]	Sensory-motor function	Low	2006	The swimming pattern following spinal cord injury is assessed using a qualitative scale. Also see Swimming test.
Marble burying[464–473]	Obsessive-compulsory like behavior	High	1981*	Glass marbles placed in the cage are buried with bedding material by the rodent. Burying of large amounts of marbles is considered to be an obsessive-compulsory like behavior.
Modified neurological severity score				See Neurological score
Montoya staircase test				See Staircase test
Morris water maze[474–491]	Visuo-spatial learning and memory	High	1981	Upon repeated testing the animal learns the position of a platform hidden under the water surface of a large tank. The latency to swim to the platform is used as a measure of learning and memory. Also see Radial arm water maze.
Multiple T-maze				See T-maze
Multivariate concentric square field[492–494]	Exploratory behavior	Low	2006	Complex behavioral patterns are measured in an arena with several zones of different characteristics and the results are evaluated using multivariate statistics (Fig 2A).
Negative geotaxis[106,495–501]	Vestibular function, Developmental stage, Laterality	Medium	1926	The time required for the animal to turn around (against gravity) to face upwards is measured when placed facing down on an inclined plane. By repeating and noting the number of left and right turns, laterality can be assessed.

Nest building[91,502–505]	Species-typical behavior	Medium	1949*	The animal is given access to nest building material and the quality of the constructed nest is assessed. Aka Bin cotton use.
Neurological score[229,274,276,506–514]	Neurological function	High	1968	A combination of several easily performed tests to evaluate neurological function. The tests included vary widely and several combinations have been used. Aka Bederson scale, Composite neuroscore, Functional neuroscore, Gross neuroscore, Modified neurological severity score, Neurological severity score and Neuroscore.
Neurological severity score				See Neurological score
Neuroscore				See Neurological score
Novel object recognition[515–523]	Learning and memory, Episodic memory	High	1988	Time spent investigating a novel versus a previously encountered object is recorded to determine if an animal remembers the previously encountered object. Aka Spontaneous object recognition. Also see Novel odor recognition.
Novel odor recognition[524,525]	Learning and memory, Episodic memory	Medium	2004*	The amount of time spent exploring a novel odor is used to assess learning and memory. An animal is placed in an arena with a single odor presented at two locations and after a delay, the animal is returned to the arena where a novel odor is presented at one location while the previously encountered odor is presented at the other location. Also see Novel object recognition.
O-maze				See Zero-maze
Oasis maze[526]	Visuo-spatial learning and memory	Low	2005	The time required for a water-deprived animal to locate a source of water in a maze during repeated testing is measured.
Object-location paired associate learning				See Paired associate learning
Odor span task[527–531]	Learning and memory, Working memory, Olfaction	Low	2000	In the first trial the animal digs in a cup filled with scented digging material to retrieve a reward. Two cups are used in the second trial; one cup has the original scent while the other has a novel scent and also contains the reward. As long as the animal correctly chooses the cup with the novel scent an additional cup is introduced in the next trial. Also see Attentional set-shifting, Dig task and Spatial span task.

Olfactometry[532–540]	Olfaction	Low	1958*	Olfactory function can be assessed using an operant conditioning approach. The animal is trained to lick on a metal spout in the presence of an odor, correct response, which results in the delivery of a liquid reward. Licking in the absence of odor, incorrect response, results in a time-out period.
Olfactory discrimination[541–549]	Learning and memory, Olfaction	Medium	1972*	The test determines whether the animal is able to discriminate between odors and use the obtained information to perform the correct choice. Testing can for example be performed using an olfactometer or carried out in the home cage.
Olfactory habituation/dishabituation[87,550,551]	Olfaction	Low	1981	The same odor is repeatedly presented to the animal which gradually loses interest in this odor (habituation). Switch to a novel odor restores interest (dishabituation) if the animal is able to discriminate between the two odors.
Olfactory tubing maze[552–554]	Learning and memory, Olfaction	Low	2002	The test determines the ability of a water-deprived animal to learn and remember how to use olfactory cues to find water in a maze made up of plastic tubing.
One-way active avoidance[555–557]	Learning and memory	Medium	1974*	The test evaluates the ability to learn that a tone in a dark compartment is followed by a foot-shock that can be avoided by escaping to a brightly illuminated compartment. Also see Passive avoidance and Shuttle-box.
Open field[558–569]	Anxiety-like behavior, Locomotor activity	High	1934	The behavioral patterns of animals placed in an open circular or square arena are recorded. Also see Emergence test.
Operant chamber vision assessment[570–572]	Vision assessment	Low	2000*	A grating is displayed on one of several monitors in the operant chamber. If the animal is able to detect the grating it can use this information to perform the correct response and receive a reward.
Operant chamber[39,539,573–581]	Cognitive function, Sensory function	High	1958*	The animal is placed in a small chamber where it can perform nose-pokes, lever presses or touch-screen interactions (responses) to receive a reward. Typically the animal can choose between different responses where only the correct one is rewarded. The number of responses required for a reward can be fixed or progressively increased over a trial. Operant chambers are very versatile and can be used to evaluate several kinds of cognitive and sensory function. Aka Skinner box.
Operant delayed alternation				See Delayed alternation
OptoMotry				See Virtual optomotor system

Paired associate learning[582–589]	Learning and memory, Associative learning	Medium	1993*	The animal has to learn to associate two things to receive a reward. The association can for example be object-location, object-odor or odor-location. The test can be performed in for example operant chambers or in custom designed test equipment.
Partition test[590–592]	Social interaction	Low	1965*	The test determines the amount of investigative behavior evoked when two animals are placed in a cage separated by a perforated divider. Also see Urinary marking patterns.
Passive avoidance[593–612]	Learning and memory	High	1960*	The animal is placed in a test chamber where a certain behavior results in an aversive event (typically an electric shock). After a delay the animal is re-introduced to the test chamber and the avoidance of the behavior which resulted in the aversive event is assessed. The behavior the animal has to avoid can e.g. be to move from a brightly lit to a dark compartment (step-through passive avoidance), stepping down from a platform (step-down passive avoidance) or drinking from a water spout. Also see Active avoidance.
Pasta handling	Skilled forelimb use	Low	1996*	Paw use when handling and eating strands of dry pasta is evaluated. Aka Vermicelli handling test and Capellini handling test.
Paw preference testing[613–620]	Skilled forelimb use, Laterality	Low	1968	The extent of left versus right paw use is assessed when the animal reaches for food through a small opening. A single medial opening (Collins test) or two lateral openings can be used. Aka Collins test.
Paw print analysis[76,621–627]	Gait	Medium	1963*	The gait pattern is assessed by evaluating the paw prints made when an animal, with the paws dyed using two colors of non-toxic paint, is made to run across a sheet of paper. Also see CatWalk.
Pin-prick[421,628,629]	Nociception	Medium	1991*	The animal is lightly pricked with a thin needle and the evoked withdrawal response is assessed.
Plantar test				See Hargreaves test
Play fighting[630–636]	Species-typical behavior	Low	1899	The extent and quality of play fighting between juvenile animals is assessed.
Pole test[637–640]	Sensory-motor function	Medium	1985*	The time required for an animal placed on top of a pole to turn around and descend is measured. Also see Vertical grid.
Porsolt test				See Forced swim test

Postural instability test[162]	Neurological function	Low	2012*	The animal is held by the trunk, head down in an almost vertical position, with its forepaws on a table and the distance the animal has to be moved forward to trigger a step is measured. Also see Stepping test.
Predator odor induced fear[242,641–645]	Anxiety-like behavior	High	1919	The animal's reaction when exposed to odors of predator species such as cats, ferrets and foxes is assessed. Also see Fear conditioning and extinction.
Prehensile traction				See Wire hanging
Prepulse inhibition[646–652]	Sensory information processing	High	1973*	The inhibition of the acoustic startle and tactile startle response by a warning signal (prepulse) is evaluated. Absence of prepulse inhibition is interpreted as a schizophrenia-like behavior. Also see Acoustic startle, Latent inhibition and Tactile startle.
Pull up test[653,654]	Neurological function	Low	1984	The ability of the animal to pull up from a head-down vertical position when held in the hind paws is evaluated.
Radial arm maze[655–664]	Visuo-spatial learning and memory, Working memory	High	1976	The test arena consist of several arms arranged like spokes on a wheel surrounding a central area where all, or some, of the arms are baited with food. The ability of the animal to remember the location of the baited arms between sessions (long term memory) and visited arms during a single session (working memory) is evaluated. Also see Radial arm water maze.
Radial arm water maze[665–669]	Learning and memory	Medium	1985	A hybrid of the Radial arm maze and Morris water maze tests. A Radial arm maze is filled with water and the animal has to find a platform located at the end of one of the arms to escape. Upon repetition the latency to find the platform decreases in animals with an intact learning and memory. Aka Water radial arm maze. Also see Morris water maze and Radial arm maze.
Randall-Selitto test[670–673]	Nociception	Medium	1957	The mechanical pressure required to evoke a withdrawal response is assessed by applying gradually increasing mechanical pressure to the paw. Also see von Frey filaments.
Reaching box				See Tray task
Resident-intruder test[592,674–676]	Social interactions	High	1975*	The behavior evoked by placing an animal (intruder) in the home cage of another animal (resident) is assessed. Typically, the resident will attack the intruder to defend its territory.

Reversal learning[30,581,677–681]	Learning and memory, Cognitive flexibility	High	1957*	The animal first learns that one of two possible responses is rewarded. When performance has reached a criterion the rules are changed and the alternate response is rewarded. Reversal learning can be performed in for example an Operant chamber, the T-maze or Morris water maze. In serial reversal paradigms the correct choice is repeatedly reversed as soon as the criterion is reached.
Rope climbing[427,682–687]	Sensory-motor function	Low	1951*	The ability to climb a vertically suspended rope is evaluated.
Rope walking				See Horizontal rope walking
Rotarod[76,688–700]	Sensory-motor function	High	1957	To avoid falling down, the animal has to run on top of an accelerating, or constant speed, rotating rod. Aka Rotorod.
Rotating pole[701–704]	Sensory-motor function	Low	1995*	The animal has to traverse a horizontal rotating pole and the number of foot slips as well as the time required is measured. Also see Beam walk and Ledged tapered beam.
Rotatory swimming test[705–708]	Lateralization	Low	1990*	The rotational/circling/turning behavior while swimming in a bowl filled with water is assessed by counting the number of clockwise and counter-clockwise laps made around the edge. Also see Rotometer.
Rotometer[620,709–722]	Sensory-motor function, Laterality	Medium	1970*	The animal is placed in a bowl and the rotational/circling/turning behavior is measured by counting the number of clockwise and counter-clockwise circles made around edge of bowl. Aka Bowl test. Also see Rotatory swimming test.
Rotorod				See Rotarod
Round stick balancing[230,723]	Sensory-motor function	Low	2000*	The ability of the animal to perch on a 5 mm diameter stick is evaluated. Also see Wire hanging and Wire traversal.
Running wheel				See Wheel running
Sarter's sustained attention task				See Sustained attention task
Screen test				See Inverted grid
Self-administration[185,724–738]	Substance dependence	High	1962*	The test measures the number of responses, typically lever presses, made to receive a small amount of the substance under investigation via the intra-venous, intra-cranial or oral route. Also see Two-bottle preference test.

Serial implicit learning task[739,740]	Procedural learning	Low	2005	Learning is assessed by determining the degree to which the animal is able to make the correct response to two stimuli in a row in order to receive a reward.
Servo ball				See Virtual reality spatial navigation systems
Set-shifting				See Attentional set-shifting
Shock probe burying[202,741–745]	Anxiety-like behavior, Learning and memory	Medium	1978	The behavior of an animal placed in a cage containing a shock probe is evaluated. Rodents normally bury objects perceived as dangerous and the shock probe is typically promptly covered with cage bedding material immediately following the shock received when investigating the shock probe. Animals re-exposed to the shock probe will bury it before being shocked if they recall the first encounter.
Shuttle-box[556,746–753]	Learning and memory	High	1960*	The animal is placed in a two-compartment arena where a light or sound signal in one of the compartments is followed by an aversive event such as a foot-shock or air puff. The animal must learn to move to the other compartment to avoid the aversive event and the number of aversive events successfully avoided by actively shuttling to the other compartment is recorded. Aka Two-way active avoidance. Also see Passive avoidance.
Single pellet reaching[754–759]	Skilled forelimb use	Medium	1990*	The animal reaches horizontally through a slit for a single pellet at a time. The movement is either described in detail or the amount of dropped pellets counted. Also see Tray task.
Skinner box				See Operant chamber
Social choice				See Social discrimination
Social discrimination[760–763]	Social memory, Social interactions	Medium	1995	The test animal is first exposed to a stimulus animal and after a delay it is exposed to both the first stimulus animal and a second, novel, stimulus animal. If the test animal is able to discriminate between the two stimulus animals the amount of ano-genital investigation directed towards the novel stimulus animal is expected to be higher.
Social habituation/ dishabituation[760,762,764,765]	Social memory, Social interactions	Medium	1995*	The test animal is repeatedly exposed to the same stimulus animal which normally reduces the amount of ano-genital investigations (habituation). On the last trial the test animal is exposed to a novel stimulus animal which normally leads to increased ano-genital investigatory response if the test animal is able to discriminate between the stimulus animals (dishabituation).

Social interaction induced conditioned place preference[766–771]	Social memory, Social interactions	Low	1992	The test evaluates if the animal considerers social interactions to be rewarding by determining if they induce a conditioned place preference.
Social interaction test of anxiety[772–775]	Anxiety-like behavior	Medium	1978	The amount of social interaction is used to evaluate anxiety-like behavior.
Social interaction testing				The amount of investigative behavior towards novel and previously encountered animals can be evaluated using several different protocols. It is typically used to evaluate social memory but can also be used to test anxiety-like behavior. To avoid agonistic behaviors (see Resident-intruder test) and sexual behavior the stimulus animals can be juveniles or ovarectomized females.
Social preference				See Three chamber social approach
Social recognition[760,762,776,777]	Social memory, Social interactions	Medium	1982	The test animal is exposed to a novel stimulus animal and after a delayed it is exposed to the same stimulus animal. If the test animal is able to recognize and remember the stimulus animal the amount of ano-genital investigation typically decrease between trials. As a control experiment two trials with different novel animals can be performed. This typically results in a similar amount of ano-genital investigation in the two trials.
Socially transmitted food preference[778–783]	Learning and memory, Olfaction, Social interactions	Low	1983*	One animal (demonstrator) is allowed to consume a novel food and then allowed to interact with another animal (observer). Guided by olfactory cues in the breath of the demonstrator, the observer animal will then prefer the demonstrated food over other kinds of food if it has an intact learning and memory capacity as well as olfactory ability. By introducing an aversive event in the demonstrator animal, a socially transmitted food aversion may be induced.
Spatial delayed alternation				See Delayed alternation

Spatial span task[151,530]	Learning and memory, Working memory	Low	2000	For the first trial a cup with digging medium, baited with a reward, is placed in one of 24 possible locations. Following each successful trial a cup is added in a novel location and only this cup is baited with a reward. Digging in an un-baited cup is considered a failed trial and the animal has to try again with the same setup. Also see Attentional set-shifting, Dig task and Odor span task.
Spontaneous forelimb use				See Cylinder test
Spontaneous object recognition				See Novel object recognition
Staircase maze[784–787]	Visuo-spatial learning and memory	Low	1970	The animal runs up a wide stair on the left side where only certain steps contain sucrose pellets on right side. Upon repetition, the animal only searches for pellets on correct steps and the time required to find all pellets and the number of errors is used to evaluate learning and memory.
Staircase test[95,758,759,788–794]	Skilled forelimb use	Medium	1991	The amount of successfully retrieved food pellets placed at ledges located at different heights below the animal is recorded. Aka Montoya staircase test.
Stairway test[795]	Sensory-motor function, Laterality	Low	2006	The time required and the extent of left/right deviations made by an animal climbing a broad vertical ladder to reach its home cage is evaluated. Also see Ladder climb.
Step-down passive avoidance				See Passive avoidance
Step-through passive avoidance				See Passive avoidance
Stepping test[441,796,797]	Neurological function	Low	1979*	The test evaluates the stepping pattern evoked when an animal is held above and moved along a horizontal surface with one or both forepaws in contact with it. Also see Postural instability test.
Sticky paper test				See Adhesive removal test
Sticky tape test				See Adhesive removal test
String test				See Wire traversal

Stop signal task[30,38,798,799]	Executive control	Low	2003*	The experimental setup contains a hole for nose-poking and two levers. The animal starts a trial with a nose poke and then presses the first lever. It then has to rapidly press the second lever to receive a reward unless an auditory stop signal is given. In these cases refraining from a lever press is rewarded.
Successive alleys[800,801]	Anxiety-like behavior	Low	2003	The test apparatus consist of four alleys in a row. Each alley is narrower than the previous one and therefore perceived as less safe. The time spent in each alley is used to evaluate anxiety-like behavior. Also see Graded anxiety test.
Sucrose preference test[802–805]	Depressive-like behavior	High	1958*	To what extent the animal prefers a sucrose solution over water is determined, usually using a two-bottle choice procedure. Decreased sensitivity to reward is considered to be a depressive-like behavior similar to anhedonia in patients. Also see Two-bottle preference test.
Sustained attention task[1,806–810]	Attention	Low	1994*	The introduction of two levers into the test chamber can be preceded by a sensory signal or an absence of a signal. The correct response (left or right lever) depends on whether the lever introduction was preceded by a sensory signal or not. The difficulty of the task can be increased by distracting sensory cues (Distractor sustained attention task). Also see 5-choice continuous performance task and 5-choice serial reaction task.
Swimming test[463,811–816]	Sensory-motor function	Low	1970*	The animal is filmed from the side while swimming from one end to the other of a transparent tank. Limb and tail use as well as body posture may be evaluated. Also see Louisville swim scale.
Tactile startle[817–819]	Reflexive behavior	Low	1975*	An unexpected air-puff directed towards the animals back typically causes a startle response which can be automatically measured. Also see Acoustic startle and Pre-pulse inhibition.
Tail suspension test[820–827]	Depressive-like behavior	High	1985	The mouse is suspended by its tail and the time spent immobile versus struggling to escape is measured. Low level of escape behavior is considered to be a depressive-like behavior.

Tail flick[287,305,406,828–834]	Nociception	High	1941	The time to withdrawal of the tail following thermal stimulation using a light beam is measured. Also see Tail immersion.
Tail immersion[835–838]	Nociception	High	1963	The time to withdrawal of the tail following immersion in hot water is measured. Also see Tail flick.
Tarlov scale[839–844]	Gait	Low	1954	A qualitative rating scale used in spinal cord injury research to evaluate gait patterns. Also see Basso, Beattie, Bresnahan (BBB) Locomotor Rating Scale.
Three chamber social approach[592,845–849]	Social interaction	Low	2004	A mouse (test mouse) is introduced into a central empty chamber connected to one chamber with an empty cage and one chamber containing a mouse (target mouse) trapped in a cage. The way the test mouse interacts with the target mouse is evaluated. Optionally, a final phase with two target mice can be performed to evaluate if the test mouse interacts more with the novel target mouse (Fig 2C).
Three-panel runway[850,851]	Visuo-spatial learning and memory	Low	1988	The animal traverses a runway to reach a goal box and at four points along the way it has to choose the correct gate among the three available at each point. The time required to traverse the runway decrease when the test is repeated if the animal learns and remembers the position of the correct gates.
T-maze[163,852–866]	Learning and memory	High	1939*	The ability to choose the right arm of a T-shaped maze is evaluated. The animal can determine the correct choice using sensory cues or by alternating between the left and right arm each trial. Motivation is provided by food deprivation, foot shocks or by filling the maze with water. Optionally, several mazes can be connected (Multiple T-maze) or the maze can be Y-shaped (Y-maze). Access to the maze can optionally be provided by an automated sorting mechanism. Also see Elevated T-maze.
Tongue protusion[789,867,868]	Neurological function	Low	1972*	The ability to use the tongue to obtain food pellets or peanut butter through a small opening is evaluated.
Tray task[869–872]	Skilled forelimb use	Low	1986*	The test determines number of successful horizontal reaches made for food pellets placed on a tray, separated from the animal by vertical metal bars. Optionally, the use of one paw may be restricted using adhesive tape or a bracelet. Aka Reaching box. Also see Single pellet reaching.

Triple test[873,874]	Anxiety-like behavior, Locomotor activity	Low	2008	A combination of Elevated plus maze, Open field and Light-dark box. Designed to avoid the problem of test order effects by running all three tests in a single session.
Tube domination test[592,875–878]	Social status, Social interaction	Low	1961	The behavior of two animals which are introduced at each end of a narrow tube is analyzed. Since they are unable to pass each other, the dominant animal pushes the other backwards.
Two-bottle preference test[879–884]	Substance dependence	High	1940*	Two bottles, commonly one with alcohol and one with water, are placed in the animal's home cage to determine the preferred solution. Also see Self-administration and Sucrose preference test.
Two odor discrimination[885–887]	Learning and memory, Olfaction	Low	1989*	To receive a reward the animal has to nose poke at the correct location, identified by discriminating between two odors. Increased numbers of correct nose pokes over time indicates intact olfactory ability as well as learning and memory capacity.
Two-way active avoidance				See Shuttle-box
Ultrasonic vocalization[16,303,736,888–895]	Species-typical behavior, Developmental stage	Medium	1970*	Pups removed from their nest normally emit ultrasonic vocalizations if they are developing normally. Recordings of ultrasonic vocalizations from adult animals may also aid in the understanding of their behavior by analyzing the type and frequency of vocalizations.
Urinary marking patterns[878,896,897]	Social status, Social interactions	Low	1973	The social status of two animals is evaluated by visualizing urination patterns using ultraviolet light. The animals are placed on each side of a screen in a cage with absorbent material covering the floor. Typically, dominant animals mark the entire area in contrast to subordinate animals that mark only the corners. Also see Partition test.
Vermicelli handling test				See Pasta handling
Vertical grid[420]	Sensory-motor function	Low	2010	The animal is placed on a vertical grid, head up, and the time required to turn around and descend is measured. Also see Pole test.

Vibrissae-evoked forelimb placing[158,161,898–901]	Neurological function, Laterality	Medium	1990*	The motor response evoked by vibrissae touch is evaluated by holding the animal by the torso and moving it either head on or sideways towards the edge of a table or Plexiglas surface. Typically, intact animals place the forelimbs on the table/Plexiglas when the vibrissae touch the side of the table/Plexiglas.
Virtual optomotor system[902–904]	Vision assessment	Medium	2004	The animal is placed on a small platform surrounded by four monitors. When a grating is displayed on the monitors the animal will reflexive move the head to follow the grating if it is able to detect the grating (Fig 2E).
Virtual reality spatial navigation systems[905–910]	Spatial navigation	Low	2005*	An animal with the head fixed or wearing a harness walks on top of a large air-supported Styrofoam ball. As the animal walks on top of the ball it moves around in a virtual reality world displayed on computer monitors in front of it. The system can be combined with operant conditioning techniques, electrophysiological recordings as well as 2-photon imaging.
Visible burrow system[878,911–916]	Species-typical behavior, Social interactions, Social status	Low	1985*	The test assesses social status as well as aggressive and defensive behaviors of a group of animals housed in a semi-natural arena.
Visual cliff avoidance[917–920]	Depth perception, Vision assessment	Low	1957	The animal is placed in an arena with a transparent bottom that extends beyond the edge of the table to create the optical illusion of a cliff. If the animal avoids the cliff or not is determined to assess depth perception ability.
Visual discrimination learning[43,921–927]	Associative learning and memory, Vision assessment	Medium	1954*	The animal is repeatedly presented with two visual stimuli and receives a reward if it is able to discriminate between the images and use this information to perform the correct action. By adjusting the protocol visual discrimination can be used to evaluate learning and memory as well as vision. Visual discrimination learning is typically performed in operant chambers but can also be performed using the Visual water task.
Visual water task[928–930]	Vision assessment	Low	2000	The animal is placed in a water-filled tank with a submerged platform in one of two possible locations. Visual cues guide the animal to the platform if it has sufficient visual acuity to detect them.

Vogel conflict test[202,931–933]	Anxiety-like behavior	Medium	1971	The behavior of water-deprived rodents given access to a water spout which occasionally delivers a mild electric shock is analyzed. The desire to both drink water and to avoid the electric shock creates a conflict situation. Also see Four plate test and Geller-Seifter conflict test.
von Frey filaments[94,360,406,934–944]	Nociception	High	1986*	The pain evoked response following stimulation of the ventral surface of the paw using filaments of different strength is recorded.
Water cross maze				See Cross maze
Water radial arm maze				See Radial arm water maze
Wheel running[945–964]	Locomotor activity, Circadian rhythms	High	1898*	The animal is given free access to a running wheel and its use is analyzed to assess both circadian rhythms and locomotor activity levels. Aka Running wheel.
Whisker nuisance test[965]	Sensory function	Low	2010	The whiskers of a rat are stimulated using a stick. Responses such as withdrawal or stick biting are used to evaluate hyper-sensitivity (Fig 2F).
Whisker trimming				See Barbering
Wire grip				See Grip strength, Inverted grid, Wire hanging and Wire traversal
Wire hanging[966]	Sensory-motor function	Low	2012*	The time the animal is able to hang onto a single horizontal wire with its forepaws is measured. Unlike the Wire traversal test lateral movement of the animal is prevented with screens in this test. Optionally, adhesive tape may be used to prevent hind paw use. Aka Wire grip. Also see Grip strength, Inverted grid and Wire traversal.
Wire traversal[966–968]	Sensory-motor function	Low	1978	The time required for an animal to grip and traverse a single horizontal wire is evaluated. Unlike the Wire hanging test all four paws and tail may be used to traverse the wire if the animal is able to do so. Aka String test and Wire grip. Also see Grip strength, Inverted grid and Wire hanging.
Y-maze				See T-maze
Zero maze[199,213,969–973]	Anxiety-like behavior	Medium	1994	A modification of the Elevated plus maze. By arranging the open and closed arms in a circle, the center zone is excluded. Also see Elevated plus maze.
Ziggurat task[974,975]	Visuo-spatial learning and memory	Low	2008	16 ziggurats (pyramid shaped towers) with a place for a food reward at the top are arranged in a 4x4 grid. The animals have to learn and remember the position of the reward containing ziggurats to obtain the rewards faster.

References

1. Lustig, C., Kozak, R., Sarter, M., Young, J. W. & Robbins, T. W. CNTRICS final animal model task selection: Control of attention. *Neurosci. Biobehav. Rev.* **37,** 2099–110 (2013).

2. Young, J. W., Light, G. A., Marston, H. M., Sharp, R. & Geyer, M. A. The 5-choice continuous performance test: evidence for a translational test of vigilance for mice. *PLoS One* **4,** e4227 (2009).

3. Carli, M., Robbins, T. W., Evenden, J. L. & Everitt, B. J. Effects of lesions to ascending noradrenergic neurones on performance of a 5-choice serial reaction task in rats; implications for theories of dorsal noradrenergic bundle function based on selective attention and arousal. *Behav. Brain Res.* **9,** 361–80 (1983).

4. Robbins, T. W. The 5-choice serial reaction time task: behavioural pharmacology and functional neurochemistry. *Psychopharmacology (Berl).* **163,** 362–80 (2002).

5. Chudasama, Y. & Robbins, T. W. Psychopharmacological approaches to modulating attention in the five-choice serial reaction time task: implications for schizophrenia. *Psychopharmacology (Berl).* **174,** 86–98 (2004).

6. Humby, T., Wilkinson, L. & Dawson, G. Assaying aspects of attention and impulse control in mice using the 5-choice serial reaction time task. *Curr. Protoc. Neurosci.* **Chapter 8,** Unit 8.5H (2005).

7. Higgins, G. A. & Breysse, N. Rodent model of attention: the 5-choice serial reaction time task. *Curr. Protoc. Pharmacol.* **Chapter 5,** Unit5.49 (2008).

8. Bari, A., Dalley, J. W. & Robbins, T. W. The application of the 5-choice serial reaction time task for the assessment of visual attentional processes and impulse control in rats. *Nat. Protoc.* **3,** 759–67 (2008).

9. Robinson, E. S. J. *et al.* Behavioural characterisation of high impulsivity on the 5-choice serial reaction time task: specific deficits in “waiting” versus “stopping”. *Behav. Brain Res.* **196,** 310–6 (2009).

10. Choi, Y., Yoon, Y. W., Na, H. S., Kim, S. H. & Chung, J. M. Behavioral signs of ongoing pain and cold allodynia in a rat model of neuropathic pain. *Pain* **59,** 369–76 (1994).

11. Naruse, K. *et al.* Transplantation of bone marrow-derived mononuclear cells improves mechanical hyperalgesia, cold allodynia and nerve function in diabetic neuropathy. *PLoS One* **6,** e27458 (2011).

12. Landis, C. & Hunt, W. A. *The startle pattern*. (Farrar and Rinehart, 1939).

13. Armus, H. L. & Isham, K. A. Effect of hunger on the acoustic startle reflex. *Psychol. Rep.* **54,** 667–70 (1984).

14. Armus, H. L. & Isham, K. A. Effect of thirst on the acoustic startle reflex. *Psychol. Rep.* **56,** 88–90 (1985).

15. Davis, M. Sensitization of the acoustic startle reflex by footshock. *Behav. Neurosci.* **103,** 495–503 (1989).

16. Kaltwasser, M. T. Acoustic startle induced ultrasonic vocalization in the rat: a novel animal model of anxiety? *Behav. Brain Res.* **43,** 133–7 (1991).

17. Chabot, C. C. & Taylor, D. H. Daily rhythmicity of the rat acoustic startle response. *Physiol. Behav.* **51,** 885–9 (1992).

18. Frankland, P. W. & Ralph, M. R. Circadian modulation in the rat acoustic startle circuit. *Behav. Neurosci.* **109,** 43–8 (1995).

19. Seaman, R. L. & Chen, J. Sensing platform for acoustic startle responses from rat forelimbs and hindlimbs. *IEEE Trans. Biomed. Eng.* **43,** 221–5 (1996).

20. Koch, M. The neurobiology of startle. *Prog. Neurobiol.* **59,** 107–28 (1999).

21. Plappert, C. F. & Pilz, P. K. The acoustic startle response as an effective model for elucidating the effect of genes on the neural mechanism of behavior in mice. *Behav. Brain Res.* **125,** 183–8 (2001).

22. Clause, A., Nguyen, T. & Kandler, K. An acoustic startle-based method of assessing frequency discrimination in mice. *J. Neurosci. Methods* **200,** 63–7 (2011).

23. Schallert, T. *et al.* Tactile extinction: distinguishing between sensorimotor and motor asymmetries in rats with unilateral nigrostriatal damage. *Pharmacol. Biochem. Behav.* **16,** 455–62 (1982).

24. Komotar, R. J. *et al.* Neurologic assessment of somatosensory dysfunction following an experimental rodent model of cerebral ischemia. *Nat. Protoc.* **2,** 2345–7 (2007).

25. Bouet, V. *et al.* The adhesive removal test: a sensitive method to assess sensorimotor deficits in mice. *Nat. Protoc.* **4,** 1560–4 (2009).

26. Freret, T. *et al.* Behavioral deficits after distal focal cerebral ischemia in mice: Usefulness of adhesive removal test. *Behav. Neurosci.* **123,** 224–30 (2009).

27. Calvino, B., Crepon-Bernard, M. O. & Le Bars, D. Parallel clinical and behavioural studies of adjuvant-induced arthritis in the rat: possible relationship with "chronic pain". *Behav. Brain Res.* **24,** 11–29 (1987).

28. Potes, C. S., Neto, F. L. & Castro-Lopes, J. M. Administration of baclofen, a gamma-aminobutyric acid type B agonist in the thalamic ventrobasal complex, attenuates allodynia in monoarthritic rats subjected to the ankle-bend test. *J. Neurosci. Res.* **83,** 515–23 (2006).

29. Pozza, D. H. *et al.* Nociceptive behaviour upon modulation of mu-opioid receptors in the ventrobasal complex of the thalamus of rats. *Pain* **148,** 492–502 (2010).

30. Gilmour, G. *et al.* Measuring the construct of executive control in schizophrenia: Defining and validating translational animal paradigms for discovery research. *Neurosci. Biobehav. Rev.* **37,** 2125–40 (2013).

31. Sauvage, M. M., Beer, Z. & Eichenbaum, H. Recognition memory: adding a response deadline eliminates recollection but spares familiarity. *Learn. Mem.* **17,** 104–8 (2010).

32. Berger-Sweeney, J., Libbey, M., Arters, J., Junagadhwalla, M. & Hohmann, C. F. Neonatal monoaminergic depletion in mice (Mus musculus) improves performance of a novel odor discrimination task. *Behav. Neurosci.* **112,** 1318–26 (1998).

33. Martens, K. M., Vonder Haar, C., Hutsell, B. A. & Hoane, M. R. A discrimination task used as a novel method of testing decision-making behavior following traumatic brain injury. *J. Neurotrauma* **29,** 2505–12 (2012).

34. Wood, E. R., Dudchenko, P. A. & Eichenbaum, H. The global record of memory in hippocampal neuronal activity. *Nature* **397,** 613–6 (1999).

35. Birrell, J. M. & Brown, V. J. Medial frontal cortex mediates perceptual attentional set shifting in the rat. *J. Neurosci.* **20,** 4320–4 (2000).

36. Colacicco, G., Welzl, H., Lipp, H.-P. & Würbel, H. Attentional set-shifting in mice: modification of a rat paradigm, and evidence for strain-dependent variation. *Behav. Brain Res.* **132,** 95–102 (2002).

37. Martens, K. M., Vonder Haar, C., Hutsell, B. A. & Hoane, M. R. The dig task: a simple scent discrimination reveals deficits following frontal brain damage. *J. Vis. Exp.* 1–6 (2013). doi:10.3791/50033

38. Floresco, S. B. & Jentsch, J. D. Pharmacological enhancement of memory and executive functioning in laboratory animals. *Neuropsychopharmacology* **36,** 227–50 (2011).

39. Barrett, J. E. & Vanover, K. E. Assessment of learning and memory using the autoshaping of operant responding in mice. *Curr. Protoc. Neurosci.* **Chapter 8,** Unit 8.5F (2004).

40. Cleland, G. G. & Davey, G. C. Autoshaping in the rat: The effects of localizable visual and auditory signals for food. *J. Exp. Anal. Behav.* **40,** 47–56 (1983).

41. Davenport, J. W. Combined autoshaping-operant (AO) training: CS-UCS interval effects in rats. *Bull. Psychon. Soc.* **3,** 383–385 (1974).

42. Walker, E. A. & Foley, J. J. Acquisition session length modulates consolidation effects produced by 5-HT2C ligands in a mouse autoshaping-operant procedure. *Behav. Pharmacol.* **21,** 83–9 (2010).

43. Horner, A. E. *et al.* The touchscreen operant platform for testing learning and memory in rats and mice. *Nat. Protoc.* **8,** 1961–84 (2013).

44. Modianos, D. T. & Pfaff, D. W. Brain stem and cerebellar lesions in female rats. I. Tests of posture and movement. *Brain Res.* **106,** 31–46 (1976).

45. Hamm, R. J. Neurobehavioral assessment of outcome following traumatic brain injury in rats: an evaluation of selected measures. *J. Neurotrauma* **18,** 1207–16 (2001).

46. Thal, S. C., Mebmer, K., Schmid-Elsaesser, R. & Zausinger, S. Neurological impairment in rats after subarachnoid hemorrhage--a comparison of functional tests. *J. Neurol. Sci.* **268,** 150–9 (2008).

47. Luong, T. N., Carlisle, H. J., Southwell, A. & Patterson, P. H. Assessment of motor balance and coordination in mice using the balance beam. *J. Vis. Exp.* (2011). doi:10.3791/2376

48. Long, S. Y. Hair-nibbling and whisker-trimming as indicators of social hierarchy in mice. *Anim. Behav.* **20,** 10–2 (1972).

49. Strozik, E. & Festing, M. F. Whisker trimming in mice. *Lab. Anim.* **15,** 309–12 (1981).

50. Bresnahan, J. F., Kitchell, B. B. & Wildman, M. F. Facial hair barbering in rats. *Lab. Anim. Sci.* **33,** 290–1 (1983).

51. Sarna, J. R., Dyck, R. H. & Whishaw, I. Q. The Dalila effect: C57BL6 mice barber whiskers by plucking. *Behav. Brain Res.* **108,** 39–45 (2000).

52. Garner, J. P., Weisker, S. M., Dufour, B. & Mench, J. A. Barbering (fur and whisker trimming) by laboratory mice as a model of human trichotillomania and obsessive-compulsive spectrum disorders. *Comp. Med.* **54,** 216–24 (2004).

53. Kalueff, A. V, Minasyan, A., Keisala, T., Shah, Z. H. & Tuohimaa, P. Hair barbering in mice: implications for neurobehavioural research. *Behav. Processes* **71,** 8–15 (2006).

54. Kuschinsky, K. & Hornykiewicz, O. Morphine catalepsy in the rat: relation to striatal dopamine metabolism. *Eur. J. Pharmacol.* **19,** 119–22 (1972).

55. Sanberg, P. R., Pevsner, J. & Coyle, J. T. Parametric influences on catalepsy. *Psychopharmacology (Berl).* **82,** 406–8 (1984).

56. Sanberg, P. R., Bunsey, M. D., Giordano, M. & Norman, A. B. The catalepsy test: its ups and downs. *Behav. Neurosci.* **102,** 748–59 (1988).

57. Ferré, S., Guix, T., Prat, G., Jane, F. & Casas, M. Is experimental catalepsy properly measured? *Pharmacol. Biochem. Behav.* **35,** 753–7 (1990).

58. Alvarez-Cervera, F. J. *et al.* A novel automated rat catalepsy bar test system based on a RISC microcontroller. *J. Neurosci. Methods* **146,** 76–83 (2005).

59. Barnes, C. A. Memory deficits associated with senescence: a neurophysiological and behavioral study in the rat. *J. Comp. Physiol. Psychol.* **93,** 74–104 (1979).

60. Koopmans, G., Blokland, a, Vannieuwenhuijzen, P. & Prickaerts, J. Assessment of spatial learning abilities of mice in a new circular maze. *Physiol. Behav.* **79,** 683–693 (2003).

61. Harrison, F. E., Reiserer, R. S., Tomarken, A. J. & McDonald, M. P. Spatial and nonspatial escape strategies in the Barnes maze. *Learn. Mem.* **13,** 809–19 (2006).

62. O'Leary, T. P., Savoie, V. & Brown, R. E. Learning, memory and search strategies of inbred mouse strains with different visual abilities in the Barnes maze. *Behav. Brain Res.* **216,** 531–42 (2011).

63. O'Leary, T. P. & Brown, R. E. The effects of apparatus design and test procedure on learning and memory performance of C57BL/6J mice on the Barnes maze. *J. Neurosci. Methods* **203,** 315–24 (2012).

64. O'Leary, T. P. & Brown, R. E. Optimization of apparatus design and behavioral measures for the assessment of visuo-spatial learning and memory of mice on the Barnes maze. *Learn. Mem.* **20,** 85–96 (2013).

65. Basso, D. M., Beattie, M. S. & Bresnahan, J. C. A sensitive and reliable locomotor rating scale for open field testing in rats. *J. Neurotrauma* **12,** 1–21 (1995).

66. Scheff, S. W., Saucier, D. A. & Cain, M. E. A statistical method for analyzing rating scale data: the BBB locomotor score. *J. Neurotrauma* **19,** 1251–60 (2002).

67. Ferguson, A. R. *et al.* A simple post hoc transformation that improves the metric properties of the BBB scale for rats with moderate to severe spinal cord injury. *J. Neurotrauma* **21,** 1601–13 (2004).

68. Lebedev, S. V *et al.* Exercise tests and BBB method for evaluation of motor disorders in rats after contusion spinal injury. *Bull. Exp. Biol. Med.* **146,** 489–94 (2008).

69. Wong, J. K., Sharp, K. & Steward, O. A straight alley version of the BBB locomotor scale. *Exp. Neurol.* **217,** 417–20 (2009).

70. Evans, R. M. & Davies, M. ScoreCentre: a computer program to assist with collection and calculation of BBB locomotor scale data. *J. Neurosci. Methods* **194,** 102–7 (2010).

71. Basso, D. M. *et al.* Basso Mouse Scale for locomotion detects differences in recovery after spinal cord injury in five common mouse strains. *J. Neurotrauma* **23,** 635–59 (2006).

72. Shelton, S. B. *et al.* A simple, efficient tool for assessment of mice after unilateral cortex injury. *J. Neurosci. Methods* **168,** 431–42 (2008).

73. Reich, A. *et al.* Fas/CD95 regulatory protein Faim2 is neuroprotective after transient brain ischemia. *J. Neurosci.* **31,** 225–33 (2011).

74. Feeney, D. M., Gonzalez, A. & Law, W. A. Amphetamine, haloperidol, and experience interact to affect rate of recovery after motor cortex injury. *Science* **217,** 855–7 (1982).

75. Drucker-Colín, R. & García-Hernández, F. A new motor test sensitive to aging and dopaminergic function. *J. Neurosci. Methods* **39,** 153–61 (1991).

76. Carter, R. J., Morton, J. & Dunnett, S. B. Motor coordination and balance in rodents. *Curr. Protoc. Neurosci.* **Chapter 8,** Unit 8.12 (2001).

77. Heffernan, M. E. *et al.* Multi-modal approach for investigating brain and behavior changes in an animal model of traumatic brain injury. *J. Neurotrauma* **30,** 1007–12 (2013).

78. Shipley, J. E. *et al.* Increased amphetamine stereotypy and longer haloperidol catalepsy in spontaneously hypertensive rats. *Life Sci.* **28,** 745–53 (1981).

79. Chatterjee, M., Singh, S., Kumari, R., Verma, A. K. & Palit, G. Evaluation of the antipsychotic potential of Panax quinquefolium in ketamine induced experimental psychosis model in mice. *Neurochem. Res.* **37,** 759–70 (2012).

80. Dhanushkodi, A. *et al.* A single intramuscular injection of rAAV-mediated mutant erythropoietin protects against MPTP-induced parkinsonism. *Genes. Brain. Behav.* **12,** 224–33 (2013).

81. Tillerson, J. L. *et al.* Olfactory discrimination deficits in mice lacking the dopamine transporter or the D2 dopamine receptor. *Behav. Brain Res.* **172,** 97–105 (2006).

82. Fleming, S. M. *et al.* Olfactory deficits in mice overexpressing human wildtype alpha-synuclein. *Eur. J. Neurosci.* **28,** 247–56 (2008).

83. Taylor, T. N. *et al.* Nonmotor symptoms of Parkinson's disease revealed in an animal model with reduced monoamine storage capacity. *J. Neurosci.* **29,** 8103–13 (2009).

84. Taylor, T. N., Greene, J. G. & Miller, G. W. Behavioral phenotyping of mouse models of Parkinson's disease. *Behav. Brain Res.* **211,** 1–10 (2010).

85. Alberts, J. R. & Galef, B. G. Acute anosmia in the rat: a behavioral test of a peripherally-induced olfactory deficit. *Physiol. Behav.* **6,** 619–21 (1971).

86. Edwards, D. A., Thompson, M. L. & Burge, K. G. Olfactory bulb removal vs peripherally induced anosmia: differential effects on the aggressive behavior of male mice. *Behav. Biol.* **7,** 823–8 (1972).

87. Yang, M. & Crawley, J. N. Simple behavioral assessment of mouse olfaction. *Curr. Protoc. Neurosci.* **Chapter 8,** Unit 8.24 (2009).

88. Deacon, R. M., Raley, J. M., Perry, V. H. & Rawlins, J. N. Burrowing into prion disease. *Neuroreport* **12,** 2053–7 (2001).

89. Deacon, R. M. J. Burrowing in rodents: a sensitive method for detecting behavioral dysfunction. *Nat. Protoc.* **1,** 118–21 (2006).

90. Deacon, R. M. J. Burrowing: A sensitive behavioural assay, tested in five species of laboratory rodents. *Behav. Brain Res.* **200,** 128–133 (2009).

91. Deacon, R. Assessing burrowing, nest construction, and hoarding in mice. *J. Vis. Exp.* e2607 (2012). doi:10.3791/2607

92. Clarke, K. A. & Still, J. Gait analysis in the mouse. *Physiol. Behav.* **66,** 723–9 (1999).

93. Hamers, F. P., Lankhorst, A. J., van Laar, T. J., Veldhuis, W. B. & Gispen, W. H. Automated quantitative gait analysis during overground locomotion in the rat: its application to spinal cord contusion and transection injuries. *J. Neurotrauma* **18,** 187–201 (2001).

94. Vrinten, D. H. & Hamers, F. F. T. “CatWalk” automated quantitative gait analysis as a novel method to assess mechanical allodynia in the rat; a comparison with von Frey testing. *Pain* **102,** 203–9 (2003).

95. Starkey, M. L. *et al.* Assessing behavioural function following a pyramidotomy lesion of the corticospinal tract in adult mice. *Exp. Neurol.* **195,** 524–39 (2005).

96. Hamers, F. P. T., Koopmans, G. C. & Joosten, E. A. J. CatWalk-assisted gait analysis in the assessment of spinal cord injury. *J. Neurotrauma* **23,** 537–48 (2006).

97. Deumens, R., Jaken, R. J. P., Marcus, M. A. E. & Joosten, E. A. J. The CatWalk gait analysis in assessment of both dynamic and static gait changes after adult rat sciatic nerve resection. *J. Neurosci. Methods* **164,** 120–30 (2007).

98. Gabriel, A. F., Marcus, M. A. E., Honig, W. M. M., Walenkamp, G. H. I. M. & Joosten, E. A. J. The CatWalk method: a detailed analysis of behavioral changes after acute inflammatory pain in the rat. *J. Neurosci. Methods* **163,** 9–16 (2007).

99. Bozkurt, A. *et al.* CatWalk gait analysis in assessment of functional recovery after sciatic nerve injury. *J. Neurosci. Methods* **173,** 91–8 (2008).

100. Angeby-Möller, K., Berge, O.-G. & Hamers, F. P. T. Using the CatWalk method to assess weight-bearing and pain behaviour in walking rats with ankle joint monoarthritis induced by carrageenan: effects of morphine and rofecoxib. *J. Neurosci. Methods* **174,** 1–9 (2008).

101. Gabriel, A. F., Marcus, M. A. E., Walenkamp, G. H. I. M. & Joosten, E. A. J. The CatWalk method: assessment of mechanical allodynia in experimental chronic pain. *Behav. Brain Res.* **198,** 477–80 (2009).

102. Vandeputte, C. *et al.* Automated quantitative gait analysis in animal models of movement disorders. *BMC Neurosci.* **11,** 92 (2010).

103. Boissier, P. J.-R., Tardy, J. & Diverres, J.-C. Une nouvelle méthode simple pour explorer l’action «tranquillisante»: le test de la cheminée. *Pharmacology* **3,** 81–84 (1960).

104. Bouët, V. *et al.* Sensorimotor and cognitive deficits after transient middle cerebral artery occlusion in the mouse. *Exp. Neurol.* **203,** 555–67 (2007).

105. Altman, J. & Sudarshan, K. Postnatal development of locomotion in the laboratory rat. *Anim. Behav.* **23,** 896–920 (1975).

106. Heyser, C. J. Assessment of developmental milestones in rodents. *Curr. Protoc. Neurosci.* **Chapter 8,** Unit 8.18 (2004).

107. Woodworth, K. N., Palmateer, J., Swide, J. & Grafe, M. R. Short- and long-term behavioral effects of exposure to 21%, 40% and 100% oxygen after perinatal hypoxia-ischemia in the rat. *Int. J. Dev. Neurosci.* **29,** 629–38 (2011).

108. Lalonde, R., Qian, S. & Strazielle, C. Transgenic mice expressing the PS1-A246E mutation: effects on spatial learning, exploration, anxiety, and motor coordination. *Behav. Brain Res.* **138,** 71–9 (2003).

109. Morgan, D. *et al.* Apparent behavioral benefits of tau overexpression in P301L tau transgenic mice. *J. Alzheimers. Dis.* **15,** 605–14 (2008).

110. Lalonde, R., Botez, M. I., Joyal, C. C. & Caumartin, M. Motor abnormalities in lurcher mutant mice. *Physiol. Behav.* **51,** 523–5 (1992).

111. Siegel, S. J., Talpos, J. C. & Geyer, M. A. Animal models and measures of perceptual processing in Schizophrenia. *Neurosci. Biobehav. Rev.* **37,** 2092–8 (2013).

112. Douglas, R. M., Neve, a, Quittenbaum, J. P., Alam, N. M. & Prusky, G. T. Perception of visual motion coherence by rats and mice. *Vision Res.* **46,** 2842–7 (2006).

113. Hupfeld, D. & Hoffmann, K.-P. Motion perception in rats (Rattus norvegicus sp.): deficits in albino Wistar rats compared to pigmented Long-Evans rats. *Behav. Brain Res.* **170,** 29–33 (2006).

114. Bennett, G. J. & Xie, Y. K. A peripheral mononeuropathy in rat that produces disorders of pain sensation like those seen in man. *Pain* **33,** 87–107 (1988).

115. Jasmin, L., Kohan, L., Franssen, M., Janni, G. & Goff, J. R. The cold plate as a test of nociceptive behaviors: description and application to the study of chronic neuropathic and inflammatory pain models. *Pain* **75,** 367–82 (1998).

116. Lee, D. E., Kim, S. J. & Zhuo, M. Comparison of behavioral responses to noxious cold and heat in mice. *Brain Res.* **845,** 117–21 (1999).

117. Allchorne, A. J., Broom, D. C. & Woolf, C. J. Detection of cold pain, cold allodynia and cold hyperalgesia in freely behaving rats. *Mol. Pain* **1,** 36 (2005).

118. Walczak, J.-S. & Beaulieu, P. Comparison of three models of neuropathic pain in mice using a new method to assess cold allodynia: the double plate technique. *Neurosci. Lett.* **399,** 240–4 (2006).

119. Tanimoto-Mori, S., Nakazato-Imasato, E., Toide, K. & Kita, Y. Pharmacologic investigation of the mechanism underlying cold allodynia using a new cold plate procedure in rats with chronic constriction injuries. *Behav. Pharmacol.* **19,** 85–90 (2008).

120. Beach, H. D. Morphine addiction in rats. *Can. J. Psychol.* **11,** 104–12 (1957).

121. Hoffman, D. C. The use of place conditioning in studying the neuropharmacology of drug reinforcement. *Brain Res. Bull.* **23,** 373–87 (1989).

122. Schechter, M. D. & Calcagnetti, D. J. Trends in place preference conditioning with a cross-indexed bibliography; 1957-1991. *Neurosci. Biobehav. Rev.* **17,** 21–41 (1993).

123. Perks, S. M. & Clifton, P. G. Reinforcer revaluation and conditioned place preference. *Physiol. Behav.* **61,** 1–5 (1997).

124. Tzschentke, T. M. Measuring reward with the conditioned place preference paradigm: a comprehensive review of drug effects, recent progress and new issues. *Prog. Neurobiol.* **56,** 613–72 (1998).

125. Schechter, M. D. & Calcagnetti, D. J. Continued trends in the conditioned place preference literature from 1992 to 1996, inclusive, with a cross-indexed bibliography. *Neurosci. Biobehav. Rev.* **22,** 827–46 (1998).

126. Bardo, M. T. & Bevins, R. A. Conditioned place preference: what does it add to our preclinical understanding of drug reward? *Psychopharmacology (Berl).* **153,** 31–43 (2000).

127. Tirelli, E., Laviola, G. & Adriani, W. Ontogenesis of behavioral sensitization and conditioned place preference induced by psychostimulants in laboratory rodents. *Neurosci. Biobehav. Rev.* **27,** 163–178 (2003).

128. Carboni, E. & Vacca, C. Conditioned place preference. A simple method for investigating reinforcing properties in laboratory animals. *Methods Mol. Med.* **79,** 481–98 (2003).

129. Roux, S., Froger, C., Porsolt, R. D., Valverde, O. & Maldonado, R. Place preference test in rodents. *Curr. Protoc. Neurosci.* **Chapter 9,** Unit 9.15 (2003).

130. Cunningham, C. L., Gremel, C. M. & Groblewski, P. A. Drug-induced conditioned place preference and aversion in mice. *Nat. Protoc.* **1,** 1662–70 (2006).

131. Prus, A. J., James, J. R. & Rosecrans, J. A. in *Methods Behav. Anal. Neurosci.* (Buccafusco, J.) (CRC Press, 2009).

132. Myers, K. M., Bechtholt-Gompf, A. J., Coleman, B. R. & Carlezon, W. A. Extinction of conditioned opiate withdrawal in rats in a two-chambered place conditioning apparatus. *Nat. Protoc.* **7,** 517–26 (2012).

133. Garcia, J., Kimeldorf, D. J. & Koelling, R. A. Conditioned aversion to saccharin resulting from exposure to gamma radiation. *Science* **122,** 157–8 (1955).

134. Hankins, W. G., Garcia, J. & Rusiniak, K. W. Dissociation of odor and taste in baitshyness. *Behav. Biol.* **8,** 407–19 (1973).

135. Deutsch, J. A., Davis, J. K. & Cap, M. Conditioned taste aversion: oral and postingestional factors. *Behav. Biol.* **18,** 545–50 (1976).

136. Palmerino, C. C., Rusiniak, K. W. & Garcia, J. Flavor-illness aversions: the peculiar roles of odor and taste in memory for poison. *Science* **208,** 753–5 (1980).

137. Panhuber, H. Effect of odor quality and intensity on conditioned odor aversion learning in the rat. *Physiol. Behav.* **28,** 149–54 (1982).

138. Klosterhalfen, S. & Klosterhalfen, W. Conditioned taste aversion and traditional learning. *Psychol. Res.* **47,** 71–94 (1985).

139. Frank, M. E. Sensory physiology of taste and smell discriminations using conditioned food aversion methodology. *Ann. N. Y. Acad. Sci.* **443,** 89–99 (1985).

140. Ervin, G. N. & Cooper, B. R. Use of conditioned taste aversion as a conflict model: effects of anxiolytic drugs. *J. Pharmacol. Exp. Ther.* **245,** 137–46 (1988).

141. Riley, A. L. & Freeman, K. B. Conditioned taste aversion: a database. *Pharmacol. Biochem. Behav.* **77,** 655–6 (2004).

142. Davis, C. M. & Riley, A. L. Conditioned taste aversion learning: implications for animal models of drug abuse. *Ann. N. Y. Acad. Sci.* **1187,** 247–75 (2010).

143. Quintero, E. *et al.* Effects of context novelty vs. familiarity on latent inhibition with a conditioned taste aversion procedure. *Behav. Processes* **86,** 242–9 (2011).

144. Tovar-Díaz, J., González-Sánchez, H. & Roldán-Roldán, G. Association of stimuli at long intervals in conditioned odor aversion. *Physiol. Behav.* **103,** 144–7 (2011).

145. Worms, P. & Lloyd, K. G. Predictability and specificity of behavioral screening tests for neuroleptics. *Pharmacol. Ther. B.* **5,** 445–50 (1979).

146. De Ryck, M., Schallert, T. & Teitelbaum, P. Morphine versus haloperidol catalepsy in the rat: a behavioral analysis of postural support mechanisms. *Brain Res.* **201,** 143–72 (1980).

147. Wardas, J., Konieczny, J. & Pietraszek, M. Influence of CGS 21680, a selective adenosine A(2A) agonist, on the phencyclidine-induced sensorimotor gating deficit and motor behaviour in rats. *Psychopharmacology (Berl).* **168,** 299–306 (2003).

148. Schallert, T., Upchurch, M., Wilcox, R. E. & Vaughn, D. M. Posture-independent sensorimotor analysis of inter-hemispheric receptor asymmetries in neostriatum. *Pharmacol. Biochem. Behav.* **18,** 753–9 (1983).

149. Zhang, L. *et al.* A test for detecting long-term sensorimotor dysfunction in the mouse after focal cerebral ischemia. *J. Neurosci. Methods* **117,** 207–14 (2002).

150. Liesz, A. *et al.* Inhibition of lymphocyte trafficking shields the brain against deleterious neuroinflammation after stroke. *Brain* **134,** 704–720 (2011).

151. Young, J. W., Powell, S. B., Risbrough, V., Marston, H. M. & Geyer, M. A. Using the MATRICS to guide development of a preclinical cognitive test battery for research in schizophrenia. *Pharmacol. Ther.* **122,** 150–202 (2009).

152. Floresco, S. B., Magyar, O., Ghods-Sharifi, S., Vexelman, C. & Tse, M. T. L. Multiple dopamine receptor subtypes in the medial prefrontal cortex of the rat regulate set-shifting. *Neuropsychopharmacology* **31,** 297–309 (2006).

153. Thomas, J. B. Cross-maze avoidance behavior and septal lesions in the rat. *Physiol. Behav.* **14,** 163–7 (1974).

154. Salimov, R. M., McBride, W. J., McKinzie, D. L., Lumeng, L. & Li, T. K. Effects of ethanol consumption by adolescent alcohol-preferring P rats on subsequent behavioral performance in the cross-maze and slip funnel tests. *Alcohol* **13,** 297–300 (1996).

155. Cressant, A. & Granon, S. Definition of a new maze paradigm for the study of spatial behavior in rats. *Brain Res. Protoc.* **12,** 116–124 (2003).

156. Vohora, D., Pal, S. N. & Pillai, K. K. Modulation of spontaneous alternation performance of mice treated with thioperamide and tacrine in a cross maze task. *Fundam. Clin. Pharmacol.* **19,** 531–2 (2005).

157. Kleinknecht, K. R. *et al.* Hippocampus-dependent place learning enables spatial flexibility in C57BL6/N mice. *Front. Behav. Neurosci.* **6,** 87 (2012).

158. Schallert, T., Fleming, S. M., Leasure, J. L., Tillerson, J. L. & Bland, S. T. CNS plasticity and assessment of forelimb sensorimotor outcome in unilateral rat models of stroke, cortical ablation, parkinsonism and spinal cord injury. *Neuropharmacology* **39,** 777–87 (2000).

159. Baskin, Y. K., Dietrich, W. D. & Green, E. J. Two effective behavioral tasks for evaluating sensorimotor dysfunction following traumatic brain injury in mice. *J. Neurosci. Methods* **129,** 87–93 (2003).

160. Gharbawie, O. A., Whishaw, P. A. & Whishaw, I. Q. The topography of three-dimensional exploration: a new quantification of vertical and horizontal exploration, postural support, and exploratory bouts in the cylinder test. *Behav. Brain Res.* **151,** 125–35 (2004).

161. Woodlee, M. T. *et al.* Testing forelimb placing "across the midline" reveals distinct, lesion-dependent patterns of recovery in rats. *Exp. Neurol.* **191,** 310–7 (2005).

162. Khaing, Z. Z. *et al.* Assessing forelimb function after unilateral cervical spinal cord injury: novel forelimb tasks predict lesion severity and recovery. *J. Neurotrauma* **29,** 488–98 (2012).

163. Schaefers, A. T. U. & Winter, Y. Rapid task acquisition of spatial-delayed alternation in an automated T-maze by mice. *Behav. Brain Res.* **225,** 56–62 (2011).

164. Rossi, M. A. *et al.* Prefrontal cortical mechanisms underlying delayed alternation in mice. *J. Neurophysiol.* **108,** 1211–22 (2012).

165. Petrinovich, L. & Bolles, R. Delayed alternation: evidence for symbolic processes in the rat. *J. Comp. Physiol. Psychol.* **50,** 363–5 (1957).

166. Conger, J. J. The effects of alcohol on conflict behavior in the albino rat. *Q. J. Stud. Alcohol* **12,** 1–29 (1951).

167. Colpaert, F. C., Niemegeers, C. J. & Janssen, P. A. Theoretical and methodological considerations on drug discrimination learning. *Psychopharmacologia* **46,** 169–77 (1976).

168. Overton, D. A. Drug discrimination training with progressively lowered doses. *Science* **205,** 720–1 (1979).

169. Overton, D. A. Influence of shaping procedures and schedules of reinforcement on performance in the two-bar drug discrimination task: A methodological report. *Psychopharmacology (Berl).* **65,** 291–298 (1979).

170. Overton, D. A. Multiple drug training as a method for increasing the specificity of the drug discrimination procedure. *J. Pharmacol. Exp. Ther.* **221,** 166–72 (1982).

171. Colpaert, F. C. & Janssen, P. A. OR discrimination: a new drug discrimination method. *Eur. J. Pharmacol.* **78,** 141–4 (1982).

172. Schechter, M. D. Drug sensitivity of individual rats determines degree of drug discrimination. *Pharmacol. Biochem. Behav.* **19,** 1–4 (1983).

173. Overton, D. A. & Hayes, M. W. Optimal training parameters in the two-bar fixed-ratio drug discrimination task. *Pharmacol. Biochem. Behav.* **21,** 19–28 (1984).

174. Koek, W. & Slangen, J. L. External stimulus control in a "drug-discrimination" procedure: drug effects and inter-animal variation. *Psychopharmacology (Berl).* **82,** 168–73 (1984).

175. Holtzman, S. G. Drug discrimination studies. *Drug Alcohol Depend.* **14,** 263–82 (1985).

176. Tomie, A., Loukas, E., Stafford, I., Peoples, L. & Wagner, G. C. Drug discrimination training with a single choice trial per session. *Psychopharmacology (Berl).* **86,** 217–22 (1985).

177. Stolerman, I. P. & Shine, P. J. Trends in drug discrimination research analysed with a cross-indexed bibliography, 1982-1983. *Psychopharmacology (Berl).* **86,** 1–11 (1985).

178. Harris, C. M., Wood, D. M., Lal, H. & Emmett-Oglesby, M. W. A method to shorten the training phase of drug discrimination. *Psychopharmacology (Berl).* **93,** 435–6 (1987).

179. Tomie, A., Peoples, L. & Wagner, G. C. Effects of single or multiple choice trials per session on drug discrimination performance. *Psychopharmacology (Berl).* **92,** 529–35 (1987).

180. Schechter, M. D. Advantages and disadvantages of a rapid method to train drug discrimination. *Pharmacol. Biochem. Behav.* **31,** 239–42 (1988).

181. Meisch, R. A. Establishment of drug discrimination and drug reinforcement in different animal strains: some methodological issues. *J. Addict. Dis.* **10,** 179–87 (1991).

182. Colombo, G. *et al.* T-maze and food reinforcement: an inexpensive drug discrimination procedure. *J. Neurosci. Methods* **67,** 83–7 (1996).

183. Appel, J. B., West, W. B., Rolandi, W. G., Alici, T. & Pechersky, K. Increasing the selectivity of drug discrimination procedures. *Pharmacol. Biochem. Behav.* **64,** 353–8 (1999).

184. Solinas, M., Panlilio, L. V, Justinova, Z., Yasar, S. & Goldberg, S. R. Using drug-discrimination techniques to study the abuse-related effects of psychoactive drugs in rats. *Nat. Protoc.* **1,** 1194–206 (2006).

185. Paterson, N. E. Assessment of substance abuse liability in rodents: self-administration, drug discrimination, and locomotor sensitization. *Curr. Protoc. Pharmacol.* **Chapter 5,** Unit5.62 (2012).

186. Balfour, D. J. & Reid, A. Effects of betamethasone on the stimulation of corticosterone secretion in rats. *Arch. Int. Pharmacodyn. Ther.* **237,** 67–74 (1979).

187. Storey, J. D. *et al.* Behavioural and neurochemical responses evoked by repeated exposure to an elevated open platform. *Behav. Brain Res.* **166,** 220–9 (2006).

188. Miyata, S. *et al.* Effects of serotonergic anxiolytics on the freezing behavior in the elevated open-platform test in mice. *J. Pharmacol. Sci.* **105,** 272–8 (2007).

189. File, S. E. & Pellow, S. The effects of triazolobenzodiazepines in two animal tests of anxiety and in the holeboard. *Br. J. Pharmacol.* **86,** 729–35 (1985).

190. Pellow, S., Chopin, P., File, S. E. & Briley, M. Validation of open:closed arm entries in an elevated plus-maze as a measure of anxiety in the rat. *J. Neurosci. Methods* **14,** 149–67 (1985).

191. Andrews, N. & File, S. E. Handling history of rats modifies behavioural effects of drugs in the elevated plus-maze test of anxiety. *Eur. J. Pharmacol.* **235,** 109–12 (1993).

192. File, S. E. & Zangrossi, H. "One-trial tolerance" to the anxiolytic actions of benzodiazepines in the elevated plus-maze, or the development of a phobic state? *Psychopharmacology (Berl).* **110,** 240–4 (1993).

193. File, S. E. The interplay of learning and anxiety in the elevated plus-maze. *Behav. Brain Res.* **58,** 199–202 (1993).

194. Hogg, S. A review of the validity and variability of the elevated plus-maze as an animal model of anxiety. *Pharmacol. Biochem. Behav.* **54,** 21–30 (1996).

195. Becker, A. & Grecksch, G. Illumination has no effect on rats' behavior in the elevated plus-maze. *Physiol. Behav.* **59,** 1175–7 (1996).

196. Rodgers, R. J. & Dalvi, A. Anxiety, defence and the elevated plus-maze. *Neurosci. Biobehav. Rev.* **21,** 801–10 (1997).

197. Espejo, E. F. Effects of weekly or daily exposure to the elevated plus-maze in male mice. *Behav. Brain Res.* **87,** 233–8 (1997).

198. Fernández Espejo, E. Structure of the mouse behaviour on the elevated plus-maze test of anxiety. *Behav. Brain Res.* **86,** 105–12 (1997).

199. Weiss, S. M., Wadsworth, G., Fletcher, A. & Dourish, C. T. Utility of ethological analysis to overcome locomotor confounds in elevated maze models of anxiety. *Neurosci. Biobehav. Rev.* **23,** 265–71 (1998).

200. Bertoglio, L. J. & Carobrez, A. P. Previous maze experience required to increase open arms avoidance in rats submitted to the elevated plus-maze model of anxiety. *Behav. Brain Res.* **108,** 197–203 (2000).

201. Martínez, J. C., Cardenas, F., Lamprea, M. & Morato, S. The role of vision and proprioception in the aversion of rats to the open arms of an elevated plus-maze. *Behav. Processes* **60,** 15–26 (2002).

202. File, S. E., Lippa, A. S., Beer, B. & Lippa, M. T. Animal tests of anxiety. *Curr. Protoc. Neurosci.* **Chapter 8,** Unit 8.3 (2004).

203. Mulder, G. B. & Pritchett, K. The elevated plus-maze. *Contemp. Top. Lab. Anim. Sci.* **43,** 39–40 (2004).

204. Carobrez, A. P. & Bertoglio, L. J. Ethological and temporal analyses of anxiety-like behavior: the elevated plus-maze model 20 years on. *Neurosci. Biobehav. Rev.* **29,** 1193–205 (2005).

205. Patel, P. D., Seasholtz, A. F. & Patel, P. D. Computer-assisted scoring of the elevated plus maze. *Biotechniques* **41,** 700, 702, 704 (2006).

206. Clénet, F., Bouyon, E., Hascoët, M. & Bourin, M. Light/dark cycle manipulation influences mice behaviour in the elevated plus maze. *Behav. Brain Res.* **166,** 140–9 (2006).

207. Walf, A. A. & Frye, C. A. The use of the elevated plus maze as an assay of anxiety-related behavior in rodents. *Nat. Protoc.* **2,** 322–8 (2007).

208. Botelho, S., Estanislau, C. & Morato, S. Effects of under- and overcrowding on exploratory behavior in the elevated plus-maze. *Behav. Processes* **74,** 357–62 (2007).

209. Komada, M., Takao, K. & Miyakawa, T. Elevated plus maze for mice. *J. Vis. Exp.* 1–4 (2008).

210. Violle, N., Balandras, F., Le Roux, Y., Desor, D. & Schroeder, H. Variations in illumination, closed wall transparency and/or extramaze space influence both baseline anxiety and response to diazepam in the rat elevated plus-maze. *Behav. Brain Res.* **203,** 35–42 (2009).

211. Sidor, M. M., Rilett, K. & Foster, J. A. Validation of an automated system for measuring anxiety-related behaviours in the elevated plus maze. *J. Neurosci. Methods* **188,** 7–13 (2010).

212. Schneider, P., Ho, Y.-J., Spanagel, R. & Pawlak, C. R. A novel elevated plus-maze procedure to avoid the one-trial tolerance problem. *Front. Behav. Neurosci.* **5,** 43 (2011).

213. Braun, A. A., Skelton, M. R., Vorhees, C. V & Williams, M. T. Comparison of the elevated plus and elevated zero mazes in treated and untreated male Sprague-Dawley rats: effects of anxiolytic and anxiogenic agents. *Pharmacol. Biochem. Behav.* **97,** 406–15 (2011).

214. Belviranli, M., Atalik, K. E. N., Okudan, N. & Gökbel, H. Age and sex affect spatial and emotional behaviors in rats: the role of repeated elevated plus maze test. *Neuroscience* **227,** 1–9 (2012).

215. Casarrubea, M. *et al.* Temporal structure of the rat's behavior in elevated plus maze test. *Behav. Brain Res.* **237,** 290–9 (2013).

216. Graeff, F. G., Viana, M. B. & Tomaz, C. The elevated T maze, a new experimental model of anxiety and memory: effect of diazepam. *Braz. J. Med. Biol. Res.* **26,** 67–70 (1993).

217. Viana, M. B., Tomaz, C. & Graeff, F. G. The elevated T-maze: a new animal model of anxiety and memory. *Pharmacol. Biochem. Behav.* **49,** 549–54 (1994).

218. Zangrossi, H. & Graeff, F. G. Behavioral validation of the elevated T-maze, a new animal model of anxiety. *Brain Res. Bull.* **44,** 1–5 (1997).

219. Graeff, F. G., Netto, C. F. & Zangrossi, H. The elevated T-maze as an experimental model of anxiety. *Neurosci. Biobehav. Rev.* **23,** 237–46 (1998).

220. Jardim, M. C., Nogueira, R. L., Graeff, F. G. & Nunes-de-Souza, R. L. Evaluation of the elevated T-maze as an animal model of anxiety in the mouse. *Brain Res. Bull.* **48,** 407–11 (1999).

221. Conde, C. A., Costa, V. & Tomaz, C. Measuring emotional memory in the elevated T-maze using a training-to-criterion procedure. *Pharmacol. Biochem. Behav.* **63,** 63–9 (1999).

222. De-Mello, N. & Carobrez, A. P. Elevated T-maze as an animal model of memory: effects of scopolamine. *Behav. Pharmacol.* **13,** 139–48 (2002).

223. Carvalho-Netto, E. . & Nunes-de-Souza, R. . Use of the elevated T-maze to study anxiety in mice. *Behav. Brain Res.* **148,** 119–132 (2004).

224. Torrejais, J. C. M., Rosa, C. C. M., Boerngen-Lacerda, R. & Andreatini, R. The elevated T-maze as a measure of two types of defensive reactions: a factor analysis. *Brain Res. Bull.* **76,** 376–9 (2008).

225. Asth, L., Lobão-Soares, B., André, E., Soares, V. D. P. & Gavioli, E. C. The elevated T-maze task as an animal model to simultaneously investigate the effects of drugs on long-term memory and anxiety in mice. *Brain Res. Bull.* **87,** 526–33 (2012).

226. Holson, R. R. Mesial prefrontal cortical lesions and timidity in rats. I. Reactivity to aversive stimuli. *Physiol. Behav.* **37,** 221–30 (1986).

227. Lalonde, R. & Strazielle, C. The relation between open-field and emergence tests in a hyperactive mouse model. *Neuropharmacology* **57,** 722–4 (2009).

228. Leibrock, C. *et al.* Akt2 Deficiency is Associated with Anxiety and Depressive Behavior in Mice. *Cell. Physiol. Biochem.* **32,** 766–77 (2013).

229. Shapira, Y., Davidson, E., Weidenfeld, Y., Cotev, S. & Shohami, E. Dexamethasone and indomethacin do not affect brain edema following head injury in rats. *J. Cereb. Blood Flow Metab.* **8,** 395–402 (1988).

230. Flierl, M. A. *et al.* Mouse closed head injury model induced by a weight-drop device. *Nat. Protoc.* **4,** 1328–37 (2009).

231. Estes, W. K. & Skinner, B. F. Some quantitative properties of anxiety. *J. Exp. Psychol.* **29,** 390–400 (1941).

232. Valentinuzzi, V. S. *et al.* Automated measurement of mouse freezing behavior and its use for quantitative trait locus analysis of contextual fear conditioning in (BALB/cJ x C57BL/6J)F2 mice. *Learn. Mem.* **5,** 391–403 (1998).

233. Otto, T., Cousens, G. & Herzog, C. Behavioral and neuropsychological foundations of olfactory fear conditioning. *Behav. Brain Res.* **110,** 119–28 (2000).

234. Anagnostaras, S. G. Computer-Assisted Behavioral Assessment of Pavlovian Fear Conditioning in Mice. *Learn. Mem.* **7,** 58–72 (2000).

235. Watson, J. B. & Rayner, R. Conditioned emotional reactions. 1920. *Am. Psychol.* **55,** 313–7 (2000).

236. Maren, S. Neurobiology of Pavlovian fear conditioning. *Annu. Rev. Neurosci.* **24,** 897–931 (2001).

237. Contarino, A., Baca, L., Kennelly, A. & Gold, L. H. Automated assessment of conditioning parameters for context and cued fear in mice. *Learn. Mem.* **9,** 89–96 (2002).

238. Rudy, J. W., Huff, N. C. & Matus-Amat, P. Understanding contextual fear conditioning: insights from a two-process model. *Neurosci. Biobehav. Rev.* **28,** 675–85 (2004).

239. Baldi, E., Lorenzini, C. A. & Bucherelli, C. Footshock intensity and generalization in contextual and auditory-cued fear conditioning in the rat. *Neurobiol. Learn. Mem.* **81,** 162–6 (2004).

240. Wehner, J. M. & Radcliffe, R. A. Cued and contextual fear conditioning in mice. *Curr. Protoc. Neurosci.* **Chapter 8,** Unit 8.5C (2004).

241. Kopec, C. D. *et al.* A robust automated method to analyze rodent motion during fear conditioning. *Neuropharmacology* **52,** 228–33 (2007).

242. Takahashi, L. K., Chan, M. M. & Pilar, M. L. Predator odor fear conditioning: current perspectives and new directions. *Neurosci. Biobehav. Rev.* **32,** 1218–27 (2008).

243. Maren, S. Pavlovian fear conditioning as a behavioral assay for hippocampus and amygdala function: cautions and caveats. *Eur. J. Neurosci.* **28,** 1661–6 (2008).

244. Chang, C. *et al.* Fear extinction in rodents. *Curr. Protoc. Neurosci.* **Chapter 8,** Unit8.23 (2009).

245. Curzon, P., Rustay, N. & Browman, K. in *Methods Behav. Anal. Neurosci.* (Buccafusco, J.) (CRC Press, 2009). at <http://www.ncbi.nlm.nih.gov/books/NBK5223/>

246. Burman, M. A., Murawski, N. J., Schiffino, F. L., Rosen, J. B. & Stanton, M. E. Factors governing single-trial contextual fear conditioning in the weanling rat. *Behav. Neurosci.* **123,** 1148–52 (2009).

247. Meuth, P. *et al.* Standardizing the analysis of conditioned fear in rodents: a multidimensional software approach. *Genes. Brain. Behav.* **12,** 583–92 (2013).

248. Furtak, S. C. *et al.* The Floor Projection Maze: A novel behavioral apparatus for presenting visual stimuli to rats. *J. Neurosci. Methods* **181,** 82–8 (2009).

249. Manosevitz, M. Behavioral heterosis: food competition in mice. *J. Comp. Physiol. Psychol.* **79,** 46–50 (1972).

250. Gellert, V. F. & Sparber, S. B. Effects of morphine withdrawal on food competition hierarchies and fighting behavior in rats. *Psychopharmacology (Berl).* **60,** 165–72 (1979).

251. Gentsch, C., Lichtsteiner, M. & Feer, H. Competition for sucrose-pellets in triads of male Wistar rats: the individuals' performances are differing but stable. *Behav. Brain Res.* **27,** 37–44 (1988).

252. Whishaw, I. Q. Food wrenching and dodging: use of action patterns for the analysis of sensorimotor and social behavior in the rat. *J. Neurosci. Methods* **24,** 169–78 (1988).

253. Merlot, E., Moze, E., Bartolomucci, A., Dantzer, R. & Neveu, P. J. The rank assessed in a food competition test influences subsequent reactivity to immune and social challenges in mice. *Brain. Behav. Immun.* **18,** 468–75 (2004).

254. Millard, A. & Gentsch, C. Competition for sucrose pellets in tetrads of male Wistar, Fischer or Sprague-Dawley rats: is intra-group ranking reflected in the level of anxiety? *Behav. Brain Res.* **168,** 243–54 (2006).

255. Porsolt, R. D., Bertin, A. & Jalfre, M. Behavioral despair in mice: a primary screening test for antidepressants. *Arch. Int. Pharmacodyn. Ther.* **229,** 327–36 (1977).

256. Shimamura, M., Kobayashi, T., Kuratani, K. & Kinoshita, M. Optimized analysis of the forced swim test using an automated experimental system: detailed time course study in mice. *J. Pharmacol. Toxicol. Methods* **57,** 80–4 (1977).

257. Porsolt, R. D., Le Pichon, M. & Jalfre, M. Depression: a new animal model sensitive to antidepressant treatments. *Nature* **266,** 730–2 (1977).

258. Nishimura, H., Tsuda, A., Oguchi, M., Ida, Y. & Tanaka, M. Is immobility of rats in the forced swim test "behavioral despair"? *Physiol. Behav.* **42,** 93–5 (1988).

259. West, A. P. Neurobehavioral studies of forced swimming: the role of learning and memory in the forced swim test. *Prog. Neuropsychopharmacol. Biol. Psychiatry* **14,** 863–77 (1990).

260. Abel, E. L. Physiological correlates of the forced swim test in rats. *Physiol. Behav.* **54,** 309–17 (1993).

261. Abel, E. L. Behavioral and physiological effects of different water depths in the forced swim test. *Physiol. Behav.* **56,** 411–4 (1994).

262. Abel, E. L. A further analysis of physiological changes in rats in the forced swim test. *Physiol. Behav.* **56,** 795–800 (1994).

263. Hédou, G., Pryce, C., Di Iorio, L., Heidbreder, C. a & Feldon, J. An automated analysis of rat behavior in the forced swim test. *Pharmacol. Biochem. Behav.* **70,** 65–76 (2001).

264. Kurtuncu, M., Luka, L. J., Dimitrijevic, N., Uz, T. & Manev, H. Reliability assessment of an automated forced swim test device using two mouse strains. *J. Neurosci. Methods* **149,** 26–30 (2005).

265. Petit-Demouliere, B., Chenu, F. & Bourin, M. Forced swimming test in mice: a review of antidepressant activity. *Psychopharmacology (Berl).* **177,** 245–55 (2005).

266. Shimamura, M., Kuratani, K. & Kinoshita, M. A new automated and high-throughput system for analysis of the forced swim test in mice based on magnetic field changes. *J. Pharmacol. Toxicol. Methods* **55,** 332–6 (2007).

267. Gersner, R., Gordon-Kiwkowitz, M. & Zangen, A. Automated behavioral analysis of limbs' activity in the forced swim test. *J. Neurosci. Methods* **180,** 82–6 (2009).

268. Kulikov, A. V, Morozova, M. V, Kulikov, V. A., Kirichuk, V. S. & Popova, N. K. Automated analysis of antidepressants' effect in the forced swim test. *J. Neurosci. Methods* **191,** 26–31 (2010).

269. Mezadri, T. J., Batista, G. M., Portes, A. C., Marino-Neto, J. & Lino-de-Oliveira, C. Repeated rat-forced swim test: reducing the number of animals to evaluate gradual effects of antidepressants. *J. Neurosci. Methods* **195,** 200–5 (2011).

270. Hayashi, E., Shimamura, M., Kuratani, K., Kinoshita, M. & Hara, H. Automated experimental system capturing three behavioral components during murine forced swim test. *Life Sci.* **88,** 411–7 (2011).

271. Can, A. *et al.* The mouse forced swim test. *J. Vis. Exp.* e3638 (2012). doi:10.3791/3638

272. Slattery, D. A. & Cryan, J. F. Using the rat forced swim test to assess antidepressant-like activity in rodents. *Nat. Protoc.* **7,** 1009–14 (2012).

273. Bederson, J. B. *et al.* Rat middle cerebral artery occlusion: evaluation of the model and development of a neurologic examination. *Stroke.* **17,** 472–6 (1986).

274. McIntosh, T. K., Noble, L., Andrews, B. & Faden, A. I. Traumatic brain injury in the rat: characterization of a midline fluid-percussion model. *Cent. Nerv. Syst. Trauma* **4,** 119–34 (1987).

275. Rodriguez, R., Santiago-Mejia, J., Gomez, C. & San-Juan, E. R. A simplified procedure for the quantitative measurement of neurological deficits after forebrain ischemia in mice. *J. Neurosci. Methods* **147,** 22–8 (2005).

276. Kabadi, S. V, Hilton, G. D., Stoica, B. A., Zapple, D. N. & Faden, A. I. Fluid-percussion-induced traumatic brain injury model in rats. *Nat. Protoc.* **5,** 1552–63 (2010).

277. Anderson, K. D. *et al.* Forelimb locomotor assessment scale (FLAS): novel assessment of forelimb dysfunction after cervical spinal cord injury. *Exp. Neurol.* **220,** 23–33 (2009).

278. Dubuisson, D. & Dennis, S. G. The formalin test: a quantitative study of the analgesic effects of morphine, meperidine, and brain stem stimulation in rats and cats. *Pain* **4,** 161–74 (1977).

279. Wahlberg, J. E. & Kartus, E. Stability of formalin test solutions. *Contact Dermatitis* **7,** 43–4 (1981).

280. Hunskaar, S., Fasmer, O. B. & Hole, K. Formalin test in mice, a useful technique for evaluating mild analgesics. *J. Neurosci. Methods* **14,** 69–76 (1985).

281. Murray, C. W., Porreca, F. & Cowan, A. Methodological refinements to the mouse paw formalin test. An animal model of tonic pain. *J. Pharmacol. Methods* **20,** 175–86 (1988).

282. Clavelou, P., Pajot, J., Dallel, R. & Raboisson, P. Application of the formalin test to the study of orofacial pain in the rat. *Neurosci. Lett.* **103,** 349–53 (1989).

283. Rosland, J. H., Tjølsen, A., Maehle, B. & Hole, K. The formalin test in mice: effect of formalin concentration. *Pain* **42,** 235–42 (1990).

284. Rosland, J. H. The formalin test in mice: the influence of ambient temperature. *Pain* **45,** 211–6 (1991).

285. Wheeler-Aceto, H. & Cowan, A. Standardization of the rat paw formalin test for the evaluation of analgesics. *Psychopharmacology (Berl).* **104,** 35–44 (1991).

286. Tjølsen, A., Berge, O. G., Hunskaar, S., Rosland, J. H. & Hole, K. The formalin test: an evaluation of the method. *Pain* **51,** 5–17 (1992).

287. Hole, K. & Tjølsen, A. The tail-flick and formalin tests in rodents: changes in skin temperature as a confounding factor. *Pain* **53,** 247–54 (1993).

288. Coderre, T. J., Fundytus, M. E., McKenna, J. E., Dalal, S. & Melzack, R. The formalin test: a validation of the weighted-scores method of behavioural pain rating. *Pain* **54,** 43–50 (1993).

289. Abbott, F. V, Franklin, K. B. & Westbrook, R. F. The formalin test: scoring properties of the first and second phases of the pain response in rats. *Pain* **60,** 91–102 (1995).

290. Clavelou, P., Dallel, R., Orliaguet, T., Woda, a & Raboisson, P. The orofacial formalin test in rats: effects of different formalin concentrations. *Pain* **62,** 295–301 (1995).

291. Jett, M. F. & Michelson, S. The formalin test in rat: validation of an automated system. *Pain* **64,** 19–25 (1996).

292. Jourdan, D. *et al.* A new automated method of pain scoring in the formalin test in rats. *Pain* **71,** 265–70 (1997).

293. Watson, G. S., Sufka, K. J. & Coderre, T. J. Optimal scoring strategies and weights for the formalin test in rats. *Pain* **70,** 53–8 (1997).

294. Sufka, K., Watson, G., Nothdurft, R. & Mogil, J. Scoring the mouse formalin test: validation study. *Eur. J. Pain* **2,** 351–358 (1998).

295. Teng, C. J. & Abbott, F. V. The formalin test: a dose-response analysis at three developmental stages. *Pain* **76,** 337–47 (1998).

296. Abbott, F. V, Ocvirk, R., Najafee, R. & Franklin, K. B. Improving the efficiency of the formalin test. *Pain* **83,** 561–9 (1999).

297. Jourdan, D., Alloui, a & Eschalier, a. Pharmacological validation of an automated method of pain scoring in the formalin test in rats. *J. Pharmacol. Toxicol. Methods* **42,** 163–70 (1999).

298. Saddi, G. & Abbott, F. V. The formalin test in the mouse: a parametric analysis of scoring properties. *Pain* **89,** 53–63 (2000).

299. Kolesnikov, Y., Cristea, M., Oksman, G., Torosjan, A. & Wilson, R. Evaluation of the tail formalin test in mice as a new model to assess local analgesic effects. *Brain Res.* **1029,** 217–23 (2004).

300. Raboisson, P. & Dallel, R. The orofacial formalin test. Neurosci. Biobehav. Rev. 28, 219–26 (2004).

301. Capone, F. & Aloisi, A. M. Refinement of pain evaluation techniques. The formalin test. *Ann. Ist. Super. Sanita* **40,** 223–9 (2004).

302. Xie, Y., Wang, J., Huo, F., Jia, H. & Tang, J. Validation of a simple automated movement detection system for formalin test in rats. *Acta Pharmacol. Sin.* **26,** 39–45 (2005).

303. Oliveira, A. R. & Barros, H. M. T. Ultrasonic rat vocalizations during the formalin test: a measure of the affective dimension of pain? *Anesth. Analg.* **102,** 832–9 (2006).

304. Luccarini, P., Childeric, A., Gaydier, A.-M., Voisin, D. & Dallel, R. The orofacial formalin test in the mouse: a behavioral model for studying physiology and modulation of trigeminal nociception. *J. Pain* **7,** 908–14 (2006).

305. Bannon, A. W. & Malmberg, A. B. Models of nociception: hot-plate, tail-flick, and formalin tests in rodents. *Curr. Protoc. Neurosci.* **Chapter 8,** Unit 8.9 (2007).

306. Bornhof, M., Ihmsen, H., Schwilden, H., Yeomans, D. C. & Tzabazis, A. The orofacial formalin test in mice revisited--effects of formalin concentration, age, morphine and analysis method. *J. Pain* **12,** 633–9 (2011).

307. Grégoire, S. *et al.* New method to discriminate sedative and analgesic effects of drugs in the automated formalin test in rats. *Neurosci. Res.* **72,** 194–8 (2012).

308. Aron, C., Simon, P., Larousse, C. & Boissier, J. R. Evaluation of a rapid technique for detecting minor tranquilizers. *Neuropharmacology* **10,** 459–69 (1971).

309. Hascoet, M., Bourin, M. & Couetoux du Tertre, a. Influence of prior experience on mice behavior using the four-plate test. *Pharmacol. Biochem. Behav.* **58,** 1131–8 (1997).

310. Bourin, M. & Hascoët, M. Implication of 5-HT2 receptor subtypes in the mechanism of action of the GABAergic compound etifoxine in the four-plate test in Swiss mice. *Behav. Brain Res.* **208,** 352–8 (2010).

311. Molinari, M. & Petrosini, L. Hemicerebellectomy and motor behaviour in rats. III. Kinematics of recovered spontaneous locomotion after lesions at different developmental stages. *Behav. Brain Res.* **54,** 43–55 (1993).

312. Jamon, M. & Clarac, F. Early walking in the neonatal rat: a kinematic study. *Behav. Neurosci.* **112,** 1218–28 (1998).

313. Metz, G. A., Merkler, D., Dietz, V., Schwab, M. E. & Fouad, K. Efficient testing of motor function in spinal cord injured rats. *Brain Res.* **883,** 165–77 (2000).

314. Muir, G. D. & Whishaw, I. Q. Red nucleus lesions impair overground locomotion in rats: a kinetic analysis. *Eur. J. Neurosci.* **12,** 1113–22 (2000).

315. Bolton, D. A. E., Tse, A. D. Y., Ballermann, M., Misiaszek, J. E. & Fouad, K. Task specific adaptations in rat locomotion: runway versus horizontal ladder. *Behav. Brain Res.* **168,** 272–9 (2006).

316. Garnier, C., Falempin, M. & Canu, M.-H. A 3D analysis of fore- and hindlimb motion during locomotion: comparison of overground and ladder walking in rats. *Behav. Brain Res.* **186,** 57–65 (2008).

317. Zörner, B. *et al.* Profiling locomotor recovery: comprehensive quantification of impairments after CNS damage in rodents. *Nat. Methods* **7,** 701–708 (2010).

318. Gravel, P., Tremblay, M., Leblond, H., Rossignol, S. & de Guise, J. A. A semi-automated software tool to study treadmill locomotion in the rat: from experiment videos to statistical gait analysis. *J. Neurosci. Methods* **190,** 279–88 (2010).

319. Bauman, J. M. & Chang, Y.-H. High-speed X-ray video demonstrates significant skin movement errors with standard optical kinematics during rat locomotion. *J. Neurosci. Methods* **186,** 18–24 (2010).

320. Hutson, K. A. & Masterton, R. B. The sensory contribution of a single vibrissa's cortical barrel. *J. Neurophysiol.* **56,** 1196–223 (1986).

321. Jenkinson, E. W. & Glickstein, M. Whiskers, barrels, and cortical efferent pathways in gap crossing by rats. *J. Neurophysiol.* **84,** 1781–9 (2000).

322. Papaioannou, S., Brigham, L. & Krieger, P. Sensory deprivation during early development causes an increased exploratory behavior in a whisker-dependent decision task. *Brain Behav.* **3,** 24–34 (2013).

323. Geller, I. & Seifter, J. The effects of meprobamate, barbiturates, d-amphetamine and promazine on experimentally induced conflict in the rat. *Psychopharmacologia* **1,** 482–492 (1960).

324. Geller, I., Kulak, J. T. & Seifter, J. The effects of chlordiazepoxide and chlorpromazine on a punishment discrimination. *Psychopharmacologia* **3,** 374–85 (1962).

325. Paterson, N. E. & Hanania, T. The modified Geller-Seifter test in rats was insensitive to GABAB receptor positive modulation or blockade, or 5-HT1A receptor activation. *Behav. Brain Res.* **208,** 258–64 (2010).

326. Dere, E. *et al.* The graded anxiety test: a novel test of murine unconditioned anxiety based on the principles of the elevated plus-maze and light-dark test. *J. Neurosci. Methods* **122,** 65–73 (2002).

327. Bresnahan, J. C., Beattie, M. S., Todd, F. D. & Noyes, D. H. A behavioral and anatomical analysis of spinal cord injury produced by a feedback-controlled impaction device. *Exp. Neurol.* **95,** 548–70 (1987).

328. Somerson, S. K. & Stokes, B. T. Functional analysis of an electromechanical spinal cord injury device. *Exp. Neurol.* **96,** 82–96 (1987).

329. Prakriya, M., McCabe, P. M. & Holets, V. R. A computerized grid walking system for evaluating the accuracy of locomotion in rats. *J. Neurosci. Methods* **48,** 15–25 (1993).

330. Chao, O. Y., Pum, M. E., Li, J.-S. & Huston, J. P. The grid-walking test: assessment of sensorimotor deficits after moderate or severe dopamine depletion by 6-hydroxydopamine lesions in the dorsal striatum and medial forebrain bundle. *Neuroscience* **202,** 318–25 (2012).

331. Cabe, P. A., Tilson, H. A., Mitchell, C. L. & Dennis, R. A simple recording grip strength device. *Pharmacol. Biochem. Behav.* **8,** 101–2 (1978).

332. Meyer, O. A., Tilson, H. A., Byrd, W. C. & Riley, M. T. A method for the routine assessment of fore- and hindlimb grip strength of rats and mice. *Neurobehav. Toxicol.* **1,** 233–6 (1979).

333. Dunnett, S. B., Torres, E. M. & Annett, L. E. A lateralised grip strength test to evaluate unilateral nigrostriatal lesions in rats. *Neurosci. Lett.* **246,** 1–4 (1998).

334. Maurissen, J. P. ., Marable, B. R., Andrus, A. K. & Stebbins, K. E. Factors affecting grip strength testing. *Neurotoxicol. Teratol.* **25,** 543–553 (2003).

335. Crabbe, J. C. *et al.* Strain differences in three measures of ethanol intoxication in mice: the screen, dowel and grip strength tests. *Genes. Brain. Behav.* **2,** 201–13 (2003).

336. Yousuf, S., Atif, F., Sayeed, I., Wang, J. & Stein, D. G. Post-stroke infections exacerbate ischemic brain injury in middle-aged rats: immunomodulation and neuroprotection by progesterone. *Neuroscience* **239,** 92–102 (2013).

337. Bolles, R. C. Grooming behavior in the rat. *J. Comp. Physiol. Psychol.* **53,** 306–10 (1960).

338. Hopkins, D. A. The sequential organization of grooming behaviour in the rat. *Brain Res.* **24,** 550 (1970).

339. Annable, A. & Wearden, J. H. Grooming movements as operants in the rat. *J. Exp. Anal. Behav.* **32,** 297–304 (1979).

340. Spruijt, B. M., Welbergen, P., Brakkee, J. & Gispen, W. H. An ethological analysis of excessive grooming in young and aged rats. *Ann. N. Y. Acad. Sci.* **525,** 89–100 (1988).

341. Young, R. K. & Thiessen, D. D. Washing, drying, and anointing in adult humans (Homo sapiens): commonalities with grooming sequences in rodents. *J. Comp. Psychol.* **105,** 340–4 (1991).

342. Berridge, K. C. & Whishaw, I. Q. Cortex, striatum and cerebellum: control of serial order in a grooming sequence. *Exp. Brain Res.* **90,** 275–90 (1992).

343. Bertelli, J. A. & Mira, J. C. Behavioral evaluating methods in the objective clinical assessment of motor function after experimental brachial plexus reconstruction in the rat. *J. Neurosci. Methods* **46,** 203–8 (1993).

344. Kalueff, A. V & Tuohimaa, P. Grooming analysis algorithm for neurobehavioural stress research. *Brain Res. Brain Res. Protoc.* **13,** 151–8 (2004).

345. Kalueff, A. V & Tuohimaa, P. The grooming analysis algorithm discriminates between different levels of anxiety in rats: potential utility for neurobehavioural stress research. *J. Neurosci. Methods* **143,** 169–77 (2005).

346. Chen, S.-K. *et al.* Hematopoietic origin of pathological grooming in Hoxb8 mutant mice. *Cell* **141,** 775–85 (2010).

347. Kyzar, E. *et al.* Towards high-throughput phenotyping of complex patterned behaviors in rodents: focus on mouse self-grooming and its sequencing. *Behav. Brain Res.* **225,** 426–31 (2011).

348. Pires, G. N., Tufik, S. & Andersen, M. L. Grooming analysis algorithm: use in the relationship between sleep deprivation and anxiety-like behavior. *Prog. Neuropsychopharmacol. Biol. Psychiatry* **41,** 6–10 (2013).

349. Barnet, S. *The rat: A study in behavior*. (Aldline, 1963).

350. Rolls, E. T. & Rolls, B. J. Altered food preferences after lesions in the basolateral region of the amygdala in the rat. *J. Comp. Physiol. Psychol.* **83,** 248–59 (1973).

351. Hamm, R. J. *et al.* Impaired gustatory neophobia following traumatic brain injury in rats. *J. Neurotrauma* **12,** 307–14 (1995).

352. Figueroa-Guzmán, Y., Kuo, J. S. & Reilly, S. NMDA receptor antagonist MK-801 infused into the insular cortex prevents the attenuation of gustatory neophobia in rats. *Brain Res.* **1114,** 183–6 (2006).

353. Figueroa-Guzmán, Y. & Reilly, S. NMDA receptors in the basolateral amygdala and gustatory neophobia. *Brain Res.* **1210,** 200–3 (2008).

354. Deacon, R. M. J. Hyponeophagia: a measure of anxiety in the mouse. *J. Vis. Exp.* 2–5 (2011).

355. Chaperon, F. *et al.* Gastrin-releasing peptide signaling plays a limited and subtle role in amygdala physiology and aversive memory. *PLoS One* **7,** e34963 (2012).

356. Hargreaves, K., Dubner, R., Brown, F., Flores, C. & Joris, J. A new and sensitive method for measuring thermal nociception in cutaneous hyperalgesia. *Pain* **32,** 77–88 (1988).

357. Murphy, L. G., Alexander, G. M. & Schwartzman, R. J. Improvement to the Hargreaves paw flick method. *Pain* **46,** 347 (1991).

358. Ortiz, M. I. *et al.* Evaluation of the interaction between acemetacin and opioids on the hargreaves model of thermal hyperalgesia. *Pharmacol. Biochem. Behav.* **88,** 47–54 (2007).

359. Kocevski, D. & Tvrdeić, A. The effect of repeated daily measurements on paw withdrawal latencies in Hargreaves test. *Coll. Antropol.* **32 Suppl 1,** 93–7 (2008).

360. Austin, P. J., Wu, A. & Moalem-Taylor, G. Chronic constriction of the sciatic nerve and pain hypersensitivity testing in rats. *J. Vis. Exp.* (2012).

361. Keller, D. L. & Umbreit, W. W. Permanent alteration of behavior in mice by chemical and psychological means. *Science* **124,** 723–4 (1956).

362. Corne, S. J., Pickering, R. W. & Warner, B. T. A method for assessing the effects of drugs on the central actions of 5-hydroxytryptamine. *Br. J. Pharmacol. Chemother.* **20,** 106–20 (1963).

363. Singleton, C. & Marsden, C. A. Circadian variation in the head twitch response produced by 5-methoxy-N1,N1-dimethyltryptamine and p-chloroamphetamine in the mouse. *Psychopharmacology (Berl).* **74,** 173–6 (1981).

364. Eble, J. P. & Goodrich, C. The maturational onset of the 5-HT mediated head twitch in mice. *Physiol. Behav.* **41,** 47–51 (1987).

365. Canal, C. E. & Morgan, D. Head-twitch response in rodents induced by the hallucinogen 2,5-dimethoxy-4-iodoamphetamine: a comprehensive history, a re-evaluation of mechanisms, and its utility as a model. *Drug Test. Anal.* **4,** 556–76 (2012).

366. Halberstadt, A. L. & Geyer, M. A. Characterization of the head-twitch response induced by hallucinogens in mice: detection of the behavior based on the dynamics of head movement. *Psychopharmacology (Berl).* **227,** 727–39 (2013).

367. Hunt, J. M. & Willoughby, R. R. The effect of frustration on hoarding in rats. *Psychosom. Med.* **1,** 309–10 (1939).

368. Miller, G. A. & Postman, L. Individual and group hoarding in rats. *Am. J. Psychol.* **59,** 652–68 (1946).

369. Marx, M. H. Experimental analysis of the hoarding habit in the rat; preliminary observations. *J. Comp. Physiol. Psychol.* **43,** 295–308 (1950).

370. Ross, S., Smith, W. I. & Denenberg, V. A preliminary study of individual and group hoarding in the white rat. *J. Genet. Psychol.* **77,** 123–7 (1950).

371. Denenberg, V. H. Hoarding in the white rat under isolation and group conditions. *J. Comp. Physiol. Psychol.* **45,** 497–503 (1952).

372. Smith, W. & Ross, S. The hoarding behavior of the mouse. III. The storing of non-relevant material. *J. Genet. Psychol.* **82,** 309–16 (1953).

373. Stamm, J. S. Hoarding and aggressive behavior in rats. *J. Comp. Physiol. Psychol.* **48,** 324–6 (1955).

374. Marx, M. H., Iwahara, S. & Brownstein, A. J. Hoarding behavior in the hooded rat as a function of varied alley illumination. *J. Genet. Psychol.* **90,** 213–8 (1957).

375. Lowney, E. D. Characteristics of food-carrying behavior in the rat. *J. Comp. Physiol. Psychol.* **51,** 565–9 (1958).

376. Lanier, D. L., Estep, D. Q. & Dewsbury, D. A. Food hoarding in muroid rodents. *Behav. Biol.* **11,** 177–87 (1974).

377. Beigneux, F., Lassalle, J. M. & Le Pape, G. Hoarding behavior of AKR, C57BL/6 mice and their F1 in their home living space: an automatic recording technique. *Physiol. Behav.* **24,** 1191–3 (1980).

378. Borker, A. S., Dhume, R. A. & Gogate, M. G. Measure of motivational drive for hoarding in laboratory rats. *Indian J. Physiol. Pharmacol.* **29,** 126–8 (1985).

379. Cole, M. R. Operant hoarding: a new paradigm for the study of self-control. *J. Exp. Anal. Behav.* **53,** 247–61 (1990).

380. Whishaw, I. Q., Coles, B. L. & Bellerive, C. H. Food carrying: a new method for naturalistic studies of spontaneous and forced alternation. *J. Neurosci. Methods* **61,** 139–43 (1995).

381. Gibson, B. M. & Shettleworth, S. J. Competition among spatial cues in a naturalistic food-carrying task. *Learn. Behav.* **31,** 143–59 (2003).

382. Charron, I. & Cabanac, M. Influence of pellet size on rat's hoarding behavior. *Physiol. Behav.* **82,** 447–51 (2004).

383. Onuki, Y., Kato, K. & Makino, J. Influences of food type on food-carrying behavior in rats (Rattus norvegicus). *Behav. Processes* **70,** 182–5 (2005).

384. Deacon, R. M. J. Assessing hoarding in mice. *Nat. Protoc.* **1,** 2828–30 (2006).

385. Boissier, J. R. & Simon, P. La reaction d'exploration chez la souris. *Therapie* **17,** 1225–32 (1962).

386. File, S. E. & Wardill, A. G. Validity of head-dipping as a measure of exploration in a modified hole-board. *Psychopharmacologia* **44,** 53–9 (1975).

387. File, S. E. & Wardill, A. G. The reliability of the hole-board apparatus. *Psychopharmacologia* **44,** 47–51 (1975).

388. Oades, R. D. & Isaacson, R. L. The development of food search behavior by rats: the effects of hippocampal damage and haloperidol. *Behav. Biol.* **24,** 327–37 (1978).

389. Iversen, I. H. & Mogensen, J. A multipurpose vertical holeboard with automated recording of spatial and temporal response patterns for rodents. *J. Neurosci. Methods* **25,** 251–63 (1988).

390. Durcan, M. J. & Lister, R. G. Does directed exploration influence locomotor activity in a holeboard test? *Behav. Neural Biol.* **51,** 121–5 (1989).

391. File, S. E. & Fluck, E. Handling alters habituation and response to stimulus change in the holeboard. *Pharmacol. Biochem. Behav.* **49,** 449–53 (1994).

392. Brosnan-Watters, G. & Wozniak, D. F. A rotating holeboard procedure for testing drug effects on spatial learning and memory in mice. *Brain Res. Brain Res. Protoc.* **1,** 331–8 (1997).

393. Heim, C. *et al.* The analysis system COGITAT for the study of cognitive deficiencies in rodents. *Behav. Res. Methods. Instrum. Comput.* **32,** 140–56 (2000).

394. Kuc, K. A., Gregersen, B. M., Gannon, K. S. & Dodart, J.-C. Holeboard discrimination learning in mice. *Genes. Brain. Behav.* **5,** 355–63 (2006).

395. Post, A. M. *et al.* The COGITAT holeboard system as a valuable tool to assess learning, memory and activity in mice. *Behav. Brain Res.* **220,** 152–8 (2011).

396. Van der Staay, F. J., Gieling, E. T., Pinzón, N. E., Nordquist, R. E. & Ohl, F. The appetitively motivated "cognitive" holeboard: a family of complex spatial discrimination tasks for assessing learning and memory. *Neurosci. Biobehav. Rev.* **36,** 379–403 (2012).

397. Kim, D. *et al.* Transplantation of Genetically Modified Fibroblasts Expressing BDNF in Adult Rats with a Subtotal Hemisection Improves Specific Motor and Sensory Functions. *Neurorehabil. Neural Repair* **15,** 141–150 (2001).

398. Anderson, K. D., Gunawan, A. & Steward, O. Quantitative assessment of forelimb motor function after cervical spinal cord injury in rats: relationship to the corticospinal tract. *Exp. Neurol.* **194,** 161–74 (2005).

399. Houle, J. D. *et al.* Combining an autologous peripheral nervous system "bridge" and matrix modification by chondroitinase allows robust, functional regeneration beyond a hemisection lesion of the adult rat spinal cord. *J. Neurosci.* **26,** 7405–15 (2006).

400. Woolfe, G. & Macdonald, A. D. The evaluation of the analgesic action of pethidine hydrochloride (demerol). *J. Pharmacol .Exp. Ther.* 300–307 (1944).

401. Ohlsson, L. Algesimetric studies by the hot plate method in mice. *Acta Pharmacol. Toxicol. (Copenh).* **9,** 322–31 (1953).

402. Hunskaar, S., Berge, O. G. & Hole, K. A modified hot-plate test sensitive to mild analgesics. *Behav. Brain Res.* **21,** 101–8 (1986).

403. Espejo, E. F. & Mir, D. Structure of the rat's behaviour in the hot plate test. *Behav. Brain Res.* **56,** 171–6 (1993).

404. Espejo, E. F. & Mir, D. Differential effects of weekly and daily exposure to the hot plate on the rat's behavior. *Physiol. Behav.* **55,** 1157–62 (1994).

405. Bannon, A. W. Models of pain: hot-plate and formalin test in rodents. *Curr. Protoc. Pharmacol.* **Chapter 5,** Unit 5.7 (2001).

406. Mulder, G. B. & Pritchett, K. Rodent analgesiometry: the hot plate, tail flick and Von Frey hairs. *Contemp. Top. Lab. Anim. Sci.* **43,** 54–5 (2004).

407. Yezierski, R. P. & Vierck, C. J. Should the hot-plate test be reincarnated? *J. Pain* **12,** 936–7; author reply 938–9 (2011).

408. Casarrubea, M., Sorbera, F., Santangelo, A. & Crescimanno, G. Learning influence on the behavioral structure of rat response to pain in hot-plate. *Behav. Brain Res.* **225,** 177–83 (2011).

409. Gunn, A., Bobeck, E. N., Weber, C. & Morgan, M. M. The influence of non-nociceptive factors on hot-plate latency in rats. *J. Pain* **12,** 222–7 (2011).

410. Neelakantan, H. & Walker, E. A. Temperature-dependent enhancement of the antinociceptive effects of opioids in combination with gabapentin in mice. *Eur. J. Pharmacol.* **686,** 55–9 (2012).

411. Casarrubea, M., Sorbera, F., Santangelo, A. & Crescimanno, G. The effects of diazepam on the behavioral structure of the rat's response to pain in the hot-plate test: anxiolysis vs. pain modulation. *Neuropharmacology* **63,** 310–21 (2012).

412. Rivlin, A. S. & Tator, C. H. Objective clinical assessment of motor function after experimental spinal cord injury in the rat. *J. Neurosurg.* **47,** 577–81 (1977).

413. Chang, M.-W., Young, M.-S. & Lin, M.-T. An inclined plane system with microcontroller to determine limb motor function of laboratory animals. *J. Neurosci. Methods* **168,** 186–94 (2008).

414. Endo, T. *et al.* Automated test of behavioral flexibility in mice using a behavioral sequencing task in IntelliCage. *Behav. Brain Res.* **221,** 172–81 (2011).

415. Codita, A. *et al.* Impaired behavior of female tg-ArcSwe APP mice in the IntelliCage: A longitudinal study. *Behav. Brain Res.* **215,** 83–94 (2010).

416. Krackow, S. *et al.* Consistent behavioral phenotype differences between inbred mouse strains in the IntelliCage. *Genes. Brain. Behav.* **9,** 722–31 (2010).

417. Galsworthy, M. J. *et al.* A comparison of wild-caught wood mice and bank voles in the Intellicage: assessing exploration, daily activity patterns and place learning paradigms. *Behav. Brain Res.* **157,** 211–7 (2005).

418. Coughenour, L. L., Mclean, J. R. & Parker, R. B. A new device for the rapid measurement of impaired motor function in mice. *Pharmacol. Biochem. Behav.* **6,** 351–3 (1977).

419. Tillerson, J. L. & Miller, G. W. Grid performance test to measure behavioral impairment in the MPTP-treated-mouse model of parkinsonism. *J. Neurosci. Methods* **123,** 189–200 (2003).

420. Kim, S. T., Son, H. J., Choi, J. H., Ji, I. J. & Hwang, O. Vertical grid test and modified horizontal grid test are sensitive methods for evaluating motor dysfunctions in the MPTP mouse model of Parkinson's disease. *Brain Res.* **1306,** 176–83 (2010).

421. Ferreira-Gomes, J., Adães, S. & Castro-Lopes, J. M. Assessment of movement-evoked pain in osteoarthritis by the knee-bend and CatWalk tests: a clinically relevant study. *J. Pain* **9,** 945–54 (2008).

422. Ferreira-Gomes, J., Adães, S., Mendonça, M. & Castro-Lopes, J. M. Analgesic effects of lidocaine, morphine and diclofenac on movement-induced nociception, as assessed by the Knee-Bend and CatWalk tests in a rat model of osteoarthritis. *Pharmacol. Biochem. Behav.* **101,** 617–24 (2012).

423. Apostolova, I., Irintchev, A. & Schachner, M. Tenascin-R restricts posttraumatic remodeling of motoneuron innervation and functional recovery after spinal cord injury in adult mice. *J. Neurosci.* **26,** 7849–59 (2006).

424. Fey, A., Schachner, M. & Irintchev, A. A novel motion analysis approach reveals late recovery in C57BL/6 mice and deficits in NCAM-deficient mice after sciatic nerve crush. *J. Neurotrauma* **27,** 815–28 (2010).

425. Pajoohesh-Ganji, A., Byrnes, K. R., Fatemi, G. & Faden, A. I. A combined scoring method to assess behavioral recovery after mouse spinal cord injury. *Neurosci. Res.* **67,** 117–25 (2010).

426. Soblosky, J. S., Colgin, L. L., Chorney-Lane, D., Davidson, J. F. & Carey, M. E. Ladder beam and camera video recording system for evaluating forelimb and hindlimb deficits after sensorimotor cortex injury in rats. *J. Neurosci. Methods* **78,** 75–83 (1997).

427. Z'Graggen, W. J., Metz, G. A., Kartje, G. L., Thallmair, M. & Schwab, M. E. Functional recovery and enhanced corticofugal plasticity after unilateral pyramidal tract lesion and blockade of myelin-associated neurite growth inhibitors in adult rats. *J. Neurosci.* **18,** 4744–57 (1998).

428. Metz, G. A. & Whishaw, I. Q. Cortical and subcortical lesions impair skilled walking in the ladder rung walking test: a new task to evaluate fore- and hindlimb stepping, placing, and co-ordination. *J. Neurosci. Methods* **115,** 169–79 (2002).

429. Riek-Burchardt, M., Henrich-Noack, P., Metz, G. A. & Reymann, K. G. Detection of chronic sensorimotor impairments in the ladder rung walking task in rats with endothelin-1-induced mild focal ischemia. *J. Neurosci. Methods* **137,** 227–33 (2004).

430. Metz, G. A. & Whishaw, I. Q. The ladder rung walking task: a scoring system and its practical application. *J. Vis. Exp.* 4–7 (2009). doi:10.3791/1204

431. Lashley, K. S. *Brain mechanisms and intelligence: A quantitative study of injuries to the brain*. (University of Chicago Press, 1929).

432. Denenberg, V. H., Talgo, N., Carroll, D. A., Freter, S. & Deni, R. A computer-aided procedure for measuring Lashley III maze performance. *Physiol. Behav.* **50,** 857–61 (1991).

433. Blizard, D. A. *et al.* "Return to home cage" as a reward for maze learning in young and old genetically heterogeneous mice. *Comp. Med.* **56,** 196–201 (2006).

434. Bressler, A., Blizard, D. & Andrews, A. Low-stress route learning using the Lashley III maze in mice. *J. Vis. Exp.* 1–7 (2010). doi:10.3791/1786

435. Lubow, R. E. & Moore, A. U. Latent inhibition: the effect of nonreinforced pre-exposure to the conditional stimulus. *J. Comp. Physiol. Psychol.* **52,** 415–9 (1959).

436. Lubow, R. E. Latent inhibition. *Psychol. Bull.* **79,** 398–407 (1973).

437. Weiss, K. R. & Brown, B. L. Latent inhibition: a review and a new hypothesis. *Acta Neurobiol. Exp. (Wars).* **34,** 301–16 (1974).

438. Weiner, I. Latent inhibition. *Curr. Protoc. Neurosci.* **Chapter 8,** Unit 8.13 (2001).

439. Manrique, T. *et al.* Time of day-dependent latent inhibition of conditioned taste aversions in rats. *Neurobiol. Learn. Mem.* **82,** 77–80 (2004).

440. Castagné, V., Moser, P. C. & Porsolt, R. D. Preclinical behavioral models for predicting antipsychotic activity. *Adv. Pharmacol.* **57,** 381–418 (2009).

441. Schallert, T., De Ryck, M., Whishaw, I. Q., Ramirez, V. D. & Teitelbaum, P. Excessive bracing reactions and their control by atropine and L-DOPA in an animal analog of Parkinsonism. *Exp. Neurol.* **64,** 33–43 (1979).

442. Jentsch, J. D., Aarde, S. M. & Seu, E. Effects of atomoxetine and methylphenidate on performance of a lateralized reaction time task in rats. *Psychopharmacology (Berl).* **202,** 497–504 (2009).

443. Jentsch, J. D. Genetic vasopressin deficiency facilitates performance of a lateralized reaction-time task: altered attention and motor processes. *J. Neurosci.* **23,** 1066–71 (2003).

444. Jentsch, J. D. Pre-clinical models of cognitive dysfunction in schizophrenia: new avenues to addressing unmet needs. *Clin. Neurosci. Res.* **3,** 303–313 (2003).

445. Carli, M., Evenden, J. L. & Robbins, T. W. Depletion of unilateral striatal dopamine impairs initiation of contralateral actions and not sensory attention. *Nature* **313,** 679–82 (1985).

446. Schallert, T., Woodlee, M. T. & Fleming, S. M. in *Pharmacol. Cereb. Ischemia* (Krigelstein, J. & Klumpp, S.) 201–216 (Medpharm Scientific Publishers, 2002).

447. Schallert, T. Behavioral tests for preclinical intervention assessment. *NeuroRx* **3,** 497–504 (2006).

448. Brooks, S. P. & Dunnett, S. B. Tests to assess motor phenotype in mice: a user's guide. *Nat. Rev. Neurosci.* **10,** 519–29 (2009).

449. Crawley, J. & Goodwin, F. K. Preliminary report of a simple animal behavior model for the anxiolytic effects of benzodiazepines. *Pharmacol. Biochem. Behav.* **13,** 167–70 (1980).

450. Ambrogi Lorenzini, C., Bucherelli, C. & Giachetti, A. Passive and active avoidance behavior in the light-dark box test. *Physiol. Behav.* **32,** 687–9 (1984).

451. Ambrogi Lorenzini, C., Bucherelli, C. & Giachetti, A. Some factors influencing conditioned and spontaneous behavior of rats in the light-dark box test. *Physiol. Behav.* **36,** 97–101 (1986).

452. Kilfoil, T., Michel, A., Montgomery, D. & Whiting, R. L. Effects of anxiolytic and anxiogenic drugs on exploratory activity in a simple model of anxiety in mice. *Neuropharmacology* **28,** 901–5 (1989).

453. Hascoët, M., Bourin, M. & Nic Dhonnchadha, B. A. The mouse light-dark paradigm: a review. *Prog. Neuropsychopharmacol. Biol. Psychiatry* **25,** 141–66 (2001).

454. Bourin, M. & Hascoët, M. The mouse light/dark box test. *Eur. J. Pharmacol.* **463,** 55–65 (2003).

455. Augustsson, H., van de Weerd, H. A., Kruitwagen, C. L. J. J. & Baumans, V. Effect of enrichment on variation and results in the light/dark test. *Lab. Anim.* **37,** 328–40 (2003).

456. Miller, S. M., Piasecki, C. C. & Lonstein, J. S. Use of the light-dark box to compare the anxiety-related behavior of virgin and postpartum female rats. *Pharmacol. Biochem. Behav.* **100,** 130–7 (2011).

457. Oomen, C. A. *et al.* The touchscreen operant platform for testing working memory and pattern separation in rats and mice. *Nat. Protoc.* **8,** 2006–21 (2013).

458. McTighe, S. M., Mar, A. C., Romberg, C., Bussey, T. J. & Saksida, L. M. A new touchscreen test of pattern separation: effect of hippocampal lesions. *Neuroreport* **20,** 881–5 (2009).

459. Gilbert, P. E., Kesner, R. P. & DeCoteau, W. E. Memory for spatial location: role of the hippocampus in mediating spatial pattern separation. *J. Neurosci.* **18,** 804–10 (1998).

460. Eilam, D. & Szechtman, H. Dosing regimen differentiates sensitization of locomotion and mouthing to D2 agonist quinpirole. *Pharmacol. Biochem. Behav.* **36,** 989–91 (1990).

461. Araujo, N. P. *et al.* The importance of housing conditions on behavioral sensitization and tolerance to ethanol. *Pharmacol. Biochem. Behav.* **82,** 40–5 (2005).

462. Valjent, E. *et al.* Mechanisms of locomotor sensitization to drugs of abuse in a two-injection protocol. *Neuropsychopharmacology* **35,** 401–15 (2010).

463. Smith, R. R. *et al.* The Louisville Swim Scale: a novel assessment of hindlimb function following spinal cord injury in adult rats. *J. Neurotrauma* **23,** 1654–70 (2006).

464. Poling, A., Cleary, J. & Monaghan, M. Burying by rats in response to aversive and nonaversive stimuli. *J. Exp. Anal. Behav.* **35,** 31–44 (1981).

465. Broekkamp, C. L., Rijk, H. W., Joly-Gelouin, D. & Lloyd, K. L. Major tranquillizers can be distinguished from minor tranquillizers on the basis of effects on marble burying and swim-induced grooming in mice. *Eur. J. Pharmacol.* **126,** 223–9 (1986).

466. Njung'e, K. & Handley, S. L. Evaluation of marble-burying behavior as a model of anxiety. *Pharmacol. Biochem. Behav.* **38,** 63–7 (1991).

467. Gyertyán, I. Analysis of the marble burying response: marbles serve to measure digging rather than evoke burying. *Behav. Pharmacol.* **6,** 24–31 (1995).

468. Londei, T., Valentini, A. M. & Leone, V. G. Investigative burying by laboratory mice may involve non-functional, compulsive, behaviour. *Behav. Brain Res.* **94,** 249–54 (1998).

469. Deacon, R. M. J. Digging and marble burying in mice: simple methods for in vivo identification of biological impacts. *Nat. Protoc.* **1,** 122–4 (2006).

470. Thomas, A. *et al.* Marble burying reflects a repetitive and perseverative behavior more than novelty-induced anxiety. *Psychopharmacology (Berl).* **204,** 361–73 (2009).

471. Hayashi, E., Kuratani, K., Kinoshita, M. & Hara, H. Pharmacologically distinctive behaviors other than burying marbles during the marble burying test in mice. *Pharmacology* **86,** 293–6 (2010).

472. Albelda, N. & Joel, D. Current animal models of obsessive compulsive disorder: an update. *Neuroscience* **211,** 83–106 (2012).

473. Albelda, N. & Joel, D. Animal models of obsessive-compulsive disorder: exploring pharmacology and neural substrates. *Neurosci. Biobehav. Rev.* **36,** 47–63 (2012).

474. Morris, R. G. M. Spatial Localization Does Not Require the Presence of Local Cues. *Learn. Motiv.* **260,** 239–260 (1981).

475. Morris, R. Developments of a water-maze procedure for studying spatial learning in the rat. *J. Neurosci. Methods* **11,** 47–60 (1984).

476. Brandeis, R., Brandys, Y. & Yehuda, S. The use of the Morris Water Maze in the study of memory and learning. *Int. J. Neurosci.* **48,** 29–69 (1989).

477. Whishaw, I. Q. & Tomie, J. A. Of mice and mazes: similarities between mice and rats on dry land but not water mazes. *Physiol. Behav.* **60,** 1191–7 (1996).

478. Dalm, S., Grootendorst, J., de Kloet, E. R. & Oitzl, M. S. Quantification of swim patterns in the Morris water maze. *Behav. Res. Methods. Instrum. Comput.* **32,** 134–9 (2000).

479. D'Hooge, R. & De Deyn, P. P. Applications of the Morris water maze in the study of learning and memory. *Brain Res. Brain Res. Rev.* **36,** 60–90 (2001).

480. Graziano, A. Automatic recognition of explorative strategies in the Morris water maze. *J. Neurosci. Methods* **130,** 33–44 (2003).

481. Enthoven, L., Dalm, S., de Kloet, E. R. & Oitzl, M. S. Swim posture of mice does not affect performance in the water maze. *Brain Res.* **1003,** 36–41 (2004).

482. Stasko, M. R. & Costa, A. C. S. Experimental parameters affecting the Morris water maze performance of a mouse model of Down syndrome. *Behav. Brain Res.* **154,** 1–17 (2004).

483. Wenk, G. L. Assessment of spatial memory using the radial arm maze and Morris water maze. *Curr. Protoc. Neurosci.* **Chapter 8,** Unit 8.5A (2004).

484. Grigoryan, G., Hodges, L. H. & Gray, J. Effects of vibrissae removal on search accuracy in the water maze. *Neurosci. Behav. Physiol.* **35,** 133–7 (2005).

485. Choi, S. H., Woodlee, M. T., Hong, J. J. & Schallert, T. A simple modification of the water maze test to enhance daily detection of spatial memory in rats and mice. *J. Neurosci. Methods* **156,** 182–93 (2006).

486. Korz, V. Water maze swim path analysis based on tracking coordinates. *Behav. Res. Methods* **38,** 522–8 (2006).

487. Thompson, H. J. *et al.* Cognitive evaluation of traumatically brain-injured rats using serial testing in the Morris water maze. *Restor. Neurol. Neurosci.* **24,** 109–14 (2006).

488. Vorhees, C. V & Williams, M. T. Morris water maze: procedures for assessing spatial and related forms of learning and memory. *Nat. Protoc.* **1,** 848–58 (2006).

489. Nunez, J. Morris Water Maze Experiment. *J. Vis. Exp.* 12–14 (2008).

490. Bromley-Brits, K., Deng, Y. & Song, W. Morris water maze test for learning and memory deficits in Alzheimer's disease model mice. *J. Vis. Exp.* 2–6 (2011).

491. Bingham, D., Martin, S. J., Macrae, I. M. & Carswell, H. V. O. Watermaze performance after middle cerebral artery occlusion in the rat: the role of sensorimotor versus memory impairments. *J. Cereb. Blood Flow Metab.* **32,** 989–999 (2012).

492. Meyerson, B. J., Augustsson, H., Berg, M. & Roman, E. The Concentric Square Field: a multivariate test arena for analysis of explorative strategies. *Behav. Brain Res.* **168,** 100–13 (2006).

493. Roman, E., Meyerson, B. J., Hyytiä, P. & Nylander, I. The multivariate concentric square field test reveals different behavioural profiles in male AA and ANA rats with regard to risk taking and environmental reactivity. *Behav. Brain Res.* **183,** 195–205 (2007).

494. Ekmark-Lewén, S., Lewén, A., Meyerson, B. J. & Hillered, L. The multivariate concentric square field test reveals behavioral profiles of risk taking, exploration, and cognitive impairment in mice subjected to traumatic brain injury. *J. Neurotrauma* **27,** 1643–55 (2010).

495. Crozier, W. J. & Pincus, G. The Geotropic Conduct of Young Rats. *J. Gen. Physiol.* **10,** 257–69 (1926).

496. Alberts, J. R., Motz, B. a & Schank, J. C. Positive geotaxis in infant rats (Rattus norvegicus): a natural behavior and a historical correction. *J. Comp. Psychol.* **118,** 123–32 (2004).

497. Li, X. *et al.* Chronic behavioral testing after focal ischemia in the mouse: functional recovery and the effects of gender. *Exp. Neurol.* **187,** 94–104 (2004).

498. Motz, B. A. & Alberts, J. R. The validity and utility of geotaxis in young rodents. *Neurotoxicol. Teratol.* **27,** 529–33 (2005).

499. Moser, V. C. Response to open commentary, "Validity and Utility of Geotaxis" by Motz and Alberts. *Neurotoxicol. Teratol.* **27,** 539–40; author reply 543–4 (2005).

500. Kreider, J. C. & Blumberg, M. S. Geotaxis and beyond: commentary on Motz and Alberts (2005). *Neurotoxicol. Teratol.* **27,** 535–7; author reply 543–4 (2005).

501. Ferrara, A., El Bejaoui, S., Seyen, S., Tirelli, E. & Plumier, J.-C. The usefulness of operant conditioning procedures to assess long-lasting deficits following transient focal ischemia in mice. *Behav. Brain Res.* **205,** 525–34 (2009).

502. Rosvold, H. E. & Walker, A. M. Effect of electroconvulsive shocks on nest building in the male albino rat. *Proc. Soc. Exp. Biol. Med.* **72,** 270–2 (1949).

503. Schneider, C. W. & Chenoweth, M. B. Effects of hallucinogenic and other drugs on the nest-building behaviour of mice. *Nature* **225,** 1262–3 (1970).

504. Van Loo, P. L. P. & Baumans, V. The importance of learning young: the use of nesting material in laboratory rats. *Lab. Anim.* **38,** 17–24 (2004).

505. Deacon, R. M. J. Assessing nest building in mice. *Nat. Protoc.* **1,** 1117–9 (2006).

506. Irwin, S. Comprehensive observational assessment: Ia. A systematic, quantitative procedure for assessing the behavioral and physiologic state of the mouse. *Psychopharmacologia* **13,** 222–57 (1968).

507. Bederson, J. B. *et al.* Rat middle cerebral artery occlusion: evaluation of the model and development of a neurologic examination. *Stroke* **17,** 472–476 (1986).

508. McIntosh, T. K. *et al.* Traumatic brain injury in the rat: characterization of a lateral fluid-percussion model. *Neuroscience* **28,** 233–44 (1989).

509. Shohami, E., Novikov, M. & Bass, R. Long-term effect of HU-211, a novel non-competitive NMDA antagonist, on motor and memory functions after closed head injury in the rat. *Brain Res.* **674,** 55–62 (1995).

510. Murai, H. *et al.* Twofold overexpression of human beta-amyloid precursor proteins in transgenic mice does not affect the neuromotor, cognitive, or neurodegenerative sequelae following experimental brain injury. *J. Comp. Neurol.* **392,** 428–38 (1998).

511. Hunter, A. J. *et al.* Functional assessments in mice and rats after focal stroke. *Neuropharmacology* **39,** 806–16 (2000).

512. Zanier, E. R. *et al.* Human umbilical cord blood mesenchymal stem cells protect mice brain after trauma. *Crit. Care Med.* **39,** 2501–10 (2011).

513. Tomasi, S., Sarmientos, P., Giorda, G., Gurewich, V. & Vercelli, A. Mutant prourokinase with adjunctive C1-inhibitor is an effective and safer alternative to tPA in rat stroke. *PLoS One* **6,** e21999 (2011).

514. Ren, Z. *et al.* "Hit & Run" model of closed-skull traumatic brain injury (TBI) reveals complex patterns of post-traumatic AQP4 dysregulation. *J. Cereb. Blood Flow Metab.* **33,** 834–45 (2013).

515. Ennaceur, A. & Delacour, J. A new one-trial test for neurobiological studies of memory in rats. 1: Behavioral data. *Behav. Brain Res.* **31,** 47–59 (1988).

516. Ennaceur, A., Cavoy, A., Costa, J. C. & Delacour, J. A new one-trial test for neurobiological studies of memory in rats. II: Effects of piracetam and pramiracetam. *Behav. Brain Res.* **33,** 197–207 (1989).

517. Mitchell, J. B. & Laiacona, J. The medial frontal cortex and temporal memory: tests using spontaneous exploratory behaviour in the rat. *Behav. Brain Res.* **97,** 107–13 (1998).

518. Silvers, J. M., Harrod, S. B., Mactutus, C. F. & Booze, R. M. Automation of the novel object recognition task for use in adolescent rats. *J. Neurosci. Methods* **166,** 99–103 (2007).

519. Rutten, K. *et al.* Automated scoring of novel object recognition in rats. *J. Neurosci. Methods* **171,** 72–7 (2008).

520. Ennaceur, A. One-trial object recognition in rats and mice: methodological and theoretical issues. *Behav. Brain Res.* **215,** 244–54 (2010).

521. Mathiasen, J. R. & DiCamillo, A. Novel object recognition in the rat: a facile assay for cognitive function. *Curr. Protoc. Pharmacol.* **Chapter 5,** Unit 5.59 (2010).

522. Antunes, M. & Biala, G. The novel object recognition memory: neurobiology, test procedure, and its modifications. *Cogn. Process.* **13,** 93–110 (2012).

523. Zhang, R. *et al.* Novel object recognition as a facile behavior test for evaluating drug effects in AβPP/PS1 Alzheimer's disease mouse model. *J. Alzheimers. Dis.* **31,** 801–12 (2012).

524. White, T. L. & Youngentob, S. L. The effect of NMDA-NR2B receptor subunit over-expression on olfactory memory task performance in the mouse. *Brain Res.* **1021,** 1–7 (2004).

525. Scott, G. A., Mtetwa, M. & Lehmann, H. Novel odour recognition memory is independent of the hippocampus in rats. *Exp. Brain Res.* **224,** 199–209 (2013).

526. Clark, R. E., Broadbent, N. J. & Squire, L. R. Hippocampus and remote spatial memory in rats. *Hippocampus* **15,** 260–72 (2005).

527. Young, J. W. *et al.* The odour span task: a novel paradigm for assessing working memory in mice. *Neuropharmacology* **52,** 634–45 (2007).

528. Rushforth, S. L., Allison, C., Wonnacott, S. & Shoaib, M. Subtype-selective nicotinic agonists enhance olfactory working memory in normal rats: a novel use of the odour span task. *Neurosci. Lett.* **471,** 114–8 (2010).

529. April, L. B., Bruce, K. & Galizio, M. The Magic Number 70 (plus or minus 20): Variables Determining Performance in the Rodent Odor Span Task. *Learn. Motiv.* **44,** 143–158 (2013).

530. Dudchenko, P. A., Wood, E. R. & Eichenbaum, H. Neurotoxic hippocampal lesions have no effect on odor span and little effect on odor recognition memory but produce significant impairments on spatial span, recognition, and alternation. *J. Neurosci.* **20,** 2964–77 (2000).

531. Dudchenko, P. A. An overview of the tasks used to test working memory in rodents. *Neurosci. Biobehav. Rev.* **28,** 699–709 (2004).

532. Pfaffmann, C. An olfactometer for the rat. *Science* **128,** 1007–8 (1958).

533. Howard, W. E., Palmateer, S. D. & Marsh, R. E. A body capacitor-olfactometer for squirrels and rats. *J. Mammal.* **50,** 771–6 (1969).

534. Slotnick, B. M. & Nigrosh, B. J. Olfactory stimulus control evaluated in a small animal olfactometer. *Percept. Mot. Skills* **39,** 583–97 (1974).

535. Slotnick, B. M. & Schoonover, F. W. Olfactory pathways and the sense of smell. *Neurosci. Biobehav. Rev.* **16,** 453–72 (1992).

536. Owens, J. G. *et al.* Design and evaluation of an olfactometer for the assessment of 3-methylindole-induced hyposmia. *Fundam. Appl. Toxicol.* **33,** 60–70 (1996).

537. Bodyak, N. & Slotnick, B. Performance of mice in an automated olfactometer: odor detection, discrimination and odor memory. *Chem. Senses* **24,** 637–45 (1999).

538. Slotnick, B., Cockerham, R. & Pickett, E. Olfaction in olfactory bulbectomized rats. *J. Neurosci.* **24,** 9195–200 (2004).

539. Slotnick, B. & Restrepo, D. Olfactometry with mice. *Curr. Protoc. Neurosci.* **Chapter 8,** Unit 8.20 (2005).

540. Gamble, K. R. & Smith, D. W. Discrimination of "odorless" mineral oils alone and as diluents by behaviorally trained mice. *Chem. Senses* **34,** 559–63 (2009).

541. Haynes, N. B. & Purvis, K. A multiple choice apparatus based on an air stream design for studies on olfactory discrimination in rodents. *J. Physiol.* **222,** 10P–12P (1972).

542. Mihalick, S. M., Langlois, J. C., Krienke, J. D. & Dube, W. V. An olfactory discrimination procedure for mice. *J. Exp. Anal. Behav.* **73,** 305–18 (2000).

543. Uchida, N. & Mainen, Z. F. Speed and accuracy of olfactory discrimination in the rat. *Nat. Neurosci.* **6,** 1224–9 (2003).

544. Enwere, E. *et al.* Aging results in reduced epidermal growth factor receptor signaling, diminished olfactory neurogenesis, and deficits in fine olfactory discrimination. *J. Neurosci.* **24,** 8354–65 (2004).

545. Abraham, N. M. *et al.* Maintaining accuracy at the expense of speed: stimulus similarity defines odor discrimination time in mice. *Neuron* **44,** 865–76 (2004).

546. Rinberg, D., Koulakov, A. & Gelperin, A. Speed-accuracy tradeoff in olfaction. *Neuron* **51,** 351–8 (2006).

547. Larson, J., Kim, D., Patel, R. C. & Floreani, C. Olfactory discrimination learning in mice lacking the fragile X mental retardation protein. *Neurobiol. Learn. Mem.* **90,** 90–102 (2008).

548. Belnoue, L., Grosjean, N., Abrous, D. N. & Koehl, M. A critical time window for the recruitment of bulbar newborn neurons by olfactory discrimination learning. *J. Neurosci.* **31,** 1010–6 (2011).

549. Devore, S., Lee, J. & Linster, C. Odor preferences shape discrimination learning in rats. *Behav. Neurosci.* **127,** 498–504 (2013).

550. Gregg, B. & Thiessen, D. D. A simple method of olfactory discrimination of urines for the Mongolian gerbil, Meriones unguiculatus. *Physiol. Behav.* **26,** 1133–6 (1981).

551. Cleland, T. A., Morse, A., Yue, E. L. & Linster, C. Behavioral models of odor similarity. *Behav. Neurosci.* **116,** 222–231 (2002).

552. Roman, F. S., Marchetti, E., Bouquerel, a & Soumireu-Mourat, B. The olfactory tubing maze: a new apparatus for studying learning and memory processes in mice. *J. Neurosci. Methods* **117,** 173–81 (2002).

553. Chaillan, F. A., Marchetti, E., Soumireu-Mourat, B. & Roman, F. S. Selective impairment of subcategories of long-term memory in mice with hippocampal lesions accessed by the olfactory tubing maze. *Behav. Brain Res.* **158,** 285–92 (2005).

554. Restivo, L., Chaillan, F. A., Ammassari-Teule, M., Roman, F. S. & Marchetti, E. Strain differences in rewarded discrimination learning using the olfactory tubing maze. *Behav. Genet.* **36,** 923–34 (2006).

555. Haggbloom, S. J., Brunner, R. L. & Bayer, S. A. Effects of hippocampal granule-cell agenesis on acquisition of escape from fear and one-way active-avoidance responses. *J. Comp. Physiol. Psychol.* **86,** 447–57 (1974).

556. Myslivecek, J. Ontogeny of active avoidance learning and memory. *Act. Nerv. Super. (Praha).* **18,** 291–301 (1976).

557. Weinberger, S. B., Koob, G. F. & Martinez, J. L. Differences in one-way active avoidance learning in mice of three inbred strains. *Behav. Genet.* **22,** 177–88 (1992).

558. Hall, C. S. Emotional behavior in the rat. I. Defecation and urination as measures of individual differences in emotionality. *J. Comp. Psychol.* **18,** 385–403 (1934).

559. Holland, H. C. & Weldon, E. A note on a new technique of recording ambulation in the open field test and its validation. *Acta Psychol. (Amst).* **28,** 293–300 (1968).

560. Walsh, R. N. & Cummins, R. A. The Open-Field Test: a critical review. *Psychol. Bull.* **83,** 482–504 (1976).

561. Rodgers, R. J. Animal models of "anxiety": where next? *Behav. Pharmacol.* **8,** 477–96; discussion 497–504 (1997).

562. Prut, L. & Belzung, C. The open field as a paradigm to measure the effects of drugs on anxiety-like behaviors: a review. *Eur. J. Pharmacol.* **463,** 3–33 (2003).

563. Stanford, S. C. The Open Field Test: reinventing the wheel. *J. Psychopharmacol.* **21,** 134–5 (2007).

564. Blizard, D. A., Takahashi, A., Galsworthy, M. J., Martin, B. & Koide, T. Test standardization in behavioural neuroscience: a response to Stanford. *J. Psychopharmacol.* **21,** 136–9 (2007).

565. Stanford, S. C. Open fields (unlike wheels) can be any shape but still miss the target. *J. Psychopharmacol.* **21,** 144 (2007).

566. Rodgers, R. J. More haste, considerably less speed. *J. Psychopharmacol.* **21,** 141–3 (2007).

567. Overstreet, D. H. The Open Field Test for two. *J. Psychopharmacol.* **21,** 140 (2007).

568. Kulikov, A. V, Tikhonova, M. A. & Kulikov, V. A. Automated measurement of spatial preference in the open field test with transmitted lighting. *J. Neurosci. Methods* **170,** 345–51 (2008).

569. Leroy, T., Silva, M., D'Hooge, R., Aerts, J.-M. & Berckmans, D. Automated gait analysis in the open-field test for laboratory mice. *Behav. Res. Methods* **41,** 148–53 (2009).

570. Clark, R. E., Reinagel, P., Broadbent, N. J., Flister, E. D. & Squire, L. R. Intact performance on feature-ambiguous discriminations in rats with lesions of the perirhinal cortex. *Neuron* **70,** 132–40 (2011).

571. Histed, M. H., Carvalho, L. a & Maunsell, J. H. R. Psychophysical measurement of contrast sensitivity in the behaving mouse. *J. Neurophysiol.* **107,** 758–65 (2012).

572. Keller, J., Strasburger, H., Cerutti, D. T. & Sabel, B. A. Assessing spatial vision - automated measurement of the contrast-sensitivity function in the hooded rat. *J. Neurosci. Methods* **97,** 103–10 (2000).

573. Skinner, B. F. & Morse, W. H. Fixed-interval reinforcement of running in a wheel. *J. Exp. Anal. Behav.* **1,** 371–9 (1958).

574. Sharma, S., Hryhorczuk, C. & Fulton, S. Progressive-ratio responding for palatable high-fat and high-sugar food in mice. *J. Vis. Exp.* e3754 (2012). doi:10.3791/3754

575. Bussey, T. J. *et al.* The touchscreen cognitive testing method for rodents: how to get the best out of your rat. *Learn. Mem.* **15,** 516–23 (2008).

576. Bussey, T. J., Saksida, L. M. & Rothblat, L. A. Discrimination of computer-graphic stimuli by mice: A method for the behavioral characterization of transgenic and gene-knockout models. *Behav. Neurosci.* **115,** 957–960 (2001).

577. Gutnikov, S. A., Barnes, J. C. & Rawlins, J. N. Working memory tasks in five-choice operant chambers: use of relative and absolute spatial memories. *Behav. Neurosci.* **108,** 899–910 (1994).

578. Baron, S. P. & Meltzer, L. T. Mouse strains differ under a simple schedule of operant learning. *Behav. Brain Res.* **118,** 143–52 (2001).

579. Delcasso, S., Jeantet, Y. & Cho, Y. H. A new test for long-term spatial memory using an operant chamber in mice. *Behav. Brain Res.* **178,** 200–7 (2007).

580. Olsen, C. M. & Winder, D. G. Operant sensation seeking in the mouse. *J. Vis. Exp.* 1–5 (2010).

581. Mar, A. C. *et al.* The touchscreen operant platform for assessing executive function in rats and mice. *Nat. Protoc.* **8,** 1985–2005 (2013).

582. Bunsey, M. & Eichenbaum, H. Critical role of the parahippocampal region for paired-associate learning in rats. *Behav. Neurosci.* **107,** 740–7 (1993).

583. Kesner, R. P., Hunsaker, M. R. & Gilbert, P. E. The role of CA1 in the acquisition of an object-trace-odor paired associate task. *Behav. Neurosci.* **119,** 781–6 (2005).

584. Gibb, S. J., Wolff, M. & Dalrymple-Alford, J. C. Odour-place paired-associate learning and limbic thalamus: comparison of anterior, lateral and medial thalamic lesions. *Behav. Brain Res.* **172,** 155–68 (2006).

585. Van Dijck, A., Vloeberghs, E., Van Dam, D., Staufenbiel, M. & De Deyn, P. P. Evaluation of the APP23-model for Alzheimer's disease in the odour paired-associate test for hippocampus-dependent memory. *Behav. Brain Res.* **190,** 147–51 (2008).

586. Talpos, J. C., Winters, B. D., Dias, R., Saksida, L. M. & Bussey, T. J. A novel touchscreen-automated paired-associate learning (PAL) task sensitive to pharmacological manipulation of the hippocampus: a translational rodent model of cognitive impairments in neurodegenerative disease. *Psychopharmacology (Berl).* **205,** 157–68 (2009).

587. Bartko, S. J., Vendrell, I., Saksida, L. M. & Bussey, T. J. A computer-automated touchscreen paired-associates learning (PAL) task for mice: impairments following administration of scopolamine or dicyclomine and improvements following donepezil. *Psychopharmacology (Berl).* **214,** 537–48 (2011).

588. Bussey, T. J., Barch, D. M. & Baxter, M. G. Testing long-term memory in animal models of schizophrenia: Suggestions from CNTRICS. *Neurosci. Biobehav. Rev.* (2013). doi:10.1016/j.neubiorev.2013.06.005

589. Young, J. W., Jentsch, J. D., Bussey, T. J., Wallace, T. L. & Hutcheson, D. M. Consideration of species differences in developing novel molecules as cognition enhancers. *Neurosci. Biobehav. Rev.* **37,** 2181–93 (2013).

590. Bronson, F. H. & Eleftheriou, B. E. Adrenal response to fighting in mice: separation of physical and psychological causes. *Science* **147,** 627–8 (1965).

591. Kudryavtseva, N. N. Use of the "partition" test in behavioral and pharmacological experiments. *Neurosci. Behav. Physiol.* **33,** 461–71 (2003).

592. Semple, B. D., Canchola, S. A. & Noble-Haeusslein, L. J. Deficits in Social Behavior Emerge during Development after Pediatric Traumatic Brain Injury in Mice. *J. Neurotrauma* **29,** 2672–83 (2012).

593. Jarvik, M. E. & Essman, W. B. A Simple One-Trial Learning Situation for Mice. *Psychol. Rep.* **6,** 290 (1960).

594. Kurtz, K. H. & Pearl, J. The effects of prior fear experiences on acquired-drive learning. *J. Comp. Physiol. Psychol.* **53,** 201–6 (1960).

595. Isaacson, R. L. & Wickelgren, W. O. Hippocampal ablation and passive avoidance. *Science* **138,** 1104–6 (1962).

596. Van der Poel, A. M. Ethological study of the behaviour of the albino rat in a passive-avoidance test. *Acta Physiol. Pharmacol. Neerl.* **14,** 503–5 (1967).

597. Boitano, J. J. & Isaacson, R. L. Effects of variation in shock-intensity on the behavior of dorsal-hippocampectomized rats in two passive-avoidance situations. *Am. J. Psychol.* **80,** 73–80 (1967).

598. Jarvik, M. E. & Kopp, R. An improved one-trial passive avoidance learning situation. *Psychol. Rep.* **21,** 221–4 (1967).

599. Randall, R. K. & Riccio, D. C. Fear and punishment as determinants of passive-avoidance responding. *J. Comp. Physiol. Psychol.* **69,** 550–3 (1969).

600. Carew, T. J. Do passive-avoidance tasks permit assessment of retrograde amnesia in rats? *J. Comp. Physiol. Psychol.* **72,** 267–71 (1970).

601. Ader, R., Weijnen, J. & Moleman, P. Retention of a passive avoidance response as a function of the intensity and duration of electric shock. *Psychon. Sci.* **26,** 126–8 (1972).

602. Davies, J. A., Navaratnam, V. & Redfern, P. H. A 24-hour rhythm in passive-avoidance behaviour in rats. *Psychopharmacologia* **32,** 211–4 (1973).

603. Staubli, U. & Huston, J. P. Up-hill avoidance: a new passive-avoidance task. *Physiol. Behav.* **22,** 775–6 (1979).

604. Van Oyen, H. G., Van De Poll, N. E. & De Bruin, J. P. Sex, age and shock-intensity as factors in passive avoidance. *Physiol. Behav.* **23,** 915–8 (1979).

605. Stehouwer, D. J. & Campbell, B. A. Ontogeny of passive avoidance: role of task demands and development of species-typical behaviors. *Dev. Psychobiol.* **13,** 385–98 (1980).

606. Van Haaren, F. & van de Poll, N. E. Effects of light intensity on passive avoidance behavior of male and female Wistar rats. *Physiol. Behav.* **36,** 123–5 (1986).

607. Kameyama, T., Nabeshima, T. & Kozawa, T. Step-down-type passive avoidance- and escape-learning method. Suitability for experimental amnesia models. *J. Pharmacol. Methods* **16,** 39–52 (1986).

608. Croiset, G., Veldhuis, H. D., Ballieux, R. E., de Wied, D. & Heijnen, C. J. The impact of mild emotional stress induced by the passive avoidance procedure on immune reactivity. *Ann. N. Y. Acad. Sci.* **496,** 477–84 (1987).

609. Allweis, C., Chernichovsky, D. & Shulz, D. Exponential data-analysis of passive-avoidance behavior in rats and mice. *Pharmacol. Biochem. Behav.* **31,** 803–6 (1988).

610. Myslivecek, J. & Hassmannová, J. Step-down passive avoidance in the rat ontogeny. *Acta Neurobiol. Exp. (Wars).* **51,** 89–96 (1991).

611. Martel, G., Jaffard, R. & Guillou, J.-L. Identification of hippocampus-dependent and hippocampus independent memory components in step-down inhibitory avoidance tasks. *Behav. Brain Res.* **207,** 138–43 (2010).

612. Moriarty, O., Roche, M., McGuire, B. E. & Finn, D. P. Validation of an air-puff passive-avoidance paradigm for assessment of aversive learning and memory in rat models of chronic pain. *J. Neurosci. Methods* **204,** 1–8 (2012).

613. Collins, R. L. On the inheritance of handedness. I. Laterality in inbred mice. *J. Hered.* **59,** 9–12 (1968).

614. Collins, R. L. When left-handed mice live in right-handed worlds. *Science* **187,** 181–4 (1975).

615. Signore, P. *et al.* An assessment of handedness in mice. *Physiol. Behav.* **49,** 701–4 (1991).

616. Waters, N. S. & Denenberg, V. H. A measure of lateral paw preference in the mouse. *Physiol. Behav.* **50,** 853–6 (1991).

617. Waters, N. S. & Denenberg, V. H. Analysis of two measures of paw preference in a large population of inbred mice. *Behav. Brain Res.* **63,** 195–204 (1994).

618. Bulman-Fleming, M. B., Bryden, M. P. & Rogers, T. T. Mouse paw preference: effects of variations in testing protocol. *Behav. Brain Res.* **86,** 79–87 (1997).

619. Güven, M., Elalmiş, D. D., Binokay, S. & Tan, U. Population-level right-paw preference in rats assessed by a new computerized food-reaching test. *Int. J. Neurosci.* **113,** 1675–89 (2003).

620. Balkaya, M., Kröber, J., Gertz, K., Peruzzaro, S. & Endres, M. Characterization of long-term functional outcome in a murine model of mild brain ischemia. *J. Neurosci. Methods* **213,** 179–87 (2013).

621. Rushton, R., Steinberg, H. & Tinson, C. Effects of a single experience on subsequent reactions to drugs. *Br. J. Pharmacol. Chemother.* **20,** 99–105 (1963).

622. De Medinaceli, L., Freed, W. J. & Wyatt, R. J. An index of the functional condition of rat sciatic nerve based on measurements made from walking tracks. *Exp. Neurol.* **77,** 634–43 (1982).

623. Lowdon, I. M., Seaber, A. V & Urbaniak, J. R. An improved method of recording rat tracks for measurement of the sciatic functional index of de Medinaceli. *J. Neurosci. Methods* **24,** 279–81 (1988).

624. Wiethölter, H., Eckert, S. & Stevens, A. Measurement of atactic and paretic gait in neuropathies of rats based on analysis of walking tracks. *J. Neurosci. Methods* **32,** 199–205 (1990).

625. Kunkel-Bagden, E., Dai, H. N. & Bregman, B. S. Methods to assess the development and recovery of locomotor function after spinal cord injury in rats. *Exp. Neurol.* **119,** 153–64 (1993).

626. Klapdor, K., Dulfer, B. G., Hammann, A. & Van der Staay, F. J. A low-cost method to analyse footprint patterns. *J. Neurosci. Methods* **75,** 49–54 (1997).

627. Górska, T., Chojnicka-Gittins, B., Majczyński, H. & Zmysłowski, W. Changes in forelimb-hindlimb coordination after partial spinal lesions of different extent in the rat. *Behav. Brain Res.* **239,** 121–38 (2013).

628. Navarro, X., Kennedy, W. R., Stewart, N. J. & Furcht, L. T. Effects of laminin on functional reinnervation of target organs by regenerating axons. *Neuroreport* **2,** 37–40 (1991).

629. Hori, K., Ozaki, N., Suzuki, S. & Sugiura, Y. Upregulations of P2X(3) and ASIC3 involve in hyperalgesia induced by cisplatin administration in rats. *Pain* **149,** 393–405 (2010).

630. Small, W. S. Notes on the Psychic Development of the Young White Rat. *Am. J. Psychol.* **11,** 80–100 (1899).

631. Thor, D. H. & Holloway, W. R. Social play in juvenile rats: a decade of methodological and experimental research. *Neurosci. Biobehav. Rev.* **8,** 455–64 (1984).

632. Vanderschuren, L. J., Niesink, R. J. & Van Ree, J. M. The neurobiology of social play behavior in rats. *Neurosci. Biobehav. Rev.* **21,** 309–26 (1997).

633. Pellis, S. M., Field, E. F., Smith, L. K. & Pellis, V. C. Multiple differences in the play fighting of male and female rats. Implications for the causes and functions of play. *Neurosci. Biobehav. Rev.* **21,** 105–20 (1997).

634. Pellis, S. M. & Pellis, V. C. Play fighting of rats in comparative perspective: a schema for neurobehavioral analyses. *Neurosci. Biobehav. Rev.* **23,** 87–101 (1998).

635. Foroud, A. & Pellis, S. M. The development of “roughness” in the play fighting of rats: a Laban Movement Analysis perspective. *Dev. Psychobiol.* **42,** 35–43 (2003).

636. Himmler, B. T., Pellis, V. C. & Pellis, S. M. Peering into the dynamics of social interactions: measuring play fighting in rats. *J. Vis. Exp.* e4288 (2013).

637. Ogawa, N., Hirose, Y., Ohara, S., Ono, T. & Watanabe, Y. A simple quantitative bradykinesia test in MPTP-treated mice. *Res. Commun. Chem. Pathol. Pharmacol.* **50,** 435–41 (1985).

638. Matsuura, K., Kabuto, H., Makino, H. & Ogawa, N. Pole test is a useful method for evaluating the mouse movement disorder caused by striatal dopamine depletion. *J. Neurosci. Methods* **73,** 45–8 (1997).

639. Glajch, K. E., Fleming, S. M., Surmeier, D. J. & Osten, P. Sensorimotor assessment of the unilateral 6-hydroxydopamine mouse model of Parkinson’s disease. *Behav. Brain Res.* **230,** 309–16 (2012).

640. Balkaya, M., Kröber, J. M., Rex, A. & Endres, M. Assessing post-stroke behavior in mouse models of focal ischemia. *J. Cereb. Blood Flow Metab.* **33,** 330–8 (2013).

641. Takahashi, L. K., Nakashima, B. R., Hong, H. & Watanabe, K. The smell of danger: a behavioral and neural analysis of predator odor-induced fear. *Neurosci. Biobehav. Rev.* **29,** 1157–67 (2005).

642. Griffith, C. R. A possible case of instinctive behavior in the white rat. *Science* **50,** 166–7 (1919).

643. Griffith, C. R. The behavior of white rats in the presence of cats. *Psychobiology* **2,** 19–28 (1920).

644. Staples, L. G. Predator odor avoidance as a rodent model of anxiety: learning-mediated consequences beyond the initial exposure. *Neurobiol. Learn. Mem.* **94,** 435–45 (2010).

645. Apfelbach, R., Blanchard, C. D., Blanchard, R. J., Hayes, R. A. & McGregor, I. S. The effects of predator odors in mammalian prey species: a review of field and laboratory studies. *Neurosci. Biobehav. Rev.* **29,** 1123–44 (2005).

646. Ison, J. R., McAdam, D. W. & Hammond, G. R. Latency and amplitude changes in the acoustic startle reflex of the rat produced by variation in auditory prestimulation. *Physiol. Behav.* **10,** 1035–9 (1973).

647. Davis, M. & Sheard, M. H. Effects of lysergic acid diethylamide (LSD) on temporal recovery (pre-pulse inhibition) of the acoustic startle response in the rat. *Pharmacol. Biochem. Behav.* **3,** 861–8 (1975).

648. Faraday, M. M. & Grunberg, N. E. The importance of acclimation in acoustic startle amplitude and pre-pulse inhibition testing of male and female rats. *Pharmacol. Biochem. Behav.* **66,** 375–81 (2000).

649. Plappert, C. F., Kuhn, S., Schnitzler, H.-U. & Pilz, P. K. D. Experience increases the prepulse inhibition of the acoustic startle response in mice. *Behav. Neurosci.* **120,** 16–23 (2006).

650. Tunstall, B., Beckett, S. & Mason, R. Ultrasonic vocalisations explain unexpected effects on pre-pulse inhibition responses in rats chronically pre-treated with phencyclidine. *Behav. Brain Res.* **202,** 184–91 (2009).

651. Valsamis, B. & Schmid, S. Habituation and prepulse inhibition of acoustic startle in rodents. *J. Vis. Exp.* e3446 (2011).

652. Ramirez-Moreno, D. F. & Sejnowski, T. J. A computational model for the modulation of the prepulse inhibition of the acoustic startle reflex. *Biol. Cybern.* **106,** 169–76 (2012).

653. Deacon, R. M. & Gardner, C. R. The pull-up test in rats: a simple method for evaluating muscle relaxation. *J. Pharmacol. Methods* **11,** 119–24 (1984).

654. Wilson, N. E., Meert, T. F. & Shephard, R. A. A pharmacological evaluation of the pull-up test for muscle relaxation in rats. *J. Neurosci. Methods* **50,** 359–67 (1993).

655. Olton, D. S. & Samuelson, R. J. Remembrance of Places Passed : Spatial Memory in Rats. *J. Exp. Psychol. Anim. Behav. Process.* **2,** 97–116 (1976).

656. Levin, E. D. Psychopharmacological effects in the radial-arm maze. *Neurosci. Biobehav. Rev.* **12,** 169–75 (1988).

657. Brown, M. F. Does a cognitive map guide choices in the radial-arm maze? *J. Exp. Psychol. Anim. Behav. Process.* **18,** 56–66 (1992).

658. Brown, M. F., Rish, P. A., VonCulin, J. E. & Edberg, J. A. Spatial guidance of choice behavior in the radial-arm maze. *J. Exp. Psychol. Anim. Behav. Process.* **19,** 195–214 (1993).

659. Masuda, Y. *et al.* Radial arm maze behavior in mice when a return to the home cage serves as the reinforcer. *Physiol. Behav.* **56,** 785–8 (1994).

660. He, J., Yamada, K., Nakajima, A., Kamei, H. & Nabeshima, T. Learning and memory in two different reward tasks in a radial arm maze in rats. *Behav. Brain Res.* **134,** 139–48 (2002).

661. Dubreuil, D., Tixier, C., Dutrieux, G. & Edeline, J.-M. Does the radial arm maze necessarily test spatial memory? *Neurobiol. Learn. Mem.* **79,** 109–17 (2003).

662. Paganelli, R. A. *et al.* A novel version of the 8-arm radial maze: effects of cerebral ischemia on learning and memory. *J. Neurosci. Methods* **132,** 9–18 (2004).

663. Wenk, G. L. Assessment of spatial memory using the radial arm maze and Morris water maze. *Curr. Protoc. Neurosci.* **Chapter 8,** Unit 8.5A (2004).

664. Brown, M. F. & Giumetti, G. W. Spatial pattern learning in the radial arm maze. *Learn. Behav.* **34,** 102–8 (2006).

665. Buresová, O., Bures, J., Oitzl, M. S. & Zahálka, A. Radial maze in the water tank: an aversively motivated spatial working memory task. *Physiol. Behav.* **34,** 1003–5 (1985).

666. Hyde, L. A., Hoplight, B. J. & Denenberg, V. H. Water version of the radial-arm maze: learning in three inbred strains of mice. *Brain Res.* **785,** 236–44 (1998).

667. Hyde, L. A., Sherman, G. F. & Denenberg, V. H. Non-spatial water radial-arm maze learning in mice. *Brain Res.* **863,** 151–9 (2000).

668. Alamed, J., Wilcock, D. M., Diamond, D. M., Gordon, M. N. & Morgan, D. Two-day radial-arm water maze learning and memory task; robust resolution of amyloid-related memory deficits in transgenic mice. *Nat. Protoc.* **1,** 1671–9 (2006).

669. Clements, K. M. & Wainwright, P. E. Spontaneously hypertensive, Wistar Kyoto and Sprague-Dawley rats differ in performance on a win-stay task and a conditioned cue preference task in the water radial arm maze. *Behav. Brain Res.* **183,** 169–77 (2007).

670. Randall, L. O. & Selitto, J. J. A method for measurement of analgesic activity on inflamed tissue. *Arch. Int. Pharmacodyn. Ther.* **111,** 409–19 (1957).

671. Takesue, E. I., Schaefer, W. & Jukniewicz, E. Modification of the Randall-Selitto analgesic apparatus. *J. Pharm. Pharmacol.* **21,** 788–9 (1969).

672. Anseloni, V. C. Z., Ennis, M. & Lidow, M. S. Optimization of the mechanical nociceptive threshold testing with the Randall–Selitto assay. *J. Neurosci. Methods* **131,** 93–97 (2003).

673. Santos-Nogueira, E., Redondo Castro, E., Mancuso, R. & Navarro, X. Randall-Selitto test: a new approach for the detection of neuropathic pain after spinal cord injury. *J. Neurotrauma* **29,** 898–904 (2012).

674. Thurmond, J. B. Technique for producing and measuring territorial aggression using laboratory mice. *Physiol. Behav.* **14,** 879–81 (1975).

675. Ebert, P. D. & Hyde, J. S. Selection for agonistic begavior in wild female Mus musculus. *Behav. Genet.* **6,** 291–304 (1976).

676. Heinrichs, S. C. & Koob, G. F. Application of experimental stressors in laboratory rodents. *Curr. Protoc. Neurosci.* **Chapter 8,** Unit8.4 (2006).

677. Widholm, J. J. *et al.* Spatial reversal learning in Aroclor 1254-exposed rats: sex-specific deficits in associative ability and inhibitory control. *Toxicol. Appl. Pharmacol.* **174,** 188–98 (2001).

678. Bissonette, G. B. & Powell, E. M. Reversal learning and attentional set-shifting in mice. *Neuropharmacology* **62,** 1168–74 (2012).

679. Watson, D. J. & Stanton, M. E. Medial prefrontal administration of MK-801 impairs T-maze discrimination reversal learning in weanling rats. *Behav. Brain Res.* **205,** 57–66 (2009).

680. Dong, Z. *et al.* Hippocampal long-term depression mediates spatial reversal learning in the Morris water maze. *Neuropharmacology* **64,** 65–73 (2013).

681. Pubols, B. H. Successive discrimination reversal learning in the white rat: a comparison of two procedures. *J. Comp. Physiol. Psychol.* **50,** 319–22 (1957).

682. Winter, C. A. & Flataker, L. The effect of antihistaminic drugs upon the performance of trained rats. *J. Pharmacol. Exp. Ther.* **101,** 156–62 (1951).

683. Vogel, W. H. Physiological disposition of 5-methoxytryptamine and the rope climbing performance of rats. *Psychopharmacologia* **15,** 88–95 (1969).

684. Axelrod, S., Kankolenski, P. F., Small, S. M., Corvin, A. & Sanders, B. E. Effects on rat rope-climbing of injection with blood plasma fractions from schizophrenics and normals. *J. Psychiatr. Res.* **8,** 1–12 (1970).

685. Fowler, S. C. & Senyuz, L. Effects of haloperidol on a run-climb-run behavioral task: distance climbed does not alter within-session decrements. *Behav. Neurosci.* **107,** 651–61 (1993).

686. Thallmair, M. *et al.* Neurite growth inhibitors restrict plasticity and functional recovery following corticospinal tract lesions. *Nat. Neurosci.* **1,** 124–31 (1998).

687. Wang, H., Spinner, R. J. & Windebank, A. J. Quantitative evaluation of movement and strength of the upper limb after transection of the C-7 nerve: is it possible in an animal model? *J. Neurosurg. Spine* **10,** 102–10 (2009).

688. Dunham, N. W. & Miya, T. S. A note on a simple apparatus for detecting neurological deficit in rats and mice. *J. Am. Pharm. Assoc. Am. Pharm. Assoc. (Baltim).* **46,** 208–9 (1957).

689. Watzman, N., Barry, H., Kinnard, W. J. & Buckley, J. P. Influence of certain parameters on the performance of mice on the rotarod. *Arch. Int. Pharmacodyn. Ther.* **169,** 362–74 (1967).

690. Jones, B. J. & Roberts, D. J. The quantiative measurement of motor inco-ordination in naive mice using an acelerating rotarod. *J. Pharm. Pharmacol.* **20,** 302–4 (1968).

691. Jones, B. J. & Roberts, D. J. A rotarod suitable for quantitative measurements of motor incoordination in naive mice. *Naunyn. Schmiedebergs. Arch. Exp. Pathol. Pharmakol.* **259,** 211 (1968).

692. Bogo, V., Hill, T. A. & Young, R. W. Comparison of accelerod and rotarod sensitivity in detecting ethanol- and acrylamide-induced performance decrement in rats: review of experimental considerations of rotating rod systems. *Neurotoxicology* **2,** 765–87 (1981).

693. Hamm, R. J., Pike, B. R., O'Dell, D. M., Lyeth, B. G. & Jenkins, L. W. The rotarod test: an evaluation of its effectiveness in assessing motor deficits following traumatic brain injury. *J. Neurotrauma* **11,** 187–96 (1994).

694. Rozas, G. & Labandeira García, J. L. Drug-free evaluation of rat models of parkinsonism and nigral grafts using a new automated rotarod test. *Brain Res.* **749,** 188–99 (1997).

695. Pritchett, K. & Mulder, G. B. The rotarod. *Contemp. Top. Lab. Anim. Sci.* **42,** 49 (2003).

696. O'Connor, C., Heath, D. L., Cernak, I., Nimmo, A. J. & Vink, R. Effects of daily versus weekly testing and pre-training on the assessment of neurologic impairment following diffuse traumatic brain injury in rats. *J. Neurotrauma* **20,** 985–93 (2003).

697. Rustay, N. R., Wahlsten, D. & Crabbe, J. C. Influence of task parameters on rotarod performance and sensitivity to ethanol in mice. *Behav. Brain Res.* **141,** 237–49 (2003).

698. Monville, C., Torres, E. M. & Dunnett, S. B. Comparison of incremental and accelerating protocols of the rotarod test for the assessment of motor deficits in the 6-OHDA model. *J. Neurosci. Methods* **158,** 219–23 (2006).

699. Whishaw, I. Q., Li, K., Whishaw, P. A., Gorny, B. & Metz, G. A. Use of rotorod as a method for the qualitative analysis of walking in rat. *J. Vis. Exp.* 1–4 (2008).

700. Bohlen, M., Cameron, A., Metten, P., Crabbe, J. C. & Wahlsten, D. Calibration of rotational acceleration for the rotarod test of rodent motor coordination. *J. Neurosci. Methods* **178,** 10–4 (2009).

701. Ohlsson, A. L. & Johansson, B. B. Environment influences functional outcome of cerebral infarction in rats. *Stroke.* **26,** 644–9 (1995).

702. Grabowski, M., Sørensen, J. C., Mattsson, B., Zimmer, J. & Johansson, B. B. Influence of an enriched environment and cortical grafting on functional outcome in brain infarcts of adult rats. *Exp. Neurol.* **133,** 96–102 (1995).

703. Mattiasson, G. J. *et al.* The rotating pole test: evaluation of its effectiveness in assessing functional motor deficits following experimental head injury in the rat. *J. Neurosci. Methods* **95,** 75–82 (2000).

704. Ruscher, K., Kuric, E. & Wieloch, T. Levodopa treatment improves functional recovery after experimental stroke. *Stroke.* **43,** 507–13 (2012).

705. Denenberg, V. H., Talgo, N. W., Waters, N. S. & Kenner, G. H. A computer-aided procedure for measuring swim rotation. *Physiol. Behav.* **47,** 1023–5 (1990).

706. Schmidt, S. L., Filgueiras, C. C. & Krahe, T. E. Effects of sex and laterality on the rotatory swimming behavior of normal mice. *Physiol. Behav.* **65,** 607–16 (1999).

707. Gasne, M., Millot, J. L., Brand, G. & Math, F. Intercorrelations among tests of lateralisation in the BALB/c mouse. *Laterality* **6,** 89–96 (2001).

708. Manhães, A. C., Abreu-Villaça, Y., Schmidt, S. L. & Filgueiras, C. C. Neonatal transection of the corpus callosum affects rotational side preference in adult Swiss mice. *Neurosci. Lett.* **415,** 159–63 (2007).

709. Ungerstedt, U. & Arbuthnott, G. W. Quantitative recording of rotational behavior in rats after 6-hydroxy-dopamine lesions of the nigrostriatal dopamine system. *Brain Res.* **24,** 485–93 (1970).

710. Greenstein, S. & Glick, S. D. Improved automated apparatus for recording rotation (circling behavior) in rats or mice. *Pharmacol. Biochem. Behav.* **3,** 507–10 (1975).

711. Schwarz, R. D., Stein, J. W. & Bernard, P. Rotometer for recording rotation in chemically or electrically stimulated rats. *Physiol. Behav.* **20,** 351–4 (1978).

712. Jerussi, T. P. A simple, inexpensive rotometer for automatically recording the dynamics of circling behavior. *Pharmacol. Biochem. Behav.* **16,** 353–7 (1982).

713. Torello, M. W., Czekajewski, J., Potter, E. A., Kober, K. J. & Fung, Y. K. An automated method for measurement of circling behavior in the mouse. *Pharmacol. Biochem. Behav.* **19,** 13–7 (1983).

714. Robinson, T. E. & Becker, J. B. The rotational behavior model: asymmetry in the effects of unilateral 6-OHDA lesions of the substantia nigra in rats. *Brain Res.* **264,** 127–31 (1983).

715. Kehinde, L. O., Buraimoh-Igbo, L. A., Ude, O. U. & Makanjuola, R. O. Electronic rotameter for quantitative evaluation of rotational behaviour in rats after unilateral lesions of the nigrostriatal dopamine system. *Med. Biol. Eng. Comput.* **22,** 361–6 (1984).

716. Etemadzadeh, E., Koskinen, L. & Kaakkola, S. Computerized rotometer apparatus for recording circling behavior. *Methods Find. Exp. Clin. Pharmacol.* **11,** 399–407 (1989).

717. D'Anna, L. *et al.* A novel, fully automated rotometer for the study of turning behavior. Comparison of results under different experimental conditions. *Acta Physiol. Pharmacol. Latinoam.* **39,** 255–68 (1989).

718. Pons, S., López, J. A., Ramis, C., Planas, B. & Rial, R. A new precise microcomputer based rotometer. *J. Neurosci. Methods* **32,** 155–8 (1990).

719. Hudson, J. L., Levin, D. R. & Hoffer, B. J. A 16-channel automated rotometer system for reliable measurement of turning behavior in 6-hydroxydopamine lesioned and transplanted rats. *Cell Transplant.* **2,** 507–14 (1993).

720. Heredia-López, F. J., Bata-García, J. L., Alvarez-Cervera, F. J. & Góngora-Alfaro, J. L. A novel rotometer based on a RISC microcontroller. *Behav. Res. Methods. Instrum. Comput.* **34,** 399–407 (2002).

721. Marin, C., Rodriguez-Oroz, M. C. & Obeso, J. A. Motor complications in Parkinson's disease and the clinical significance of rotational behavior in the rat: have we wasted our time? *Exp. Neurol.* **197,** 269–74 (2006).

722. Löscher, W. Abnormal circling behavior in rat mutants and its relevance to model specific brain dysfunctions. *Neurosci. Biobehav. Rev.* **34,** 31–49 (2010).

723. Stahel, P. F. *et al.* Experimental closed head injury: analysis of neurological outcome, blood-brain barrier dysfunction, intracranial neutrophil infiltration, and neuronal cell death in mice deficient in genes for pro-inflammatory cytokines. *J. Cereb. Blood Flow Metab.* **20,** 369–80 (2000).

724. Weeks, J. R. Experimental morphine addiction: method for automatic intravenous injections in unrestrained rats. *Science* **138,** 143–4 (1962).

725. Pickens, R. & Thompson, T. Cocaine-reinforced behavior in rats: effects of reinforcement magnitude and fixed-ratio size. *J. Pharmacol. Exp. Ther.* **161,** 122–9 (1968).

726. Schuster, C. R. & Thompson, T. Self administration of and behavioral dependence on drugs. *Annu. Rev. Pharmacol.* **9,** 483–502 (1969).

727. Blakesley, B. C., Dinneen, L. C., Elliott, R. D. & Francis, D. L. Intravenous self-administration of heroin in the rat: experimental technique and computer analysis. *Br. J. Pharmacol.* **45,** 181P–182P (1972).

728. Goeders, N. E. & Smith, J. E. Intracranial self-administration methodologies. *Neurosci. Biobehav. Rev.* **11,** 319–29 (1987).

729. Richardson, N. R. & Roberts, D. C. Progressive ratio schedules in drug self-administration studies in rats: a method to evaluate reinforcing efficacy. *J. Neurosci. Methods* **66,** 1–11 (1996).

730. Grahame, N. J. & Cunningham, C. L. Intravenous self-administration of ethanol in mice. *Curr. Protoc. Neurosci.* **Chapter 9,** Unit 9.11 (2002).

731. Samson, H. H. & Czachowski, C. L. Behavioral measures of alcohol self-administration and intake control: rodent models. *Int. Rev. Neurobiol.* **54,** 107–43 (2003).

732. Thomsen, M. & Caine, S. B. Chronic intravenous drug self-administration in rats and mice. *Curr. Protoc. Neurosci.* **Chapter 9,** Unit 9.20 (2005).

733. Thomsen, M. & Caine, S. B. Intravenous drug self-administration in mice: practical considerations. *Behav. Genet.* **37,** 101–18 (2007).

734. Panlilio, L. V & Goldberg, S. R. Self-administration of drugs in animals and humans as a model and an investigative tool. *Addiction* **102,** 1863–70 (2007).

735. June, H. L. & Gilpin, N. W. Operant self-administration models for testing the neuropharmacological basis of ethanol consumption in rats. *Curr. Protoc. Neurosci.* **Chapter 9,** Unit 9.12.1–26 (2010).

736. Maier, E. Y., Ma, S. T., Ahrens, A., Schallert, T. J. & Duvauchelle, C. L. Assessment of ultrasonic vocalizations during drug self-administration in rats. *J. Vis. Exp.* 1–4 (2010). doi:10.3791/2041

737. O'Connor, E. C., Chapman, K., Butler, P. & Mead, A. N. The predictive validity of the rat self-administration model for abuse liability. *Neurosci. Biobehav. Rev.* **35,** 912–38 (2011).

738. Kmiotek, E. K., Baimel, C. & Gill, K. J. Methods for intravenous self administration in a mouse model. *J. Vis. Exp.* e3739 (2012).

739. Trueman, R. C., Brooks, S. P. & Dunnett, S. B. Implicit learning in a serial choice visual discrimination task in the operant 9-hole box by intact and striatal lesioned mice. *Behav. Brain Res.* **159,** 313–322 (2005).

740. Jay, J. R. & Dunnett, S. B. An operant serial implicit learning task (SILT) in rats: task acquisition, performance and the effects of striatal lesions. *J. Neurosci. Methods* **163,** 235–44 (2007).

741. Pinel, J. P. & Treit, D. Burying as a defensive response in rats. *J. Comp. Physiol. Psychol.* **92,** 708–712 (1978).

742. Treit, D., Pinel, J. P. & Fibiger, H. C. Conditioned defensive burying: a new paradigm for the study of anxiolytic agents. *Pharmacol. Biochem. Behav.* **15,** 619–26 (1981).

743. Craft, R. M., Howard, J. L. & Pollard, G. T. Conditioned defensive burying as a model for identifying anxiolytics. *Pharmacol. Biochem. Behav.* **30,** 775–80 (1988).

744. Treit, D. A comparison of anxiolytic and nonanxiolytic agents in the shock-probe/burying test for anxiolytics. *Pharmacol. Biochem. Behav.* **36,** 203–5 (1990).

745. De Boer, S. F. & Koolhaas, J. M. Defensive burying in rodents: ethology, neurobiology and psychopharmacology. *Eur. J. Pharmacol.* **463,** 145–161 (2003).

746. Denny, M. R. & Thomas, J. O. Avoidance learning and relearning as a function of shuttlebox dimensions. *Science* **132,** 620–1 (1960).

747. Cotton, M. M. Overshadowing in a two-way shuttlebox. *Psychol. Rep.* **53,** 367–73 (1983).

748. Bozarth, M. A. A computer approach to measuring shuttle box activity and conditioned place preference. *Brain Res. Bull.* **11,** 751–3 (1983).

749. Wadenberg, M. L. *et al.* A novel computer-controlled conditioned avoidance apparatus for rats. *J. Pharmacol. Toxicol. Methods* **38,** 211–5 (1997).

750. Savonenko, A. V, Brush, F. R. & Zieliński, K. How do rats cope with the two-way escape problem in a homogeneous shuttle box? *Acta Neurobiol. Exp. (Wars).* **59,** 145–57 (1999).

751. Wadenberg, M. L. & Hicks, P. B. The conditioned avoidance response test re-evaluated: is it a sensitive test for the detection of potentially atypical antipsychotics? *Neurosci. Biobehav. Rev.* **23,** 851–62 (1999).

752. Clark, M. G., Vasilevsky, S. & Myers, T. M. Air and shock two-way shuttlebox avoidance in C57BL/6J and 129X1/SvJ mice. *Physiol. Behav.* **78,** 117–23 (2003).

753. Myers, T. M., Cohn, S. I. & Clark, M. G. Acquisition and performance of two-way shuttlebox avoidance: Effects of aversive air intensity. *Learn. Motiv.* **36,** 312–321 (2005).

754. Whishaw, I. Q. & Pellis, S. M. The structure of skilled forelimb reaching in the rat: a proximally driven movement with a single distal rotatory component. *Behav. Brain Res.* **41,** 49–59 (1990).

755. Whishaw, I. Q., Gorny, B., Foroud, A. & Kleim, J. A. Long–Evans and Sprague–Dawley rats have similar skilled reaching success and limb representations in motor cortex but different movements: some cautionary insights into the selection of rat strains for neurobiological motor research. *Behav. Brain Res.* **145,** 221–232 (2003).

756. Alaverdashvili, M., Leblond, H., Rossignol, S. & Whishaw, I. Q. Cineradiographic (video X-ray) analysis of skilled reaching in a single pellet reaching task provides insight into relative contribution of body, head, oral, and forelimb movement in rats. *Behav. Brain Res.* **192,** 232–47 (2008).

757. Whishaw, I. Q., Whishaw, P. & Gorny, B. The structure of skilled forelimb reaching in the rat: a movement rating scale. *J. Vis. Exp.* (2008).

758. Klein, A., Sacrey, L.-A. R., Whishaw, I. Q. & Dunnett, S. B. The use of rodent skilled reaching as a translational model for investigating brain damage and disease. *Neurosci. Biobehav. Rev.* **36,** 1030–42 (2012).

759. Klein, A. & Dunnett, S. B. Analysis of skilled forelimb movement in rats: the single pellet reaching test and staircase test. *Curr. Protoc. Neurosci.* **Chapter 8,** Unit8.28 (2012).

760. Millan, M. J. & Bales, K. L. Towards improved animal models for evaluating social cognition and its disruption in schizophrenia: The CNTRICS initiative. *Neurosci. Biobehav. Rev.* **37,** 2166–80 (2013).

761. Engelmann, M., Hädicke, J. & Noack, J. Testing declarative memory in laboratory rats and mice using the nonconditioned social discrimination procedure. *Nat. Protoc.* **6,** 1152–62 (2011).

762. Ferguson, J. N., Young, L. J. & Insel, T. R. The neuroendocrine basis of social recognition. *Front. Neuroendocrinol.* **23,** 200–24 (2002).

763. Engelmann, M., Wotjak, C. T. & Landgraf, R. Social discrimination procedure: an alternative method to investigate juvenile recognition abilities in rats. *Physiol. Behav.* **58,** 315–21 (1995).

764. Tejada, L. D. & Rissman, E. F. Sex differences in social investigation: effects of androgen receptors, hormones and test partner. *J. Neuroendocrinol.* **24,** 1144–53 (2012).

765. Winslow, J. T. & Camacho, F. Cholinergic modulation of a decrement in social investigation following repeated contacts between mice. *Psychopharmacology (Berl).* **121,** 164–72 (1995).

766. Crowder, W. F. & Hutto, C. W. Operant place conditioning measures examined using two nondrug reinforcers. *Pharmacol. Biochem. Behav.* **41,** 817–24 (1992).

767. Calcagnetti, D. J. & Schechter, M. D. Place conditioning reveals the rewarding aspect of social interaction in juvenile rats. *Physiol. Behav.* **51,** 667–72 (1992).

768. Van den Berg, C. L. *et al.* Isolation changes the incentive value of sucrose and social behaviour in juvenile and adult rats. *Behav. Brain Res.* **106,** 133–42 (1999).

769. Schwabe, K., Klein, S. & Koch, M. Behavioural effects of neonatal lesions of the medial prefrontal cortex and subchronic pubertal treatment with phencyclidine of adult rats. *Behav. Brain Res.* **168,** 150–60 (2006).

770. Trezza, V., Damsteegt, R., Achterberg, E. J. M. & Vanderschuren, L. J. M. J. Nucleus accumbens μ-opioid receptors mediate social reward. *J. Neurosci.* **31,** 6362–70 (2011).

771. Achterberg, E. J. M., Trezza, V. & Vanderschuren, L. J. M. J. β-Adrenoreceptor stimulation mediates reconsolidation of social reward-related memories. *PLoS One* **7,** e39639 (2012).

772. Niesink, R. J. & van Ree, J. M. Short-term isolation increases social interactions of male rats: a parametric analysis. *Physiol. Behav.* **29,** 819–25 (1982).

773. File, S. E. & Seth, P. A review of 25 years of the social interaction test. *Eur. J. Pharmacol.* **463,** 35–53 (2003).

774. File, S. E. & Hyde, J. R. Can social interaction be used to measure anxiety? *Br. J. Pharmacol.* **62,** 19–24 (1978).

775. Poulin, J.-F., Bérubé, P., Laforest, S. & Drolet, G. Enkephalin knockdown in the central amygdala nucleus reduces unconditioned fear and anxiety. *Eur. J. Neurosci.* **37,** 1357–67 (2013).

776. Arsenault, D. *et al.* PAK inactivation impairs social recognition in 3xTg-AD Mice without increasing brain deposition of tau and Aβ. *J. Neurosci.* **33,** 10729–40 (2013).

777. Thor, D. H. & Holloway, W. R. Social memory of the male laboratory rat. *J. Comp. Physiol. Psychol.* **96,** 1000–1006 (1982).

778. Galef, B. G. & Wigmore, S. W. Transfer of information concerning distant foods: A laboratory investigation of the "information-centre" hypothesis. *Anim. Behav.* **31,** 748–758 (1983).

779. Galef, B. G., Wigmore, S. W. & Kennett, D. J. A failure to find socially mediated taste aversion learning in Norway rats (R. norvegicus). *J. Comp. Psychol.* **97,** 358–63 (1983).

780. Galef, B. G. Utilization by Norway rats (R. norvegicus) of multiple messages concerning distant foods. *J. Comp. Psychol.* **97,** 364–371 (1983).

781. Wrenn, C. C., Harris, A. P., Saavedra, M. C. & Crawley, J. N. Social transmission of food preference in mice: Methodology and application to galanin-overexpressing transgenic mice. *Behav. Neurosci.* **117,** 21–31 (2003).

782. Galef, B. G. & Whiskin, E. E. Socially transmitted food preferences can be used to study long-term memory in rats. *Learn. Behav.* **31,** 160–4 (2003).

783. Gold, P. E., Countryman, R. A., Dukala, D. & Chang, Q. Acetylcholine release in the hippocampus and prelimbic cortex during acquisition of a socially transmitted food preference. *Neurobiol. Learn. Mem.* **96,** 498–503 (2011).

784. Molinengo, L. & Ricci-Gamalero, S. The “staircase maze” and the “simple staircase” in the analysis of the psychopharmacological action of CNS depressants. *Pharmacology* **4,** 169–78 (1970).

785. Molinengo, L. The action of scopolamine on retrieval and memory storage in rats evaluated in the staircase maze. *Behav. Neural Biol.* **59,** 18–24 (1993).

786. Molinengo, L., Orsetti, M., Pastorello, B., Scordo, I. & Ghi, P. The action of arecoline on retrieval and memory storage evaluated in the staircase maze. *Neurobiol. Learn. Mem.* **63,** 167–73 (1995).

787. Molinengo, L. & Ghi, P. Behavioral and neurochemical effects induced by subchronic l-deprenyl administration. *Pharmacol. Biochem. Behav.* **58,** 649–55 (1997).

788. Montoya, C. P., Campbell-Hope, L. J., Pemberton, K. D. & Dunnett, S. B. The “staircase test”: a measure of independent forelimb reaching and grasping abilities in rats. *J. Neurosci. Methods* **36,** 219–28 (1991).

789. Whishaw, I. Q., Woodward, N. C., Miklyaeva, E. & Pellis, S. M. Analysis of limb use by control rats and unilateral DA-depleted rats in the Montoya staircase test: movements, impairments and compensatory strategies. *Behav. Brain Res.* **89,** 167–77 (1997).

790. Nikkhah, G., Rosenthal, C., Hedrich, H. J. & Samii, M. Differences in acquisition and full performance in skilled forelimb use as measured by the “staircase test” in five rat strains. *Behav. Brain Res.* **92,** 85–95 (1998).

791. Baird, A. L., Meldrum, A. & Dunnett, S. B. The staircase test of skilled reaching in mice. *Brain Res. Bull.* **54,** 243–50 (2001).

792. Samsam, T. E., Gadrinab, L. G. & Bushnell, P. J. Toxicological evaluation of the staircase test for assessing fine motor movements. *Neurotoxicol. Teratol.* **26,** 113–20 (2003).

793. Kloth, V., Klein, A., Loettrich, D. & Nikkhah, G. Colour-coded pellets increase the sensitivity of the staircase test to differentiate skilled forelimb performances of control and 6-hydroxydopamine lesioned rats. *Brain Res. Bull.* **70,** 68–80 (2006).

794. Pagnussat, A. D. S., Michaelsen, S. M., Achaval, M. & Netto, C. A. Skilled forelimb reaching in Wistar rats: evaluation by means of Montoya staircase test. *J. Neurosci. Methods* **177,** 115–21 (2009).

795. Boltze, J. *et al.* The stairway: a novel behavioral test detecting sensomotoric stroke deficits in rats. *Artif. Organs* **30,** 756–63 (2006).

796. Olsson, M., Nikkhah, G., Bentlage, C. & Björklund, a. Forelimb akinesia in the rat Parkinson model: differential effects of dopamine agonists and nigral transplants as assessed by a new stepping test. *J. Neurosci.* **15,** 3863–75 (1995).

797. Blume, S. R., Cass, D. K. & Tseng, K. Y. Stepping test in mice: a reliable approach in determining forelimb akinesia in MPTP-induced Parkinsonism. *Exp. Neurol.* **219,** 208–11 (2009).

798. Eagle, D. M. & Robbins, T. W. Inhibitory control in rats performing a stop-signal reaction-time task: effects of lesions of the medial striatum and d-amphetamine. *Behav. Neurosci.* **117,** 1302–17 (2003).

799. Eagle, D. M., Bari, A. & Robbins, T. W. The neuropsychopharmacology of action inhibition: cross-species translation of the stop-signal and go/no-go tasks. *Psychopharmacology (Berl).* **199,** 439–56 (2008).

800. Deacon, R. M. J., Penny, C. & Rawlins, J. N. P. Effects of medial prefrontal cortex cytotoxic lesions in mice. *Behav. Brain Res.* **139,** 139–55 (2003).

801. Deacon, R. M. J. The successive alleys test of anxiety in mice and rats. *J. Vis. Exp.* 1–7 (2013). doi:10.3791/2705

802. Campbell, B. A. Absolute and relative sucrose preference thresholds for hungry and satiated rats. *J. Comp. Physiol. Psychol.* **51,** 795–800 (1958).

803. Levine, R. Genetic relationships, choice models, and sucrose preference behaviour in mice. *Nature* **215,** 668–9 (1967).

804. Pothion, S., Bizot, J.-C., Trovero, F. & Belzung, C. Strain differences in sucrose preference and in the consequences of unpredictable chronic mild stress. *Behav. Brain Res.* **155,** 135–46 (2004).

805. Crawford, C. A., Der-Ghazarian, T., Britt, C. E., Varela, F. A. & Kozanian, O. O. Novelty-induced conditioned place preference, sucrose preference, and elevated plus maze behavior in adult rats after repeated exposure to methylphenidate during the preweanling period. *Behav. Brain Res.* **246C,** 29–35 (2013).

806. Bushnell, P. J., Kelly, K. L. & Crofton, K. M. Effects of toluene inhalation on detection of auditory signals in rats. *Neurotoxicol. Teratol.* **16,** 149–60 (1994).

807. McGaughy, J. & Sarter, M. Behavioral vigilance in rats: task validation and effects of age, amphetamine, and benzodiazepine receptor ligands. *Psychopharmacology (Berl).* **117,** 340–57 (1995).

808. Burk, J. A. Introduction of a retention interval in a sustained attention task in rats: effects of a visual distracter and increasing the inter-trial interval. *Behav. Processes* **67,** 521–31 (2004).

809. Bushnell, P. J. & Strupp, B. J. in (Buccafusco, J.) (CRC Press, 2009).

810. St Peters, M., Cherian, A. K., Bradshaw, M. & Sarter, M. Sustained attention in mice: expanding the translational utility of the SAT by incorporating the Michigan Controlled Access Response Port (MICARP). *Behav. Brain Res.* **225,** 574–83 (2011).

811. Schapiro, S., Salas, M. & Vukovich, K. Hormonal effects on ontogeny of swimming ability in the rat: assessment of central nervous system development. *Science* **168,** 147–50 (1970).

812. Gruner, J. A. & Altman, J. Swimming in the rat: analysis of locomotor performance in comparison to stepping. *Exp. Brain Res.* **40,** 374–82 (1980).

813. Donnan, G. A., Willis, G. L., Kaczmarczyk, S. J. & Rowe, P. Motor function in the 1-methyl-4-phenyl-1,2,3,6-tetrahydropyridine-treated mouse. *J. Neurol. Sci.* **77,** 185–91 (1987).

814. Haobam, R., Sindhu, K. M., Chandra, G. & Mohanakumar, K. P. Swim-test as a function of motor impairment in MPTP model of Parkinson's disease: a comparative study in two mouse strains. *Behav. Brain Res.* **163,** 159–67 (2005).

815. Liebscher, T. *et al.* Nogo-A antibody improves regeneration and locomotion of spinal cord-injured rats. *Ann. Neurol.* **58,** 706–19 (2005).

816. Magnuson, D. S. K. *et al.* Swimming as a model of task-specific locomotor retraining after spinal cord injury in the rat. *Neurorehabil. Neural Repair* **23,** 535–45 (2009).

817. Lockey, A. J., Kavaliers, M. & Ossenkopp, K.-P. Lipopolysaccharide reduces tactile startle response magnitude but not prepulse inhibition in rats: a dose-response examination. *Pharmacol. Biochem. Behav.* **93,** 47–53 (2009).

818. Geyer, M. A. *et al.* The effects of lysergic acid diethylamide and mescaline-derived hallucinogens on sensory-integrative function: tactile startle. *J. Pharmacol. Exp. Ther.* **207,** 837–47 (1978).

819. Geyer, M. A., Warbritton, J. D., Menkes, D. B., Zook, J. a & Mandell, a J. Opposite effects of intraventricular serotonin and bufotenin on rat startle responses. *Pharmacol. Biochem. Behav.* **3,** 687–91 (1975).

820. Steru, L., Chermat, R., Thierry, B. & Simon, P. The tail suspension test: a new method for screening antidepressants in mice. *Psychopharmacology (Berl).* **85,** 367–70 (1985).

821. Thierry, B., Stéru, L., Simon, P. & Porsolt, R. D. The tail suspension test: ethical considerations. *Psychopharmacology (Berl).* **90,** 284–5 (1986).

822. Chermat, R., Thierry, B., Mico, J. A., Steru, L. & Simon, P. Adaptation of the tail suspension test to the rat. *J. Pharmacol.* **17,** 348–50 (1986).

823. Steru, L. *et al.* The automated Tail Suspension Test: a computerized device which differentiates psychotropic drugs. *Prog. Neuropsychopharmacol. Biol. Psychiatry* **11,** 659–71 (1987).

824. Mayorga, A. J. & Lucki, I. Limitations on the use of the C57BL/6 mouse in the tail suspension test. *Psychopharmacology (Berl).* **155,** 110–112 (2001).

825. Ripoll, N., David, D. J. P., Dailly, E., Hascoët, M. & Bourin, M. Antidepressant-like effects in various mice strains in the tail suspension test. *Behav. Brain Res.* **143,** 193–200 (2003).

826. Cryan, J. F., Mombereau, C. & Vassout, A. The tail suspension test as a model for assessing antidepressant activity: review of pharmacological and genetic studies in mice. *Neurosci. Biobehav. Rev.* **29,** 571–625 (2005).

827. Can, A. *et al.* The tail suspension test. *J. Vis. Exp.* e3769 (2012).

828. D'Amour, F. E. & Smith, D. L. A method for determining loss of pain sensation. *J. Pharmacol. Exp. Ther.* **72,** 74–9 (1941).

829. Hillman, G. R. A digital tail-flick apparatus. *Med. Biol. Eng.* **13,** 597–8 (1975).

830. Ness, T. J., Jones, S. L. & Gebhart, G. F. Contribution of the site of heating to variability in the latency of the rat tail flick reflex. *Brain Res.* **426,** 169–72 (1987).

831. Berge, O. G., Garcia-Cabrera, I. & Hole, K. Response latencies in the tail-flick test depend on tail skin temperature. *Neurosci. Lett.* **86,** 284–8 (1988).

832. Hole, K. *et al.* The tail-flick test needs to be improved. *Pain* **43,** 391–3 (1990).

833. Han, J. S. & Ren, M. F. The importance of monitoring tail-skin temperature in measuring tail-flick latency. *Pain* **46,** 117 (1991).

834. Grant, G. J. *et al.* Assessing local anesthetic effect using the mouse tail flick test. *J. Pharmacol. Toxicol. Methods* **29,** 223–6 (1993).

835. Janssen, P. A., Niemegeers, C. J. & Dony, J. G. The inhibitory effect of fentanyl and other morphine-like analgesics on the warm water induced tail withdrawl reflex in rats. *Arzneimittelforschung.* **13,** 502–7 (1963).

836. Luttinger, D. Determination of antinociceptive efficacy of drugs in mice using different water temperatures in a tail-immersion test. *J. Pharmacol. Methods* **13,** 351–7 (1985).

837. Ramabadran, K., Bansinath, M., Turndorf, H. & Puig, M. M. Tail immersion test for the evaluation of a nociceptive reaction in mice. Methodological considerations. *J. Pharmacol. Methods* **21,** 21–31 (1989).

838. Kotlinska, J. H. *et al.* Antinociceptive effects of two deltorphins analogs in the tail-immersion test in rats. *Peptides* **39,** 103–10 (2013).

839. Tarlov, I. M. & Klinger, H. Spinal cord compression studies. II. Time limits for recovery after acute compression in dogs. *AMA. Arch. Neurol. Psychiatry* **71,** 271–90 (1954).

840. Wrathall, J. R., Pettegrew, R. K. & Harvey, F. Spinal cord contusion in the rat: production of graded, reproducible, injury groups. *Exp. Neurol.* **88,** 108–22 (1985).

841. Guth, L., Zhang, Z. & Roberts, E. Key role for pregnenolone in combination therapy that promotes recovery after spinal cord injury. *Proc. Natl. Acad. Sci. U. S. A.* **91,** 12308–12 (1994).

842. Guth, L., Zhang, Z., DiProspero, N. A., Joubin, K. & Fitch, M. T. Spinal cord injury in the rat: treatment with bacterial lipopolysaccharide and indomethacin enhances cellular repair and locomotor function. *Exp. Neurol.* **126,** 76–87 (1994).

843. Popovich, P. G. *et al.* A reassessment of a classic neuroprotective combination therapy for spinal cord injured rats: LPS/pregnenolone/indomethacin. *Exp. Neurol.* **233,** 677–85 (2012).

844. Brenes, R. A. *et al.* Toward a mouse model of hind limb ischemia to test therapeutic angiogenesis. *J. Vasc. Surg.* **56,** 1669–79; discussion 1679 (2012).

845. Nadler, J. J. *et al.* Automated apparatus for quantitation of social approach behaviors in mice. *Genes. Brain. Behav.* **3,** 303–14 (2004).

846. Moy, S. S. *et al.* Sociability and preference for social novelty in five inbred strains: an approach to assess autistic-like behavior in mice. *Genes. Brain. Behav.* **3,** 287–302 (2004).

847. Pearson, B. L., Defensor, E. B., Blanchard, D. C. & Blanchard, R. J. C57BL/6J mice fail to exhibit preference for social novelty in the three-chamber apparatus. *Behav. Brain Res.* **213,** 189–94 (2010).

848. Yang, M., Silverman, J. L. & Crawley, J. N. Automated three-chambered social approach task for mice. *Curr. Protoc. Neurosci.* **Chapter 8,** Unit 8.26 (2011).

849. Kaidanovich-Beilin, O., Lipina, T., Vukobradovic, I., Roder, J. & Woodgett, J. R. Assessment of social interaction behaviors. *J. Vis. Exp.* 1–6 (2011).

850. Furuya, Y., Yamamoto, T., Yatsugi, S. & Ueki, S. A new method for studying working memory by using the three-panel runway apparatus in rats. *Jpn. J. Pharmacol.* **46,** 183–8 (1988).

851. Mutlu, O., Ulak, G., Çelikyurt, I. K., Akar, F. Y. & Erden, F. Effects of Citalopram on Cognitive Performance in Passive Avoidance, Elevated Plus-Maze and Three-Panel Runway Tasks in Naïve Rats. *Chin. J. Physiol.* **54,** 36–46 (2011).

852. Dennis, W. Spontaneous alternation in rats as an indicator of the persistence of stimulus effects. *J. Comp. Psychol.* **28,** 305–312 (1939).

853. Tolman, E. C., Ritchie, B. F. & Kalish, D. Studies in spatial learning. II. Place learning versus response learning. *J. Exp. Psychol.* **36,** 221–229 (1946).

854. Rodriguez, M., Gomez, C., Alonso, J. & Afonso, D. Laterality, alternation, and perseveration relationships on the T-maze test. *Behav. Neurosci.* **106,** 974–80 (1992).

855. Rodriguez, M. & Afonso, D. Ontogeny of T-maze behavioral lateralization in rats. *Physiol. Behav.* **54,** 91–4 (1993).

856. Wenk, G. L. Assessment of spatial memory using the T maze. *Curr. Protoc. Neurosci.* **Chapter 8,** Unit 8.5B (2001).

857. Andrade, C., Alwarshetty, M., Sudha, S. & Suresh Chandra, J. Effect of innate direction bias on T-maze learning in rats: implications for research. *J. Neurosci. Methods* **110,** 31–5 (2001).

858. Dudchenko, P. A. How do animals actually solve the T maze? *Behav. Neurosci.* **115,** 850–860 (2001).

859. Deacon, R. M. J. Appetitive position discrimination in the T-maze. *Nat. Protoc.* **1,** 13–5 (2006).

860. Szelest, I. & Cohen, J. Effects of forced-choice runway variations on rats' T-maze serial pattern learning. *Learn. Behav.* **34,** 202–14 (2006).

861. Locchi, F., Dall'Olio, R., Gandolfi, O. & Rimondini, R. Water T-maze, an improved method to assess spatial working memory in rats: Pharmacological validation. *Neurosci. Lett.* **422,** 213–6 (2007).

862. Moustgaard, A. & Hau, J. Induction of habits in rats by a forced-choice procedure in T-maze and the effect of pre-test free exploration. *Behav. Processes* **82,** 104–7 (2009).

863. Patil, S. S., Sunyer, B., Höger, H. & Lubec, G. Evaluation of spatial memory of C57BL/6J and CD1 mice in the Barnes maze, the Multiple T-maze and in the Morris water maze. *Behav. Brain Res.* **198,** 58–68 (2009).

864. Pistell, P. J. & Ingram, D. K. Development of a water-escape motivated version of the Stone T-maze for mice. *Neuroscience* **166,** 61–72 (2010).

865. Gaskill, B. N., Lucas, J. R., Pajor, E. A. & Garner, J. P. Little and often? Maintaining continued performance in an automated T-maze for mice. *Behav. Processes* **86,** 272–8 (2011).

866. Shoji, H., Hagihara, H., Takao, K., Hattori, S. & Miyakawa, T. T-maze forced alternation and left-right discrimination tasks for assessing working and reference memory in mice. *J. Vis. Exp.* 1–8 (2012).

867. Castro, A. J. The effects of cortical ablations on tongue usage in the rat. *Brain Res.* **45,** 251–3 (1972).

868. Gulyaeva, N. *et al.* Tongue protrusion: a simple test for neurological recovery in rats following focal cerebral ischemia. *J. Neurosci. Methods* **125,** 183–193 (2003).

869. Whishaw, I. Q., O'Connor, W. T. & Dunnett, S. B. The contributions of motor cortex, nigrostriatal dopamine and caudate-putamen to skilled forelimb use in the rat. *Brain* **109 (Pt 5,** 805–43 (1986).

870. Whishaw, I. Q. An endpoint, descriptive, and kinematic comparison of skilled reaching in mice (Mus musculus) with rats (Rattus norvegicus). *Behav. Brain Res.* **78,** 101–11 (1996).

871. Whishaw, I. Q., Piecharka, D. M., Zeeb, F. & Stein, D. G. Unilateral frontal lobe contusion and forelimb function: chronic quantitative and qualitative impairments in reflexive and skilled forelimb movements in rats. *J. Neurotrauma* **21,** 1584–600 (2004).

872. Fouad, K., Vavrek, R. & Cho, S. A TrkB antibody agonist promotes plasticity following cervical spinal cord injury in adult rats. *J. Neurotrauma* 1–35 (2010).

873. Ramos, A., Pereira, E., Martins, G. C., Wehrmeister, T. D. & Izídio, G. S. Integrating the open field, elevated plus maze and light/dark box to assess different types of emotional behaviors in one single trial. *Behav. Brain Res.* **193,** 277–88 (2008).

874. Fraser, L. M. *et al.* Measuring anxiety- and locomotion-related behaviours in mice: a new way of using old tests. *Psychopharmacology (Berl).* **211,** 99–112 (2010).

875. Lindzey, G., Winston, H. & Manosevitz, M. Social dominance in inbred mouse strains. *Nature* **191,** 474–6 (1961).

876. Miczek, K. A. & Barry, H. What does the tube test measure? *Behav. Biol.* **13,** 537–9 (1975).

877. Benton, D., Dalrymple-Alford, J. C. & Brain, P. F. Comparisons of measures of dominance in the laboratory mouse. *Anim. Behav.* **28,** 1274–1279 (1980).

878. Wang, F. *et al.* Bidirectional control of social hierarchy by synaptic efficacy in medial prefrontal cortex. *Science* **334,** 693–7 (2011).

879. Richter, C. P. & Campbell, K. H. Alcohol taste thresholds and concentrations of solution preferred by rats. *Science* **91,** 507–8 (1940).

880. Wilson, A. W., Neill, J. C. & Costall, B. Strain differences in ethanol preference and reinforced behaviour: a comparison of two-bottle choice and operant self-administration paradigms. *Behav. Pharmacol.* **8,** 37–46 (1997).

881. Tordoff, M. G. & Bachmanov, A. A. Influence of test duration on the sensitivity of the two-bottle choice test. *Chem. Senses* **27,** 759–68 (2002).

882. Blizard, D. A., Vandenbergh, D. J., Lionikas, A. & McClearn, G. E. Learning in the 2-bottle alcohol preference test. *Alcohol. Clin. Exp. Res.* **32,** 2041–6 (2008).

883. Green, A. S. & Grahame, N. J. Ethanol drinking in rodents: is free-choice drinking related to the reinforcing effects of ethanol? *Alcohol* **42,** 1–11 (2008).

884. Cao, J., Gautier, N. M. & Li, M. D. CD-1 mice Show Individual Differences in Nicotine Preference in a Modified Two-Bottle Oral Self-Administration Model. *Front. Psychiatry* **3,** 28 (2012).

885. Staubli, U., Thibault, O., DiLorenzo, M. & Lynch, G. Antagonism of NMDA receptors impairs acquisition but not retention of olfactory memory. *Behav. Neurosci.* **103,** 54–60 (1989).

886. Larson, J. & Sieprawska, D. Automated study of simultaneous-cue olfactory discrimination learning in adult mice. *Behav. Neurosci.* **116,** 588–599 (2002).

887. Patel, R. C. & Larson, J. Impaired olfactory discrimination learning and decreased olfactory sensitivity in aged C57Bl/6 mice. *Neurobiol. Aging* **30,** 829–37 (2009).

888. Sewell, G. D. Ultrasonic communication in rodents. *Nature* **227,** 410 (1970).

889. Oswalt, G. L. & Meier, G. W. Olfactory, thermal, and tactual influences on infantile ultrasonic vocalization in rats. *Dev. Psychobiol.* **8,** 129–35 (1975).

890. Ehret, G. Infant rodent ultrasounds -- a gate to the understanding of sound communication. *Behav. Genet.* **35,** 19–29 (2005).

891. Portfors, C. V. Types and functions of ultrasonic vocalizations in laboratory rats and mice. *J. Am. Assoc. Lab. Anim. Sci.* **46,** 28–34 (2007).

892. Brudzynski, S. M. Communication of adult rats by ultrasonic vocalization: biological, sociobiological, and neuroscience approaches. *ILAR J.* **50,** 43–50 (2009).

893. Lahvis, G. P., Alleva, E. & Scattoni, M. L. Translating mouse vocalizations: prosody and frequency modulation. *Genes. Brain. Behav.* **10,** 4–16 (2011).

894. Fischer, J. & Hammerschmidt, K. Ultrasonic vocalizations in mouse models for speech and socio-cognitive disorders: insights into the evolution of vocal communication. *Genes. Brain. Behav.* **10,** 17–27 (2011).

895. Brudzynski, S. M. Ethotransmission: communication of emotional states through ultrasonic vocalization in rats. *Curr. Opin. Neurobiol.* **23,** 310–7 (2013).

896. Desjardins, C., Maruniak, J. A. & Bronson, F. H. Social rank in house mice: differentiation revealed by ultraviolet visualization of urinary marking patterns. *Science* **182,** 939–41 (1973).

897. Drickamer, L. C. Urine marking and social dominance in male house mice (Mus musculus domesticus). *Behav. Processes* **53,** 113–120 (2001).

898. Barth, T. M. & Stanfield, B. B. The recovery of forelimb-placing behavior in rats with neonatal unilateral cortical damage involves the remaining hemisphere. *J. Neurosci.* **10,** 3449–59 (1990).

899. Hua, Y. Behavioral Tests After Intracerebral Hemorrhage in the Rat. *Stroke* **33,** 2478–2484 (2002).

900. Anstrom, K. K., Schallert, T., Woodlee, M. T., Shattuck, A. & Roberts, D. C. S. Repetitive vibrissae-elicited forelimb placing before and immediately after unilateral 6-hydroxydopamine improves outcome in a model of Parkinson's disease. *Behav. Brain Res.* **179,** 183–91 (2007).

901. Khaing, Z. Z. *et al.* Assessing forelimb function after unilateral cervical spinal cord injury: novel forelimb tasks predict lesion severity and recovery. *J. Neurotrauma* **29,** 488–98 (2012).

902. An, X. *et al.* Suppression of experimental autoimmune optic neuritis by the novel agent fingolimod. *J. Neuroophthalmol.* **33,** 143–8 (2013).

903. Aung, M. H., Kim, M. K., Olson, D. E., Thule, P. M. & Pardue, M. T. Early visual deficits in streptozotocin-induced diabetic long evans rats. *Invest. Ophthalmol. Vis. Sci.* **54,** 1370–7 (2013).

904. Prusky, G. T., Alam, N. M., Beekman, S. & Douglas, R. M. Rapid quantification of adult and developing mouse spatial vision using a virtual optomotor system. *Invest. Ophthalmol. Vis. Sci.* **45,** 4611–6 (2004).

905. Dombeck, D. a, Khabbaz, A. N., Collman, F., Adelman, T. L. & Tank, D. W. Imaging large-scale neural activity with cellular resolution in awake, mobile mice. *Neuron* **56,** 43–57 (2007).

906. Dombeck, D. A., Harvey, C. D., Tian, L., Looger, L. L. & Tank, D. W. Functional imaging of hippocampal place cells at cellular resolution during virtual navigation. *Nat. Neurosci.* **13,** 1433–40 (2010).

907. Ravassard, P. *et al.* Multisensory control of hippocampal spatiotemporal selectivity. *Science* **340,** 1342–6 (2013).

908. Harvey, C. D., Collman, F., Dombeck, D. a & Tank, D. W. Intracellular dynamics of hippocampal place cells during virtual navigation. *Nature* **461,** 941–6 (2009).

909. Hölscher, C., Schnee, A., Dahmen, H., Setia, L. & Mallot, H. A. Rats are able to navigate in virtual environments. *J. Exp. Biol.* **208,** 561–9 (2005).

910. Youngstrom, I. a & Strowbridge, B. W. Visual landmarks facilitate rodent spatial navigation in virtual reality environments. *Learn. Mem.* **19,** 84–90 (2012).

911. Blanchard, R. J., Blanchard, D. C. & Flannelly, K. J. Social stress, mortality and aggression in colonies and burrowing habitats. *Behav. Processes* **11,** 209–213 (1985).

912. Blanchard, R. J. & Blanchard, D. C. Antipredator defensive behaviors in a visible burrow system. *J. Comp. Psychol.* **103,** 70–82 (1989).

913. Blanchard, D. C. *et al.* Visible burrow system as a model of chronic social stress: behavioral and neuroendocrine correlates. *Psychoneuroendocrinology* **20,** 117–34 (1995).

914. Blanchard, R. J., Yudko, E., Dulloog, L. & Blanchard, D. C. Defense changes in stress nonresponsive subordinate males in a visible burrow system. *Physiol. Behav.* **72,** 635–42 (2001).

915. Arakawa, H., Blanchard, D. C. & Blanchard, R. J. Colony formation of C57BL/6J mice in visible burrow system: identification of eusocial behaviors in a background strain for genetic animal models of autism. *Behav. Brain Res.* **176,** 27–39 (2007).

916. Pobbe, R. L. H. *et al.* Expression of social behaviors of C57BL/6J versus BTBR inbred mouse strains in the visible burrow system. *Behav. Brain Res.* **214,** 443–9 (2010).

917. Walk, R. D., Gibson, E. J. & Tighe, T. J. Behavior of light- and dark-reared rats on a visual cliff. *Science* **126,** 80–1 (1957).

918. Fox, M. W. The visual cliff test for the study of visual depth perception in the mouse. *Anim. Behav.* **13,** 232–3 (1965).

919. Eichengreen, J. M., Coren, S. & Nachmias, J. Visual-cliff preference by infant rats: effects of rearing and test conditions. *Science* **151,** 830–1 (1966).

920. Logan, R. W. *et al.* High-precision genetic mapping of behavioral traits in the diversity outbred mouse population. *Genes. Brain. Behav.* **12,** 424–37 (2013).

921. Markham, M. R., Butt, A. E. & Dougher, M. J. A computer touch-screen apparatus for training visual discriminations in rats. *J. Exp. Anal. Behav.* **65,** 173–82 (1996).

922. Bussey, T. J., Muir, J. L. & Robbins, T. W. A novel automated touchscreen procedure for assessing learning in the rat using computer graphic stimuli. *Neurosci. Res. Commun.* **15,** 103–110 (1994).

923. Bussey, T. J. *et al.* The touchscreen cognitive testing method for rodents: how to get the best out of your rat. *Learn. Mem.* **15,** 516–23 (2008).

924. Hager, A. M. & Dringenberg, H. C. Design of a noninvasive face mask for ocular occlusion in rats and assessment in a visual discrimination paradigm. *Behav. Res. Methods* **44,** 919–23 (2012).

925. Passig, C. *et al.* Development of a computerized system for simultaneous visual discrimination in rats. *Behav. Brain Res.* **47,** 199–201 (1992).

926. Levine, T. E., Bornschein, R. L. & Michaelson, I. A. Technique for assessing visual discrimination learning in mice. *Pharmacol. Biochem. Behav.* **7,** 567–70 (1977).

927. Fields, P. E. The efficiency of the serial multiple visual discrimination apparatus and method with white rats. *J. Comp. Physiol. Psychol.* **46,** 69–76 (1953).

928. Prusky, G. T., West, P. W. & Douglas, R. M. Behavioral assessment of visual acuity in mice and rats. *Vision Res.* **40,** 2201–9 (2000).

929. Robinson, L., Bridge, H. & Riedel, G. Visual discrimination learning in the water maze: a novel test for visual acuity. *Behav. Brain Res.* **119,** 77–84 (2001).

930. Prusky, G. T. & Douglas, R. M. Characterization of mouse cortical spatial vision. *Vision Res.* **44,** 3411–8 (2004).

931. Vogel, J. R., Beer, B. & Clody, D. E. A simple and reliable conflict procedure for testing anti-anxiety agents. *Psychopharmacologia* **21,** 1–7 (1971).

932. Millan, M. J. & Brocco, M. The Vogel conflict test: procedural aspects, gamma-aminobutyric acid, glutamate and monoamines. *Eur. J. Pharmacol.* **463,** 67–96 (2003).

933. Basso, A. M., Gallagher, K. B., Mikusa, J. P. & Rueter, L. E. Vogel conflict test: sex differences and pharmacological validation of the model. *Behav. Brain Res.* **218,** 174–83 (2011).

934. Von Frey, M. Untersuchungen über die sinnesfunctionen der menschlichen haut. *Abhandl. Sachs. Ges. Wiss.* **23,** 175–266 (1896).

935. Reeh, P. W. Sensory receptors in mammalian skin in an in vitro preparation. *Neurosci. Lett.* **66,** 141–6 (1986).

936. Andrews, K. The effect of changes in temperature and humidity on the accuracy of von Frey hairs. *J. Neurosci. Methods* **50,** 91–3 (1993).

937. Chaplan, S. R., Bach, F. W., Pogrel, J. W., Chung, J. M. & Yaksh, T. L. Quantitative assessment of tactile allodynia in the rat paw. *J. Neurosci. Methods* **53,** 55–63 (1994).

938. Möller, K. A., Johansson, B. & Berge, O. G. Assessing mechanical allodynia in the rat paw with a new electronic algometer. *J. Neurosci. Methods* **84,** 41–7 (1998).

939. Pitcher, G. M., Ritchie, J. & Henry, J. L. Paw withdrawal threshold in the von Frey hair test is influenced by the surface on which the rat stands. *J. Neurosci. Methods* **87,** 185–93 (1999).

940. Fruhstorfer, H., Gross, W. & Selbmann, O. von Frey hairs: new materials for a new design. *Eur. J. Pain* **5,** 341–2 (2001).

941. Cunha, T. M. *et al.* An electronic pressure-meter nociception paw test for mice. *Braz. J. Med. Biol. Res.* **37,** 401–7 (2004).

942. Lambert, G. A., Mallos, G. & Zagami, A. S. Von Frey's hairs--a review of their technology and use--a novel automated von Frey device for improved testing for hyperalgesia. *J. Neurosci. Methods* **177,** 420–6 (2009).

943. Mills, C. *et al.* Estimating efficacy and drug ED50's using von Frey thresholds: impact of weber's law and log transformation. *J. Pain* **13,** 519–23 (2012).

944. Nirogi, R., Goura, V., Shanmuganathan, D., Jayarajan, P. & Abraham, R. Comparison of manual and automated filaments for evaluation of neuropathic pain behavior in rats. *J. Pharmacol. Toxicol. Methods* **66,** 8–13 (2012).

945. Stewart, C. C. Variations in daily activity produced by alcohol and by changes in barometric pressure and diet, with a description of recording methods. *Am. J. Physiol.* **1,** 40–56 (1898).

946. Holmquest, D. L., Retiene, K. & Lipscomb, H. S. Random light and wheel running. *Science* **154,** 159 (1966).

947. Holmques, D. L., Retiene, K. & Lipscomb, H. S. Circadian rhythms in rats: effects of random lighting. *Science* **152,** 662–4 (1966).

948. Wells, R. L. & Heusner, W. W. A controlled-running wheel for small animals. *Lab. Anim. Sci.* **21,** 904–10 (1971).

949. Porterfield, A. L. & Stern, J. J. A simple method for studying operant wheel running in rats. *Physiol. Behav.* **12,** 1083–5 (1974).

950. Campagna, P. D. A modified voluntary running wheel for laboratory animals. *Lab. Anim. Sci.* **34,** 397–8 (1984).

951. Iversen, I. H. Techniques for establishing schedules with wheel running as reinforcement in rats. *J. Exp. Anal. Behav.* **60,** 219–38 (1993).

952. Sherwin, C. Voluntary wheel running: a review and novel interpretation. *Anim. Behav.* **56,** 11–27 (1998).

953. Deboer, T. & Tobler, I. Running wheel size influences circadian rhythm period and its phase shift in mice. *J. Comp. Physiol. A.* **186,** 969–73 (2000).

954. Banjanin, S. & Mrosovsky, N. Preferences of mice, Mus musculus, for different types of running wheel. *Lab. Anim.* **34,** 313–8 (2000).

955. De Visser, L., van den Bos, R. & Spruijt, B. M. Automated home cage observations as a tool to measure the effects of wheel running on cage floor locomotion. *Behav. Brain Res.* **160,** 382–8 (2005).

956. Siepka, S. M. & Takahashi, J. S. Methods to record circadian rhythm wheel running activity in mice. *Methods Enzymol.* **393,** 230–9 (2005).

957. LeGates, T. A. & Altimus, C. M. Measuring circadian and acute light responses in mice using wheel running activity. *J. Vis. Exp.* 1–9 (2011).

958. Kitsukawa, T. *et al.* A novel instrumented multipeg running wheel system, Step-Wheel, for monitoring and controlling complex sequential stepping in mice. *J. Neurophysiol.* **106,** 479–87 (2011).

959. Dubreucq, S., Marsicano, G. & Chaouloff, F. Emotional consequences of wheel running in mice: which is the appropriate control? *Hippocampus* **21,** 239–42 (2011).

960. Roach, G. C., Edke, M. & Griffin, T. M. A novel mouse running wheel that senses individual limb forces: biomechanical validation and in vivo testing. *J. Appl. Physiol.* **113,** 627–35 (2012).

961. Karvat, G. & Kimchi, T. Systematic autistic-like behavioral phenotyping of 4 mouse strains using a novel wheel-running assay. *Behav. Brain Res.* **233,** 405–14 (2012).

962. Careau, V., Bininda-Emonds, O. R. P., Ordonez, G. & Garland, T. Are voluntary wheel running and open-field behavior correlated in mice? Different answers from comparative and artificial selection approaches. *Behav. Genet.* **42,** 830–44 (2012).

963. Novak, C. M., Burghardt, P. R. & Levine, J. A. The use of a running wheel to measure activity in rodents: relationship to energy balance, general activity, and reward. *Neurosci. Biobehav. Rev.* **36,** 1001–14 (2012).

964. Verwey, M., Robinson, B. & Amir, S. Recording and Analysis of Circadian Rhythms in Running-wheel Activity in Rodents. *J. Vis. Exp.* 1–7 (2013).

965. McNamara, K. C. S., Lisembee, A. M. & Lifshitz, J. The whisker nuisance task identifies a late-onset, persistent sensory sensitivity in diffuse brain-injured rats. *J. Neurotrauma* **27,** 695–706 (2010).

966. Klein, S. M. *et al.* Noninvasive in vivo assessment of muscle impairment in the mdx mouse model--a comparison of two common wire hanging methods with two different results. *J. Neurosci. Methods* **203,** 292–7 (2012).

967. Miquel, J. & Blasco, M. A simple technique for evaluation of vitality loss in aging mice, by testing their muscular coordination and vigor. *Exp. Gerontol.* **13,** 389–96 (1978).

968. Barclay, L. L., Gibson, G. E. & Blass, J. P. The string test: an early behavioral change in thiamine deficiency. *Pharmacol. Biochem. Behav.* **14,** 153–7 (1981).

969. Shepherd, J. K., Grewal, S. S., Fletcher, A., Bill, D. J. & Dourish, C. T. Behavioural and pharmacological characterisation of the elevated "zero-maze" as an animal model of anxiety. *Psychopharmacology (Berl).* **116,** 56–64 (1994).

970. Cook, M. N., Crounse, M. & Flaherty, L. Anxiety in the elevated zero-maze is augmented in mice after repeated daily exposure. *Behav. Genet.* **32,** 113–8 (2002).

971. Kulkarni, S. K., Singh, K. & Bishnoi, M. Elevated zero maze: a paradigm to evaluate antianxiety effects of drugs. *Methods Find. Exp. Clin. Pharmacol.* **29,** 343–8 (2007).

972. Milner, L. C. & Crabbe, J. C. Three murine anxiety models: results from multiple inbred strain comparisons. *Genes. Brain. Behav.* **7,** 496–505 (2008).

973. Heredia, L., Torrente, M., Domingo, J. L. & Colomina, M. T. Individual housing and handling procedures modify anxiety levels of Tg2576 mice assessed in the zero maze test. *Physiol. Behav.* **107,** 187–91 (2012).

974. Faraji, J., Lehmann, H., Metz, G. A. & Sutherland, R. J. Rats with hippocampal lesion show impaired learning and memory in the ziggurat task: a new task to evaluate spatial behavior. *Behav. Brain Res.* **189,** 17–31 (2008).

975. Faraji, J., Metz, G. A. & Sutherland, R. J. Characterization of spatial performance in male and female Long-Evans rats by means of the Morris water task and the ziggurat task. *Brain Res. Bull.* **81,** 164–72 (2010).
